# Tissue-Specific Downregulation of Fatty Acid Synthase Suppresses Intestinal Adenoma Formation via Coordinated Reprograming of Transcriptome and Metabolism in the Mouse Model of Apc-Driven Colorectal Cancer

**DOI:** 10.3390/ijms23126510

**Published:** 2022-06-10

**Authors:** James Drury, Lyndsay E. A. Young, Timothy L. Scott, Courtney O. Kelson, Daheng He, Jinpeng Liu, Yuanyan Wu, Chi Wang, Heidi L. Weiss, Teresa Fan, Matthew S. Gentry, Ramon Sun, Yekaterina Y. Zaytseva

**Affiliations:** 1 Department of Toxicology and Cancer Biology, University of Kentucky, Lexington, KY 40536, USA; james.drury12@uky.edu (J.D.); courtney.kelson@uky.edu (C.O.K.); teresa.fan@uky.edu (T.F.); 2 Department of Molecular and Cellular Biochemistry, University of Kentucky, Lexington, KY 40536, USA; lyndsay.young@uky.edu (L.E.A.Y.); matthew.gentry@uky.edu (M.S.G.); 3 Markey Cancer Center, University of Kentucky, Lexington, KY 40536, USA; tim.scott@uky.edu (T.L.S.); ramon.sun@uky.edu (R.S.); 4 Center for Environmental and Systems Biochemistry, University of Kentucky, Lexington, KY 40536, USA; 5 Markey Cancer Center Biostatistics and Bioinformatics Shared Resource Facility, University of Kentucky, Lexington, KY 40536, USA; daheng.he@uky.edu (D.H.); jinpeng.liu@uky.edu (J.L.); ywu244@g.uky.edu (Y.W.); chi.wang@uky.edu (C.W.); heidi.weiss@uky.edu (H.L.W.); 6 Department of Neuroscience, University of Kentucky, Lexington, KY 40536, USA

**Keywords:** colorectal cancer, fatty acid synthase, lipid metabolism, colorectal cancer initiation, Apc mutation

## Abstract

Altered lipid metabolism is a potential target for therapeutic intervention in cancer. Overexpression of Fatty Acid Synthase (FASN) correlates with poor prognosis in colorectal cancer (CRC). While multiple studies show that upregulation of lipogenesis is critically important for CRC progression, the contribution of FASN to CRC initiation is poorly understood. We utilize a C57BL/6-Apc/Villin-Cre mouse model with knockout of FASN in intestinal epithelial cells to show that the heterozygous deletion of FASN increases mouse survival and decreases the number of intestinal adenomas. Using RNA-Seq and gene set enrichment analysis, we demonstrate that a decrease in FASN expression is associated with inhibition of pathways involved in cellular proliferation, energy production, and CRC progression. Metabolic and reverse phase protein array analyses demonstrate consistent changes in alteration of metabolic pathways involved in both anabolism and energy production. Downregulation of FASN expression reduces the levels of metabolites within glycolysis and tricarboxylic acid cycle with the most significant reduction in the level of citrate, a master metabolite, which enhances ATP production and fuels anabolic pathways. In summary, we demonstrate the critical importance of FASN during CRC initiation. These findings suggest that targeting FASN is a potential therapeutic approach for early stages of CRC or as a preventive strategy for this disease.

## 1. Introduction

Currently ranked as the second leading cause of cancer-related deaths in the United States, colorectal cancer (CRC) remains a substantial public health problem with an estimated 149,500 new cases and 52,980 deaths during 2021 (https://www.cancer.org/cancer/colon-rectal-cancer/about/key-statistics.html, accessed on 31 July 2021). Abnormally elevated lipid synthesis provides cancer cells with membrane building blocks, signaling lipid molecules, posttranslational modifications of proteins, and energy supply to support rapid cell proliferation [1,2,3]. Fatty Acid Synthase (FASN), a key enzyme of de novo lipid synthesis, has been actively investigated as a therapeutic target in cancer. FASN is the most targetable among lipogenesis genes due to its high degree of overexpression in cancer cells [1,4,5]. Multiple studies, including reports from our laboratory [1,6,7,8], have found that elevated expression of FASN is associated with advanced stages of CRC and CRC metastasis [6,7]. Pre-clinical studies show significant anti-cancer effects when lipid synthesis is inhibited by genetic and pharmacological inhibition of FASN [9,10,11]. Currently, a novel FASN inhibitor, TVB-2640, is being tested in one Phase I and two Phase II clinical trials [12]. Even though numerous studies show the benefit of targeting FASN in cancer including CRC, knowledge about the contribution of lipid synthesis to CRC initiation is very limited, and the utility of this pathway as a therapeutic target for the early stages of this disease is unclear.

Multiomics-based analyses of paired normal and tumor tissues from 275 patients with colorectal cancer revealed that metabolic alterations occur at the adenoma stage of carcinogenesis [13]. Several studies have shown that FASN is significantly upregulated in the early stages of CRC [6,13,14,15]. Increased expression of FASN in 86% of aberrant crypt foci from patients with sporadic CRC or familial adenomatous polyposis also suggests its importance in the early stages of colonic neoplasm development [16]. Consistently, FASN is significantly overexpressed in rectal biopsies from patients harboring adenomas compared with those with no adenomas [17].

A recent study using mouse embryonic fibroblasts transfected with polyomavirus middle T antigen (PyMT), a breast cancer oncoprotein, provides strong evidence that FASN plays an important role in the initial step of cell transformation and is required for cancer cells to acquire 3D growth properties during transformation [18]. FASN deletion results in low rates of glycolysis and mitochondrial respiration as well as an accumulation in reactive oxygen species [18], suggesting that upregulation of fatty acid synthesis promotes tumorigenesis via alteration of metabolic pathways and redox status. This study further emphasizes the potential importance of utilizing FASN as a therapeutic target for the prevention and treatment of early stages of cancer. However, the feasibility of targeting FASN as a preventive strategy or early-stage treatment in CRC has not been explored.

Epidemiological studies demonstrate that diet and dietary fatty acids can contribute to CRC initiation and development [19,20]. Whereas most tumors are dependent on fatty acid synthesis, they can also scavenge lipids from their environment [1]. We have shown that upregulation of CD36, a fatty acid transporter, and an increase in exogenous lipid uptake can compensate for the effect of pharmacological or genetic inhibition of FASN [21]. Therefore, to identify FASN-mediated vulnerabilities which can be efficiently targeted in cancer, it is very important to understand the contribution of FASN to CRC initiation in the context of the in vivo in a mouse model with ad libitum feeding. Mouse models carrying mutations in the *Apc* (adenomatosis polyposis coli) gene are genetically parallel to familial and sporadic colon adenoma development in humans [22]. The alteration of FASN in this model provides a unique opportunity to better understand the contribution of this enzyme and de novo lipid synthesis to Apc-driven carcinogenesis.

We discovered that heterozygous deletion of FASN significantly increases mouse survival and decreases the number of intestinal adenomas formed. Analysis of gene set enrichment data revealed a decrease in FASN expression leading to a significant decrease in the enrichment of genes associated with pathways involved in cellular proliferation, energy production, and CRC progression. In agreement with these data, a decrease in FASN expression reduces the levels of metabolites involved in glycolysis and the tricarboxylic acid (TCA) cycle with the most significant reduction in the level of cellular citrate, a metabolite involved in ATP production and fueling of anabolic pathways. Using the reverse phase protein array (RPPA), we demonstrate alterations in the protein levels of multiple metabolic enzymes. Interestingly, the levels of diglycerides in adenomas and free fatty acids in adenomas and plasma are not affected by the changes in FASN expression in the intestine.

In summary, this study provides strong evidence that FASN is critically important in CRC initiation by orchestrating changes in the transcriptome and metabolic pathways consistent with an increase in proliferation, ATP production, and anabolism. Therefore, targeting FASN should be further explored as a potential preventive strategy or early-stage treatment for CRC.

## 2. Results

### 2.1. Heterozygous Deletion of FASN Increases Survival and Decreases the Number of Intestinal Adenomas during Apc-Driven Carcinogenesis

**Figure 1:**
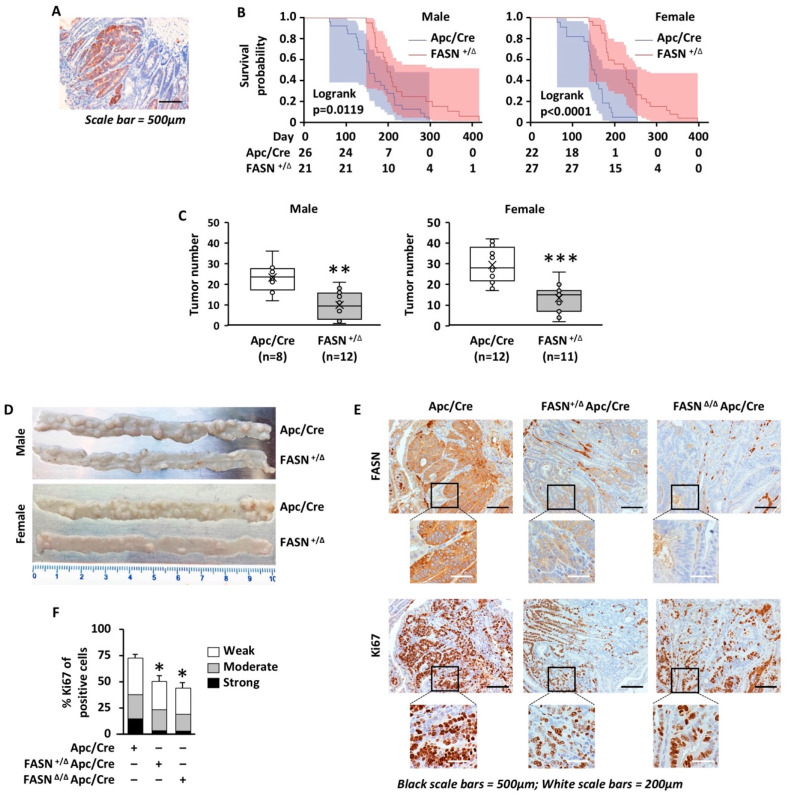
Heterozygous deletion of FASN increases mice survival and decreases the number of adenomas formed in the Apc-driven carcinogenesis mouse model. (**A**) Immunohistochemistry staining for FASN in intestinal tissues from Apc/Cre mice. (**B**) The effect of Villin-Cre-mediated heterozygous deletion of FASN in mouse intestinal tissues on mice survival. (**C**) The effect of Villin-Cre-mediated heterozygous deletion of FASN in mouse intestinal tissues on formation of mouse adenomas. Number of adenomas was quantified within 10 cm sections of distal intestine from Apc/Cre mice and mice with heterozygous deletion of FASN. (**D**) Representative images of intestinal tissues from male and female mice, Apc/Cre vs. Apc/Cre with heterozygous deletion of FASN. (**E**) Representative images of immunochemistry staining for FASN and Ki67 in Apc/Cre mice and Apc/Cre mice with hetero- and homozygous deletion of FASN. (**F**) Quantification of Ki67 staining in mouse adenomas with the different levels of FASN expression. (* *p* < 0.05, ** *p* < 0.01, *** *p* < 0.001).

We utilized a C57BL/6-*Apc^tm1Tyj^* mouse model in which the 15 coding exons of the *Apc* gene are flanked by LoxP sites. Germline heterozygous deletion of the floxed region results in a mouse highly suspectable to spontaneous intestinal adenoma formation and serves as a mouse model of CRC. Immunohistochemistry staining of intestinal tissues from Apc/Villin-Cre (Apc/Cre) mice demonstrates high expression of FASN in adenomas as compared to surrounding tissues (Figure 1A). These mice were bred with mice that have LoxP-flanked FASN alleles [23] to establish mouse colonies with heterozygous (FASN^+/∆^/Apc/Cre) and homozygous (FASN^∆/∆^/Apc/Cre) deletion of FASN in the intestinal epithelium (Figure S1A) [21]. Consistent with a previously published study, homozygous deletion of FASN in the intestine leads to a smaller size litter and premature death of approximately 70–80% of animals within 2 months after birth due to disruptions in the intestinal mucus barrier [24]. However, approximately 20% of FASN^∆/∆^/Apc/Cre mice survive and have a phenotype similar to FASN^+/∆^/Apc/Cre mice (Figure S1B–D). Immunohistochemistry staining demonstrates residual expression of FASN in these mice (Figure S1E,F), potentially due to the inefficiency of the *Cre* transgene [25], which would explain the phenotype similar to FASN^+/∆^/Apc/Cre mice. We previously demonstrated that hetero- and homozygous deletion of FASN in Apc/Cre mice lead to the upregulation of CD36, a fatty acid transporter [21]. Indeed, immunohistochemistry staining reveals a high level of CD36 expression in FASN^∆/∆^/Apc/Cre intestinal mucosa, suggesting that a potential compensation of inhibited lipid synthesis is increased fatty acid uptake (Figure S1G). Due to the variability in FASN expression and survival in FASN^∆/∆^/Apc/Cre group, these animals were omitted from the survival analysis and studies evaluating the number of intestinal adenomas.

To investigate the contribution of FASN to survival and adenoma formation, we used Apc/Cre and FASN^+/∆^/Apc/Cre mice kept on standard laboratory chow. The heterozygous deletion of FASN significantly increases mouse survival (Figure 1B) and decreases the number of intestinal adenomas in both male and female mice (Figure 1C,D). There were no significant changes in animal size due to differences in FASN expression (Figure S1B,C). We noted that female mice develop a higher number of adenomas and have shorter survival as compared to male mice, but the differences between genders were not statistically significant (Figure 1B,C). Even though we excluded FASN^∆/∆^/Apc/Cre mice from analysis, we noted that the higher degree of FASN inhibition in this group is associated with a much lower number of intestinal adenomas. Several mice in this group were adenoma-free and survived over a year until they were sacrificed for analysis (Figure S1B,C).

Ki-67 expression has been widely used in clinical practice as an index to evaluate the proliferative activity of tumor cells [26]. The sections of the intestine from Apc/Cre, FASN^+/∆^/Apc/Cre, and FASN^∆/∆^/Apc/Cre mice were prepared using the swiss roll technique, and immunohistochemistry staining for Ki67 was performed. The analysis of Ki67 staining using the HALO digital pathology analysis platform revealed high expression of FASN in Apc/Cre adenomas is associated with a higher percentage of Ki67 positive cells and higher intensity staining as compared to FASN^+/∆^/Apc/Cre and FASN^∆/∆^/Apc/Cre adenomas (Figure 1E,F), suggesting a higher proliferative capability of these adenomas.

Together, these data suggest that FASN promotes CRC initiation by increasing the number of adenomas formed and the proliferation of CRC cells, thus decreasing mouse survival.

### 2.2. A Decrease in FASN Expression Is Associated with Downregulation of Pathways Linked to Cellular Proliferation, Energy Production, and Cancer-Associated Signaling

**Figure 2:**
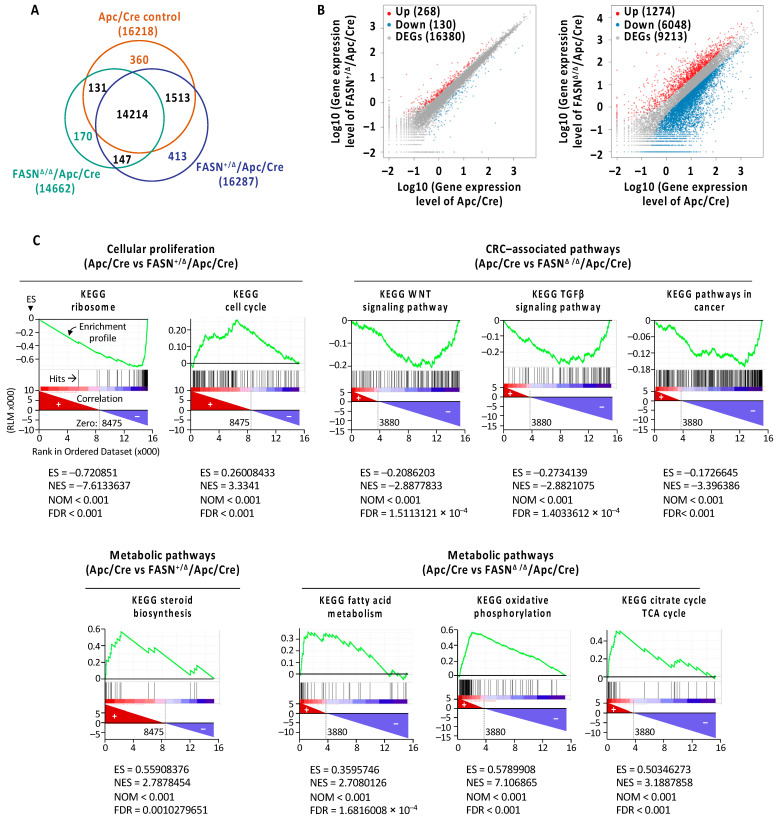
FASN promotes adenoma formation via pathways upregulation of pathways involved in cell growth and energy metabolism. (**A**) Venn diagram displaying the overlapping genes identified in tumors from Apc/Cre mice and Apc/Cre mice with heterozygous and homozygous expression of FASN. (**B**) Scatter plots showing the numbers of differentially expressed genes in Apc/Cre mice and Apc/Cre mice with heterozygous and homozygous expression of FASN. Significantly changed DEGs are indicated in colors. Red and blue dots are up- and downregulated genes, respectively. The detailed lists of differentially expressed genes are provided in Table S2A. (**C**) Representative gene set enrichment analysis plots generated from RNA-Seq expression data of Apc/Cre and FASN knockout mice. The bar codes indicate the location of the members of the gene set in the ranked list of all genes. ES, enrichment score; NES, normalized enrichment score; NOM, nominal *p*-value; FDR, false discovery rate adjusted *p*-value.

To determine the effect of FASN on gene expression profile during Apc-driven carcinogenesis, we performed RNA-Seq analysis on adenomas collected from Apc/Cre, FASN^+/∆^/Apc/Cre and FASN^∆/∆^/Apc/Cre mice (Table S1A,B). Venn diagram shows numbers of overlapping and non-overlapping genes differentially expressed among three different genotypes (Figure 2A).

Deletion of FASN resulted in significant transcriptome changes with exacerbated changes observed in FASN^∆/∆^/Apc/Cre mice compared to FASN^∆/−^/Apc/Cre mice (Figure 2B, Table S1C-1,C-2). Gene set enrichment analysis shows that high FASN expression in adenomas from Apc/Cre mice is associated with enrichment of genes associated with cellular proliferation, energy production, and oncogenic signaling as compared to adenomas collected from mice with hetero- and homozygous deletion of FASN (Figure 2C). Lists of the top 20 positively and negatively enriched pathways and genes associated with downregulation of FASN in FASN^+/∆^/Apc/Cre and FASN^∆/∆^/Apc/Cre mice are included in Table S1D-1–D-4.

In summary, these data further confirm that FASN promotes adenoma formation via altered expression of genes involved in proliferation, energy production, and CRC progression.

### 2.3. Heterozygous Deletion of FASN Alters the Levels of Diglycerides, but Does Not Change the Total Levels of Free Fatty Acid and Sphingolipid Species in Mouse Adenomas

**Figure 3:**
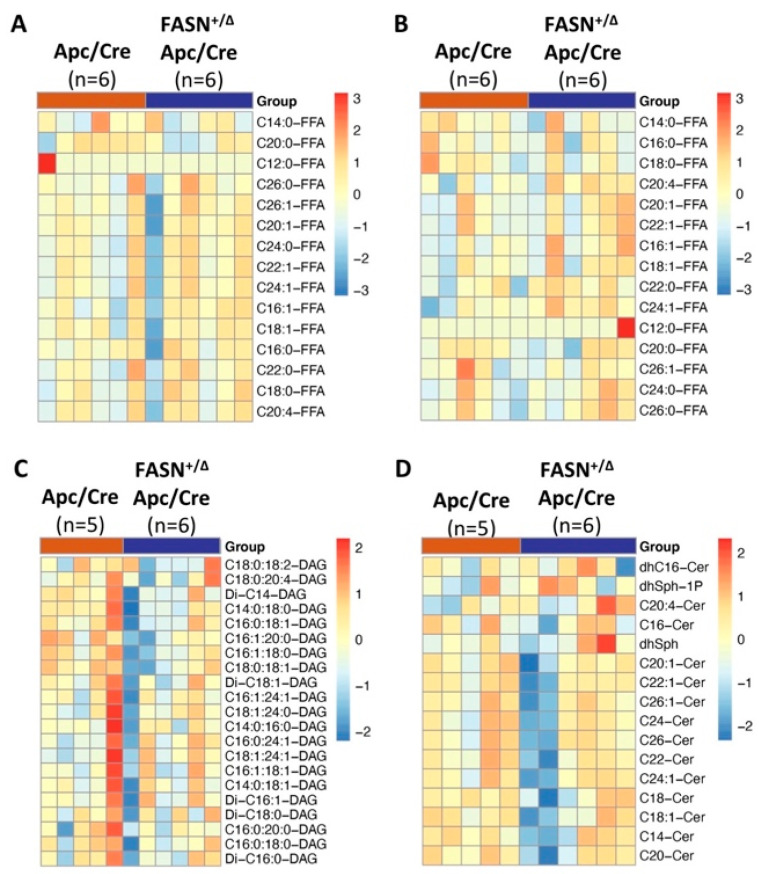
The effect of heterozygous deletion of FASN on lipid composition. Heat maps showing the composition of free fatty acids in (**A**) adenoma tissues and (**B**) plasma, and the composition of (**C**) diglycerides and (**D**) sphingolipids in mouse adenomas from Apc/Cre and FASN^+/∆^ Apc/Cre mice.

We have shown that the shRNA-mediated knockdown of FASN abolishes de novo lipid synthesis but does not affect the total palmitate level in established CRC cell lines [6]. Adenoma tissues and plasma from Apc/Cre and FASN^+/∆^/Apc/Cre mice were analyzed to determine the levels of free fatty acids. Due to the limited number of FASN^∆/∆^/Apc/Cre mice available and the low number of adenomas formed in this genotype group, we did not perform lipid analysis for this genotype. As shown in Figure 3A,B and Table S2A,B, we did not find any significant differences in the levels of free fatty acids between Apc/Cre and FASN^+/∆^/Apc/Cre mice. Our previous studies suggest that FASN activity regulates lipid storage and de novo sphingolipid synthesis [8,27]. Therefore, we measured the total levels of diglycerides and sphingolipids in adenomas from Apc/Cre and FASN^+/∆^/Apc/Cre mice. We observed a significant decrease in the levels of some diglycerides (C16:1:20:0-DAG; Di-C14-DAG; C14:0:18:0-DAG; C16:1:18:0-DAG; C18:0:18:1-DAG, *p*-value < 0.05) (Figure 3C and Table S2C). However, we would like to note that further statistical analysis and adjustments for the false discovery rate (q-value) show no statistical significance. No significant changes were seen in the total levels of sphingolipids (Figure 3D and Table S2D). We have also analyzed the levels of triglycerides in adenoma tissue and plasma of Apc/Cre, FASN^+/∆^/Apc/Cre, and FASN^∆/∆^/Apc/Cre using a triglyceride quantification kit. As shown in Figure S2A,B, no significant differences were observed between Apc/Cre mice and Apc/Cre mice with hetero- and homozygous deletion of FASN. Together, these data suggest that the heterozygous deletion of FASN primarily alters the levels of diglycerides but does not significantly affect the total level of free fatty acids and sphingolipids in mouse adenomas.

### 2.4. Downregulation of FASN in Mouse Adenomas Alters the Levels of Cellular Metabolites

**Figure 4:**
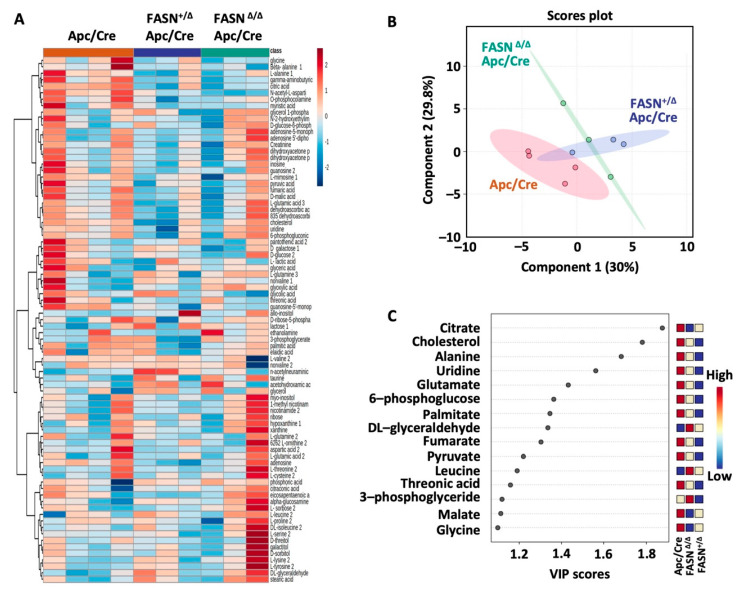
Downregulation of FASN is associated with alteration in multiple metabolic intermediates in mouse adenomas. (**A**) Heat map demonstrating the levels of metabolites (log normalization) identified in adenoma tissues from Apc/Cre (*n* = 4) and Apc/Cre mice with hetero-(*n* = 3) and homozygous (*n* = 3) deletion of FASN. (**B**) Partial least-squares discriminant analysis (PLS-DA) on metabolic data from Apc/Cre, FASN^+/∆^ Apc/Cre, and FASN ^∆/∆^ Apc/Cre mice. (**C**) Variable importance in projection (VIP) values from PLS-DA. The VIP score of a metabolite is calculated as a weighted sum of the squared correlations between the PLS-DA components and the original variable (FASN expression). The *x*-axis indicates the VIP scores corresponding to each metabolite on the *y*-axis.

Our previous studies demonstrated that FASN regulates glycolysis and mitochondrial respiration in vitro [8]. To assess FASN-mediated changes of metabolites in glycolysis and the TCA cycle, we performed a metabolic analysis of adenomas from Apc/Cre, FASN^+/∆^/Apc/Cre, and FASN^∆/∆^/Apc/Cre mice using GC-MS. Polar metabolites were extracted from pulverized tumors of the three genotypes and metabolites were identified. Metabolites from all major pathways were detected, including glycolysis and TCA cycle intermediates, lipids, sugars, amino acids, and others. Using this information-rich dataset, supervised clustering analysis was performed to assess overall metabolic profiles for each cohort. The heat map of these data demonstrated significant changes in the metabolite levels among Apc/Cre, FASN^+/∆^/Apc/Cre, and FASN^∆/∆^/Apc/Cre (Figure 4A). The PLS-DA further demonstrates a significant difference in the metabolic profiles of adenomas among Apc/Cre, FASN^+/∆^/Apc/Cre, and FASN^∆/∆^/Apc/Cre mice (Figure 4B). Based on VIP score, citric acid, cholesterol, alanine, uridine, glutamate, 6-phosphoglucose, and palmitate were identified as the top metabolic intermediates that contribute to FASN-driven differences observed among mice genotypes (Figure 4C).

Taken together, these data demonstrate that FASN upregulation is associated with metabolic pathways involved in the turnover of citrate, palmitate, cholesterol, and 6-phosphoglucose, as well as in the synthesis of uridine and amino acids such as alanine and glutamate.

### 2.5. Deletion of FASN Alters Expression of Metabolites and Their Metabolizing Enzymes in Adenomas

**Figure 5:**
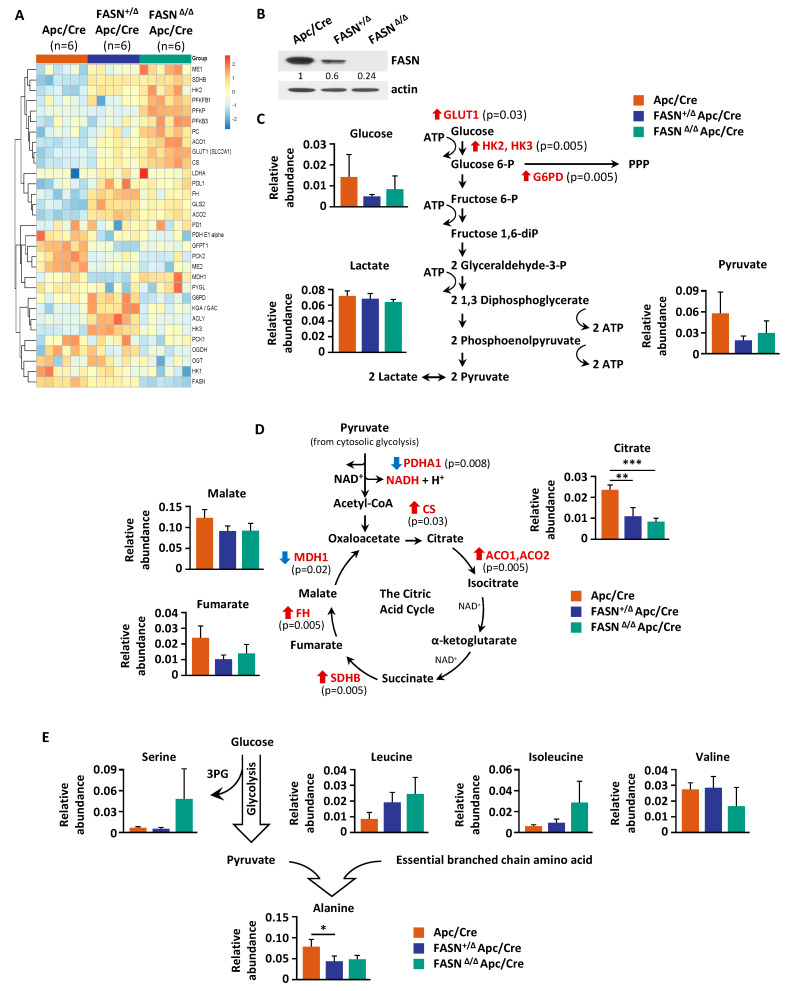
FASN knockdown alters the levels of metabolites and metabolic enzymes involved in glycolysis, TCA cycle, and amino acid metabolism. (**A**) Heat map demonstrating expression of metabolic enzymes as determined by Reverse Phase Protein Array (RPPA) analysis. (**B**) The level of FASN expression by western blot of mouse adenomas used for RPPA analysis. (**C**) FASN-mediated changes in metabolites and metabolizing enzymes within glycolysis. (**D**) FASN-mediated changes in metabolites and metabolizing enzymes within the TCA cycle. (**E**) Hetero- and homozygous knockdown of FASN alters the levels of branched-chain amino acids and decreases synthesis of alanine. (* *p* < 0.05, ** *p* < 0.01, *** *p* < 0.001).

To profile the expression of metabolic enzymes within glycolysis and the TCA cycle, we performed reverse phase protein array (RPPA) [28]. The heat map of the RPPA analysis shows the levels of metabolic enzyme expression in adenomas collected from Apc/Cre, FASN^+/∆^/Apc/Cre, and FASN^∆/∆^/Apc/Cre mice (Figure 5A). The expression of FASN in samples analyzed by RPPA is also shown by western blot (Figure 5B). Interestingly, we observed an increase in the expression of glucose transporter 1 and glycolytic enzyme hexokinases 2 and 3. The expression of glucose-6-phosphate dehydrogenase (G6PD), a rate-limiting enzyme of the pentose phosphate pathway is also increased (Figure 5A,C and Table S3). In contrast, the enzymes glutamine-fructose-6-phosphate transaminase 1 and O-linked N-acetylglucosamine transferase, which are involved in hexosamine synthesis, a branch of glycolysis and O-linked glycosylation, significantly decreased (Figure 5A and Table S3). The decrease in the levels of glucose in adenomas from FASN^+/∆^/Apc/Cre and FASN^∆/∆^/Apc/Cre mice suggests that glycolytic enzymes may be upregulated due to the limited substrate availability (Figure 5C).

Interestingly, even though we did not observe a change in the level of lactate, levels of pyruvate and glucose trended lower in FASN^+/∆^/Apc/Cre and FASN^∆/∆^/Apc/Cre mice as compared to Apc/Cre mice (Figure 5C). Pyruvate dehydrogenase E1 subunit alpha 1, a component of the pyruvate dehydrogenase enzyme complex, links glycolysis and the TCA cycle and is important for cancer metabolic shift [29]. Strikingly, we observed significant downregulation of this enzyme in adenomas from FASN^+/∆^/Apc/Cre and FASN^∆/∆^/Apc/Cre mice as compared to Apc/Cre mice (Figure 5A,C and Table S3).

Nicotinamide adenine dinucleotide phosphate (NADPH) is produced by metabolic enzymes such as G6PD and 6-phosphogluconate dehydrogenase (6PGD) of the pentose phosphate pathway, malic enzymes (MEs), isocitrate dehydrogenases, and enzymes in one-carbon-tetrahydrofolate oxidation pathways [30]. Intriguingly, we found the ME1, a cytosolic NADP^+^-dependent isoform, is upregulated in FASN^+/∆^/Apc/Cre and FASN^∆/∆^/Apc/Cre mice as compared to Apc/Cre mice, and ME2, a mitochondrial NAD^+^-dependent isoform, is significantly downregulated (Figure 5A and Table S3). ME1 plays important role in generating NADPH for lipid and cholesterol synthesis and increases FASN expression in the intestine, suggesting that upregulation of ME1 may be a potential compensation mechanism due to a decrease in lipid synthesis.

The TCA cycle constitutes the epicenter of cell metabolism because it oxidizes multiple substrates while providing precursors for the synthesis of lipids, nucleotides, and proteins [31]. The TCA cycle begins with the reaction that combines the two-carbon acetyl-CoA with a four-carbon oxaloacetate to generate the six-carbon citrate [31]. We found that the level of citrate is significantly decreased in both FASN^+/∆^/Apc/Cre and FASN^∆/∆^/Apc/Cre mice as compared to Apc/Cre mice (Figure 5D). Furthermore, according to the PLS-VIP analysis, citrate has the highest VIP score (see Figure 4C), suggesting its significance in FASN-mediated metabolic changes in our genetically modified mouse models. We noted that other TCA cycle substrates such as malate and fumarate are also decreased in FASN^+/∆^/Apc/Cre and FASN^∆/∆^/Apc/Cre mice as compared to Apc/Cre mice, but these changes did not reach statistical significance (Figure 5D).Even though the level of citrate significantly decreased, the expression of CS increased, potentially due to feedback regulation by the availability of the precursor pyruvate (thus acetyl CoA) (Figure 5A,C and Table S3). We confirmed that knockout of FASN leads to upregulation of CS in mouse tissues using western blot analysis (Figure S3). Intriguingly, the level of mitochondrial isoform phosphoenolpyruvate carboxykinase 2, which converts oxaloacetate into phosphoenolpyruvate, is significantly decreased due to FASN downregulation, suggesting a decrease in utilization of non-glucose substrates such as glutamine to fuel the pentose phosphate pathway and serine/glycine metabolism [32].

Amino acids play diverse roles in cancer cells, supporting biosynthetic pathways, redox balance, epigenetic regulation, and immune responses [33,34]. Alanine can be synthesized from pyruvate and branched-chain amino acids (BCAA) such as valine, leucine, and isoleucine [35] and plays an important role in the synthesis of proteins, amino acids, and other biomolecules as well as energy fuel for cancer cells [36]. We found that both the hetero- and homozygous deletion of FASN increase the levels of leucine and isoleucine, and significantly decrease the level of alanine in intestinal adenomas (Figure 5E), suggesting the potential impairment in BCAA metabolism.

Cumulatively, these data suggest that a decrease in FASN expression is associated with a decrease in metabolic intermediates of the TCA cycle with the most significant change in the level of intracellular citrate, which is a key metabolite supporting mitochondrial ATP production and anabolic reactions.

### 2.6. Expression of CS Is Upregulated and Correlates with Expression of FASN in Colorectal Cancer

**Figure 6:**
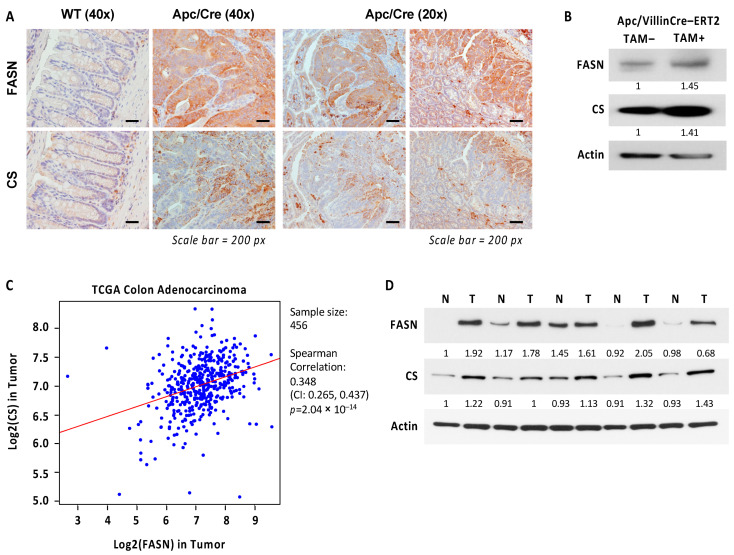
Expression of FASN correlates with expression of CS in CRC. (**A**) Expression of FASN and CS in intestinal tissues of wild type (WT) C57BL/6J mice and in intestinal tissues and adenomas in Apc/Cre mice. (**B**) Heterozygous deletion of *Apc* gene using tamoxifen (TAM) inducible Villin-Cre recombinase leads to upregulation of FASN and CS in mouse intestinal tissues. Tamoxifen was injected for 5 days, and intestinal tissues were collected on day 10 after the last injection. (**C**) Correlations between FASN and CS were determined based on RNA-Seq data of CRC patient tumor tissues (*n* = 456) from The Cancer Genome Atlas. (**D**) Expression of FASN and CS in human normal colon mucosa and matched tumor tissues. N-normal mucosa, T-primary tumor.

CS activation drives flux toward lipid and triglyceride synthesis in cancer [37]. Since FASN knockout upregulates CS expression in transgenic mouse models, we sought to evaluate the levels of FASN and CS expression in mouse tissues during Apc-driven carcinogenesis. Immunohistochemistry staining of FASN and CS revealed that both are significantly upregulated in intestinal epithelium and adenomas in Apc/Cre mice as compared to intestinal tissues of wild-type C57BL/6J mice (Figure 6A). We noted that even though the expression pattern of CS does not always recapitulate that of FASN, their expression seems to localize in the same areas of developing adenomas (Figure 6A). Interestingly, the inactivation of *Apc* gene using tamoxifen-inducible Villin-Cre-ERT2 resulted in upregulation of both FASN and CS expression in the intestinal mucosa (Figure 6B). To translate our findings to human cancer, we analyzed The Cancer Genome Atlas RNA-Seq data and identified a highly significant positive correlation between FASN and CS gene expression in human colon adenocarcinomas (Figure 6C). In agreement with these data, western blot analysis of fresh normal human colon mucosa and matched primary tumors demonstrates an increase in FASN and CS in tumors as compared to normal tissues (Figure 6D).

In summary, these data suggest that CS is highly expressed in CRC, and there is a positive correlation between CS and FASN expression at mRNA and protein levels in colon adenocarcinomas.

## 3. Discussion

Aberrant lipid synthesis via upregulation of FASN is crucial for cancer cells, and targeting FASN can be a potential therapeutic strategy in many cancers, including CRC [1,3]. Even though multiple studies demonstrate that inhibition of FASN decreases CRC cell growth and survival in vitro, pre-clinical studies demonstrate much less efficacy of FASN inhibition on tumor growth in vivo, potentially due to compensation mechanisms such as dietary fatty acid uptake [9,21]. A better understanding of the timing and conditions for using FASN-targeted therapy is critical for developing successful therapeutic strategies [4,9,10]. A recently published study suggests that FASN activity could be essential during the initial steps of the transformation process and, thus, can be a target for cancer prevention [18]. However, the contribution of FASN to CRC initiation has not been extensively studied. Therefore, the goal of this study was to understand the role of FASN in Apc-driven carcinogenesis and to evaluate it as a potential target for CRC prevention using transgenic mouse models.

Our study is the first to elucidate the effect of Villin-Cre-mediated downregulation of FASN expression in intestinal epithelial cells on mice survival, adenoma formation, and transcriptome and metabolome of adenomas in the transgenic model of Apc-driven CRC. The results of our study show that heterozygous deletion of FASN in Apc/Cre mice significantly increases mouse survival and decreases the number of intestinal adenomas. These results are consistent with the study showing that inhibition of FASN by orlistat, a drug used for treating obesity [38], increases survival rates in Apc^Min^ mice, a commonly used model for Apc-driven CRC [39]. Another study using pharmacological inhibitors in Apc^Min^ mice showed that orlistat and lovastatin, inhibitors of cholesterol biosynthesis, significantly reduced FASN enzymatic activities and gene expression in colonic tissues. However, they did not affect the number of intestinal polyps and there was a statistically significant reduction in polyp volume only in the mouse group treated with lovastatin [40]. The discrepancy in outcomes of these studies is potentially due to different diets and different doses of orlistat used [39,40]. Our results showing that FASN promotes carcinogenesis are also in agreement with studies on other types of cancer, showing that transgenic expression of FASN results in a significant increase in prostate intraepithelial neoplasia [41] and that pharmacological inhibition of FASN with Fasnall [42] or C75 [43] significantly delays tumor progression in *neu*-N mice, a model of mammary cancer.

The meta-analysis of 34 studies and 6180 CRC patients demonstrates that high expression of Ki67, a proliferation marker, is significantly correlated with poor overall survival and disease-free survival [44]. Indeed, our results demonstrate that high expression of FASN in Apc/Cre mice is associated with a higher percentage of Ki67 positive cells. Conversely, downregulation of FASN expression in FASN^+/∆^/Apc/Cre and FASN^∆/∆^/Apc/Cre is associated with a decrease in the percentage of Ki67 positive cells and the intensity of Ki67 staining, suggesting that high expression of FASN is associated with a higher proliferative activity of CRC cells. These findings are further supported by the GSEA analysis of RNA-Seq data on adenomas from Apc/Cre, FASN^+/∆^/Apc/Cre, and FASN^∆/∆^/Apc/Cre mice showing the significant enrichment of genes involved in cell cycle progression in Apc/Cre mice as compared to adenomas from mice with hetero- and homozygous deletion of FASN.

Our data from transgenic mice demonstrate that FASN significantly upregulates the set of genes associated with the pathways involved in the cell cycle, steroid biosynthesis, and metabolism. These results are in agreement with data obtained from in vitro studies on human CRC cells showing that similar pathways are modulated by TVB-3166, a FASN inhibitor and an analog of TVB-2640, which is currently used in clinical trials [10,45]. Together, the data further confirm the specificity of TVB inhibitors in targeting FASN in cancer cells. Interestingly, we also found that gene expression of several stem cell markers implicated in CRC, such as LGR5, ALDH, CD44, and CD166, is significantly downregulated in FASN knockout adenomas, suggesting that FASN may promote APC-driven carcinogenesis via an increase in stemness of intestinal epithelial cells. This mechanism is currently under investigation in our laboratory.

Our previous studies demonstrated that genetic and pharmacological inhibition of FASN is associated with the inhibition of glycolysis, TCA cycle activity, and beta-oxidation in vitro [8]. Consistently, low respiration and glycolytic capacity were observed when FASN was deleted in MEFs infected with retroviral particles expressing the PyMT breast cancer oncogene (FASN^∆/∆^-PyMT) as compared to control FASN^lox/lox^-PyMT MEFs [18]. In agreement with these studies, our GSEA results show reduced expression of genes involved in the TCA cycle and beta-oxidation in FASN^+/∆^/Apc/Cre and FASN^∆/∆^/Apc/Cre as compared to Apc/Cre mice. Interestingly, heterozygous deletion of FASN leads to a drastic and significant decrease in the expression of genes associated with the cell cycle and ribosome pathways, suggesting that an approximate 50% decrease in FASN expression in these mice is sufficient to significantly inhibit cellular proliferation and protein synthesis. Steroid biosynthesis is also significantly downregulated in these mice as compared to Apc/Cre control mice. The higher extent of FASN downregulation in mice with homozygous deletion leads to additional alterations in gene expression associated with inhibition of energy production, fatty acid biosynthesis, and CRC-promoting pathways, suggesting that a higher degree of FASN inhibition elicits a more efficient and global antitumor effect.

Our previous work demonstrated that shRNA-mediated deletion of FASN significantly decreases the incorporation of ^13^C sodium acetate into palmitate but does not affect the total palmitate level [6]. Similarly, the relative independence from FASN activity to maintain stable intracellular lipid levels was observed in FASN^lox/lox^-PyMT and FASN^∆/∆^-PyMT MEFs [18]. In this study, we noted that heterozygous deletion of FASN in intestinal epithelial cells decreased the levels of several diglycerides in adenomas. However, we did not observe any unequivocal changes in the total levels of free fatty acids or sphingolipids in adenomas from mice with heterozygous deletion of FASN. We did not perform the comprehensive lipid analysis on adenomas from FASN^∆/∆^/Apc/Cre mice due to the limited number of mice of this genotype. However, analysis of the triglyceride levels in intestinal tissue and plasma from these mice shows a similar, statistically non-significant decrease in triglycerides for FASN^+/∆^/Apc/Cre mice compared to Apc/Cre. These results have several potential explanations. We have previously shown that both shRNA-mediated and pharmacological inhibition of FASN led to increased FA uptake [9,21], suggesting that the cellular lipid pool may be replenished by dietary FAs. Alternatively, in the Villin-Cre mouse model we used, Cre recombinase is expressed in villus and crypt epithelial cells of the small and large intestines, but the expression of FASN is intact in other cell types within intestinal tissues and in other organs. FASN is highly expressed in liver and adipose tissues [1]. It has been shown that the contribution of liver fatty acid synthesis appears to be less than that of fats derived from peripheral tissues or dietary fat [46] but FAs from all these sources can contribute to the total level of circulating lipids. The components of the tumor microenvironment and the intestinal microbiota can also alter the levels of lipid and metabolites [47]. FASN is highly expressed in endothelial cells [48,49], immune cells [50,51], and fibroblasts [52] associated with cancer, suggesting the potential impact of FASN expression in these cells on the levels of FAs observed during adenoma formation. To better understand the effect of FASN on lipid synthesis and lipid uptake and utilization, we plan to perform stable-isotope tracing studies to identify lipid species that drive Apc/FASN-driven carcinogenesis in a better-controlled environment such as organoid culture and primary CRC cell lines.

In current studies, the metabolic analysis of adenomas demonstrates changes in the level of several metabolites in glycolysis and the TCA cycle, including a decrease in D-glucose, pyruvate, citrate, malate, and fumarate. The changes in abundance of some metabolites did not reach statistical significance which can be explained by tissue- and cell-specific deletion of FASN in our model and the potential contribution of stromal compartment and microbiota to their levels. Based on PLS-DA analysis, the highest VIP was assigned to citrate, suggesting that a reduction in the level of citrate is the most significant change due to the reduced expression of FASN in our transgenic mouse models. This conclusion is supported by previously published work showing that the lack of FASN impairs glycolysis and the anaplerotic shift of the TCA cycle. This study also shows a diminished incorporation of carbon derived from glucose into the TCA cycle intermediates including citrate [18]. Citrate is an intermediate in the TCA cycle, which is produced in mitochondria by the action of CS, which combines acetyl-CoA and oxaloacetate to generate citrate for the TCA cycle [53]. Since CS catalyzes the first reaction of the TCA cycle, it is generally assumed to be the rate-limiting enzyme of the cycle [54]. Using RPPA and western blot analysis, we show that the level of CS is significantly increased in FASN^+/∆^/Apc/Cre and FASN^∆/∆^/Apc/Cre mice as compared to Apc/Cre, potentially, due to a decrease in the substrate availability. Several mechanisms contribute to mitochondrial citrate synthesis, including the serine/glycine pathway, truncated or reversed TCA cycle, and lactate uptake [55]. Citrate is important for ATP production, lipid synthesis, and epigenetic regulation [56]. Moreover, cytosolic citrate is obligatory for the promotion of cancer cell growth and proliferation [55], thus supporting our results that a decrease in FASN expression and the level of citrate are associated with less proliferative properties of intestinal adenomas and an increase of survival of mice. In addition, citrate is a key regulatory molecule, which targets (directly or indirectly) catabolic and anabolic pathways in a manner such that when one pathway is activated, the other is inhibited [53]. Indeed, administration of high doses of citrate inhibits the proliferation of various cancer cells via inhibition of glycolysis and other anti-cancer effects [53,57]. The complexity of citrate synthesis and utilization in cancer cells warrants the use of stable isotope tracing to better understand the metabolic adaptations associated with the downregulation of FASN expression in transgenic mouse models.

Consistent with the published study on CRC [58], our data show that CS is overexpressed in CRC as compared to normal mucosa. Our data shows that the heterozygous deletion of *Apc* in normal intestinal epithelium leads to the upregulation of both CS and FASN, suggesting that an increase in citrate and lipid synthesis are metabolic futures required for Apc-driven carcinogenesis. Even though we did not see significant changes in CS mRNA expression between normal mucosa and tumor tissues, the TCGA data demonstrate a significant correlation between the expression of CS and FASN in human colorectal cancer. We further confirmed this correlation by analyzing FASN and CS protein levels in matched normal colon and tumor tissues. Our data warrant in-depth studies to further delineate the mechanisms of how the level of citrate is regulated by FASN and better understand the functional consequences of these changes in CRC.

Another significant change identified due to a downregulation in FASN expression is a decrease in the beta-alanine level. Interestingly, beta-alanine was found to be the most upregulated metabolite in colon carcinoma tissues as compared to normal mucosa [59], suggesting it is potentially important for metabolic alterations in CRC. The synthesis of alanine from pyruvate is thought to lie in the mitochondria matrix [60]. Even though the role of alanine in cancer is poorly understood, emerging evidence suggests that alanine plays a role in the proliferation and survival of cancer cells [61]. Interestingly, alanine contributes significantly to bioenergetic and anabolic pathways including de novo synthesis of fatty acids in pancreatic cancer [62].

Limitations of the study. The rigorous analysis did not identify any statistically significant changes in free fatty acids, sphingolipids, and triglycerides between Apc/Cre mice and mice with altered expression of FASN. Moreover, even though the abundance of several metabolites decreased, statistical significance was not reached on all of them. There are several explanations for these results. We did not address the potential contribution of diet, adipose tissue, or stromal compartment (where the expression of FASN is intact in our mouse model) to the level of FAs or the contribution of metabolites to adenoma tissues and circulation [47]. Furthermore, we could not account for the effect of high heterogeneity of collected tissues and individual diversity among mice in our model, and this could greatly contribute to the outcome of our study. Indeed, it has been shown that principal component analysis does not show an unequivocal separation between cancer tissue and normal mucosa in CRC patients; paired comparison of cancer tissue and normal mucosa obtained from the same subject must be done to identify the difference [63]. Another potential explanation is that, in many cases, the patterns of non-significant differences in gene/protein expression or in the levels of metabolic intermediates as identified in the current study can lead to significant differences in the development of disease and, therefore, analysis of these patterns is as important as the identification of significant differences. Therefore, follow-up studies using different models need to be performed to confirm our findings and further delineate the effect of FASN in the development of CRC.

Use of stable isotope tracers in a controlled environment such as organoid cultures would address some issues described above and help advance understanding of mechanisms of how FASN contributes to carcinogenesis in CRC. ^13^C glucose and ^13^C acetate stable isotope tracing would facilitate a better understanding of the mechanisms of how changes in FASN expression alter glucose utilization and contribute to TCA cycle intermediates and lipid metabolism.

Our previous study demonstrates that pharmacological inhibition of FASN leads to a significant decrease in the levels of lipid species in several patient-derived xenografts treated with TVB-3664, and significant changes seem to be mostly associated with patient-derived xenografts established from metastatic tissues [9]. These data suggest that mutations other than in the *Apc* gene, stage of cancer, and aggressiveness of the tumor can contribute to diversity in lipid utilization and uptake. Therefore, analysis of other models beyond the Apc-driven model should be utilized to better understand the role of de novo lipid synthesis in CRC carcinogenesis.

In summary, despite some limitations, our study provides compelling evidence that FASN plays an important role in CRC initiation by promoting the expression of genes supporting cellular proliferation and upregulating metabolic pathways involved in catabolic reactions and energy production. Therefore, this work warrants further investigation of FASN as a potential target for CRC prevention in the setting of more complex models when CRC is driven by mutations other than the *Apc* gene to further confirm the potential for use of FASN-targeted therapy in individuals who have a high risk based on either genetics or screening colonoscopy results.

## 4. Materials and Methods

### 4.1. Mouse Colonies

Mice were housed at the facility supervised by the Division of Laboratory Animal Resources, University of Kentucky in accordance with the NIH Guide for the Care and Use of Lab Animals (https://www.ncbi.nlm.nih.gov/books/NBK54050/, accessed on 31 July 2021). All animal experimental procedures were carried out under approval from the University Committee on Use and Care of Animals, University of Kentucky, protocol # 2016-2521. Mice were fed 2018 Teklad global 18% protein rodent diets from ENVIGO during breeding, strain maintenance, and experimental procedures. C57BL/6J mice with LoxP-flanked FASN alleles (FASN^f/f^) were obtained from Clay Semenkovich, MD, at Washington University [23]. Apc/Villin-Cre mouse colonies with hetero- and homozygous deletion of FASN were established by mating these mice with Villin-Cre mice (B6.Cg-Tg(Vil1-cre)1000 Gum/J, stock #021504) and with Apc mice (C57BL/6-Apc^tm1Tyj^/J, stock #00945).

### 4.2. Survival Analysis and Tumor Number Studies

For survival studies, mice were observed daily for signs associated with adenoma development such as weight loss, lethargy/cachexia, paleness of the paws, hinged posture, obstruction, bloody stool, and anal bleeding. Animals were euthanized when they reached the endpoint (recumbent and unable to drink and eat due to symptoms associated with disease progression). For survival studies, we observed 26 Apc/Cre male mice, 22 Apc/Cre females, 21 FASN^+/∆^/Apc/Cre males, and 27 FASN^+/∆^/Apc/Cre females.

For adenoma count, intestines were removed. The section of 10 cm starting 1 cm from cecum was used for determination of adenoma number. The intestine was washed with PBS, placed on an ice-cold metal platform, opened, cleaned, and the number of visual adenomas was counted. The tumor numbers are reported as the average number of tumors for male and female mice. Tissues from 8 Apc/Cre male mice, 12 Apc/Cre females, 12 FASN^+/∆^/Apc/Cre males, and 11 FASN^+/∆^/Apc/Cre females were used for analysis.

The tissues were processed for immunohistochemistry analysis using the swiss-roll techniques or adenomas were collected for further quantitative Reverse Transcription Polymerase Chain Reaction and western blot analysis.

### 4.3. Histologic Analysis and Immunohistochemical (IHC) Staining

Paraffin-embedded tissue section slides were prepared from transgenic mouse tissues using the Biospecimen Procurement and Translational Pathology Shared Resource Facility services. Tissue slides were stained with hematoxylin and eosin. For IHC staining, paraffin-embedded tissue sections were deparaffinized, rehydrated, and antigen retrieval was performed using Antigen Retriever Buffer #T6455 (Sigma-Aldrich Inc., St. Louis, MO, USA). IHC staining was performed using ImmPRESS^®^ HRP Universal (Horse Anti-Mouse/Rabbit IgG) PLUS Polymer Kit, Peroxidase, MP-7800 (Vector Laboratories Inc., Burlingame, CA, USA) according to manufacturer instructions. The stained sections were visualized and imaged using a Nikon Eclipse 80i upright microscope (Melville, NY, USA). The HALO digital pathology analysis platform at the Biospecimen Procurement and Translational Pathology Shared Resource Facility was used to quantify the percentage of Ki67 positive cells and intensity of staining in intestinal tissues.

### 4.4. Western Blot Analysis

Mouse adenoma tissues were harvested and homogenized using metal beads in Cell Lysis Buffer #9803 (Cell Signaling, Danvers, MA, USA) supplemented with additional protease inhibitors. Equal amounts of cell lysates were resolved by SDS-PAGE and subjected to western blot analysis.

### 4.5. Antibodies for Western Blot and IHC Staining

Antibodies were purchased from Cell Signaling (Danvers, MA): Fatty Acid Synthase (C20G5) Rabbit mAb (#3180), Citrate Synthase (D7V8B) Rabbit mAb (#14309), Ki-67 (D3B5) Rabbit mAb (Mouse Preferred; IHC Formulated) #12202. All antibodies were used at a concentration of 1∶1000 for western blot and 1:100 for IHC.

### 4.6. RNA-Sequencing and Gene Set Enrichment Analysis

RNA samples from pulled adenomas (*n* = 3) collected from Apc/Villin-Cre, FASN^+/∆^/Apc/Villin-Cre and FASN^∆^^/∆^/Apc/Villin-Cre were prepared using a QIAGEN RNeasy kit and library preparation, sequencing, and standard bioinformatics analysis were performed by BGI Genomics, Cambridge, MA, USA (https://www.bgi.com/global/, accessed on 31 July 2021). The quality control assessment of samples and detailed summary of the sequencing coverage, quality statistics, and data analysis report are included in Supplemental Table S1A,B. The gene set enrichment analysis was performed by the Biostatistics and Bioinformatics Shared Resource Facility, University of Kentucky (Lexington, KY, USA) using gene set enrichment analysis (GSEA) software (version 4.0.3) and the KEGG pathways in the Molecular Signature Database (MSigDB) [64,65]. Hypergeometric tests were used to test enrichment of KEGG pathways based on the R package clusterProfiler (version 3.18.1) [66].

### 4.7. Metabolite Extraction

For metabolic analysis, the adenomas were removed from intestine, rinse with ice cold PBS and immediately cryopreserved to minimize any further changes in metabolite levels. Isolated adenomas were removed from cryostorage and transferred to a micro vial set for use with a Freezer/Mill Cryogenic Grinder (SPEX SamplePrep model 6875D, Cole-Parmer North America, Vernon Hills, IL, USA). Tissue was pulverized to 5 μm particles. Metabolites were extracted directly from the micro vial by the addition of 1 mL of 50% methanol containing 20 mL-norvaline (procedural, internal control) and separated into polar (aqueous layer) and insoluble pellet (protein/DNA/RNA/glycogen) by centrifugation at 4 °C, 15,000 rpm for 10 min. The pellet was subsequently washed four times with 50% methanol and once with 100% methanol. The pellet was then hydrolyzed in 200 μL of 3N hydrochloric acid and then 200 μL of 100% methanol was added before drying. The polar and pellet fraction was dried at 10^−3^ mBar using a SpeedVac (ThermoFisher Scientific, Waltham, MA, USA) followed by derivatization. The insoluble pellet was hydrolyzed as described [67].

### 4.8. Sample Derivatization and Gas Chromatography-Mass Spectrometry (GC-MS) Quantification

Dried polar and insoluble samples were derivatized by the addition of 50 μL of 20 mg/mL methoxyamine hydrochloride in pyridine, vortexed thoroughly, and incubated for 1.5 h at 30 °C. Sequential addition of 80 μL of N-methyl-trimethylsilyl-trifluoroacetamide followed with an incubation time of 30 min at 37 °C with thorough vortexing between addition of solvents. The mixture was then transferred to an amber, v-shaped glass chromatography vial and analyzed by GC-MS.

An Agilent 7800B gas-chromatography coupled to a 5977B mass spectrometry detector was used for this study (Agilent, Santa Clara, CA, USA). GC-MS protocols were similar to those described previously [68,69] except a modified temperature gradient was used for GC: Initial temperature was 130 °C, held for 4 min, rising at 6 °C/min to 243 °C, rising at 60 °C/min to 280 °C, held for 2 min. The electron ionization energy was set to 70 eV. Scan (*m*/*z*: 50–800) and full scan mode were used for metabolomics analysis. Mass spectra were translated to relative metabolite abundance using MassHunter MS quantitative software matched to the FiehnLib metabolomics library (available through Agilent) for retention time and fragmentation pattern [69,70,71]. Relative abundance was corrected for recovery using the L-norvaline standard and adjusted to protein input represented by the sum of amino acids from the pellet fraction also analyzed by GC-MS.

### 4.9. Metabolite Analysis

Data were uploaded into MetaboAnalyst version 5.0 (Xia Lab, McGill University, Montreal, QC, Canada) for partial least-squares discriminant analysis (PLS-DA), variable importance in projection (VIP) analysis, and clustering heat map analysis. Data was uploaded as a CSV file and auto-scaled (mean-centered and divided by the standard deviation of each variable). The VIP score of a metabolite is calculated as a weighted sum of the squared correlations between the PLS-DA components. Heatmaps were organized using all metabolic features and distance measured by a Euclidean analysis.

### 4.10. Lipidomic Analysis

Lipid analysis of mouse adenoma tissues was performed by Lipidomics Shared Resources (Analytical Unit) at the Medical University of South Carolina according to their standard protocols (https://hollingscancercenter.musc.edu/research/shared-resources/lipidomics, accessed on 31 July 2021). The data for free fatty acids, diglycerides, and sphingolipids were processed and analyzed by Markey Cancer Center Biostatistics and Bioinformatics Shared Resource Facility based on the following procedure. Below the detection limit measurements of a metabolite were imputed by the minimum of detected values of the metabolite across samples divided by square root of 2. Data were then log2-transformed and used as input for the limma package to compare metabolic profile between Apc/Cre and FASN^+/∆^/Apc/Cre groups [72]. *p* values and fold changes were calculated based on the moderated t-statistics. Multiple comparisons adjustment was performed by controlling the false discovery rate (FDR) based on the Benjamini and Hochberg method. An FDR < 0.05 was considered statistically significant.

### 4.11. Reverse Phase Protein Analysis

RPPA was performed on protein lyates of adenomas from Apc/Villin-Cre, FASN^+/∆^/Apc/Villin-Cre and FASN^∆^^/∆^/Apc/Villin-Cre mice by the Center for Environmental and Systems Biochemistry (Redox Metabolism Shared Resource Facility, University of Kentucky) as previously described [28]. The raw RPPA data obtained in 6 dilution steps were processed based on the following procedure. First, data quality control was performed by plotting the relationship between background-corrected intensity and dilution step. Proteins with low measurement quality as reflected by large variation across replicates or unreliable curve trend were excluded. Secondly, nonlinear curve fitting for the background corrected intensity vs. dilution step was applied based on the “serial dilution curve” algorithm [73] to infer the concentration of each protein in the original undiluted sample. Due to the low protein concentration measurement in the 6th dilution step, the nonlinear curve fitting was only based on data from the first 5 dilution steps. Third, protein concentrations were log2-transformed and normalized based on the median normalization method described in https://www.tcpaportal.org/tcpa/faq.html (accessed on 13 December 2020) and [74]. Finally, the Wilcoxon Rank Sum test was used for differential expression analysis comparing experimental groups. Multiple comparisons adjustment was performed by the Benjamini and Hochberg method. Differentially expressed proteins were identified by false discovery rate < 0.05. Heatmap and volcano plots were generated to demonstrate the relative expression changes of proteins of interest.

### 4.12. Analysis of Correlation between FASN and Citrate Synthase (CS)

Correlations between FASN and CS were determined based on RNA-Seq data of CRC patient tumor tissues from The Cancer Genome Atlas [75]. The RNA-Seq data (FPKM values) were downloaded from the Genomic Data Commons (https://www.cancer.gov/tcga, accessed on 4 August 2021) and converted to TPM values. Spearman’s rank correlation coefficient was used to quantify the correlation between FASN and CS expressions.

## Data Availability

The raw data supporting the conclusions of this article will be made available by the authors, without undue reservation. The datasets presented in this study can be found in supplemental materials and freely available in Dryad repository.

## Supplementary Materials

The following supporting information can be downloaded at: https://www.mdpi.com/article/10.3390/ijms23126510/s1.
