# Supplementary material for: Tissue-Specific Downregulation of Fatty Acid Synthase Suppresses Intestinal Adenoma Formation via Coordinated Reprograming of Transcriptome and Metabolism in the Mouse Model of Apc-Driven Colorectal Cancer
Source: Int J Mol Sci. 2022 Jun 10;23(12):6510. doi: 10.3390/ijms23126510 (PMC9245602; doi:10.3390/ijms23126510)
Supplement: Supplementary file 1 [file ijms-23-06510-s001.zip › ijms-1768628-supplementary-1/Supplementary/FigureS1.pdf]

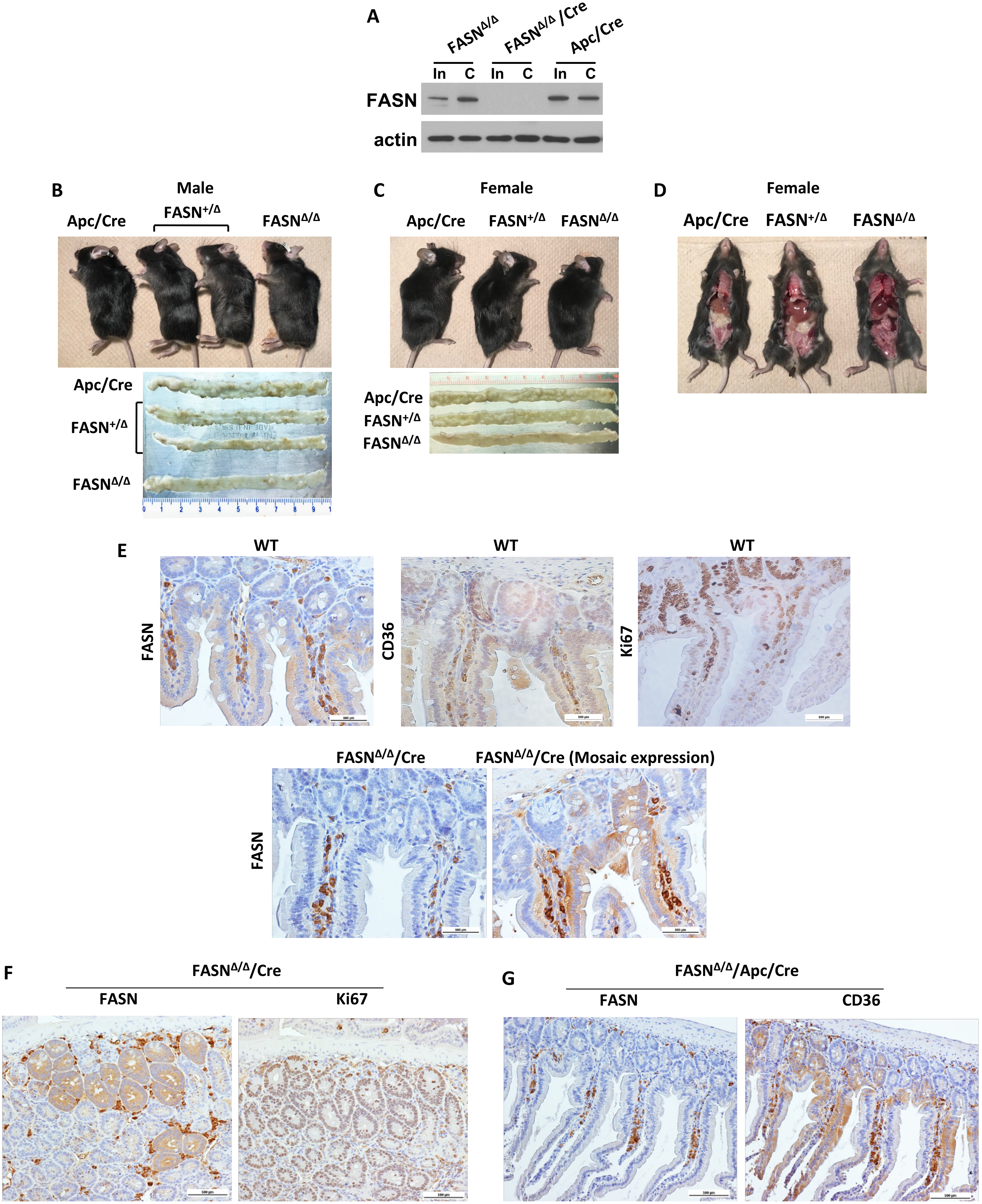

Figure S1. Characterization of transgenic mouse models. **(A)** Western blot analysis confirming a complete knockdown of FASN in FASN$^{\Delta\Delta}$/Cre mice. **(B-C)** Representative images of male (B) and female (C) mice showing no significant differences in phenotype based on FASN deletion. **(D)** Differences in visceral fat accumulation among female mice with different genotypes. **(E)** FASN expression in normal mucosa in wild type mice and in mice with homozygous deletion of FASN; mosaic expression of FASN is shown on the right. **(F)** Mosaic expression in normal mucosa and corresponding Ki67 staining. **(G)** CD36 staining in Apc/Cre mice with homozygous deletion of FASN.
