# Supplementary material for: Tissue-Specific Downregulation of Fatty Acid Synthase Suppresses Intestinal Adenoma Formation via Coordinated Reprograming of Transcriptome and Metabolism in the Mouse Model of Apc-Driven Colorectal Cancer
Source: Int J Mol Sci. 2022 Jun 10;23(12):6510. doi: 10.3390/ijms23126510 (PMC9245602; doi:10.3390/ijms23126510)
Supplement: Supplementary file 1 [file ijms-23-06510-s001.zip › ijms-1768628-supplementary-1/Supplementary/FigureS3.pdf]

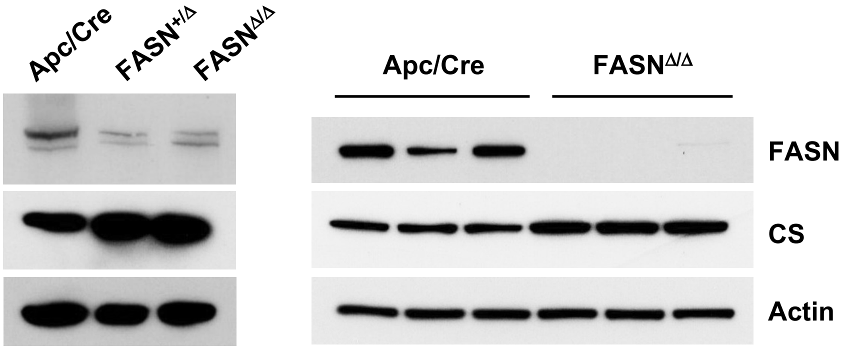


**Figure S3.** Heterozygous and homozygous deletion of FASN in Apc/Cre mice is associated with an increase in citrate synthase expression.
