# Supplementary material for: Tissue-Specific Downregulation of Fatty Acid Synthase Suppresses Intestinal Adenoma Formation via Coordinated Reprograming of Transcriptome and Metabolism in the Mouse Model of Apc-Driven Colorectal Cancer
Source: Int J Mol Sci. 2022 Jun 10;23(12):6510. doi: 10.3390/ijms23126510 (PMC9245602; doi:10.3390/ijms23126510)
Supplement: Supplementary file 1 [file ijms-23-06510-s001.zip › ijms-1768628-supplementary-1/Supplementary/Table S1B.pdf]

Table S1B: Complete report for RNA-Seq analysis

HUMqxoE [Transcriptome Resequencing Report]

2020/12/15

@2020 BGI All Rights Reserved

© 2019 BGI All Rights Reserved.

Table of Contents

Results 4
- 1 Abstract 4
- 2 Sequencing data Filtering 4
- 3 Genome Mapping 5
- 4 Novel Transcripts Prediction 6
- 5 SNP and INDEL Detection 7
- 6 Differentially Splicing Gene Detection 9
- 7 Gene Expression Analysis 10
- 8 Circos Diagram 16
- 9 Gene expression cluster analysis 17
- 10 Time Series Analysis 18
- 11 Differentially Expressed Gene Detection 20
- 12 Venn Diagram of DEG 23
- 13 Clustering Analysis of DEG 23
- 14 Gene Ontology Analysis of DEG 24
- 15 Pathway Analysis of DEG 27
- 16 Transcription Factor Prediction of DEG 29
- 17 Protein-Protein Interaction Networks of DEG 31

Methods 32
- 1 Experiment and Bioinformatics Workflow 32
- 2 Sequencing Reads Filtering 34
- 3 Genome Mapping 34
- 4 Novel Transcript Prediction 35
- 5 SNP and INDEL Detection 36
- 6 Differentially Splicing Gene Detection 37
- 7 Gene Expression Analysis 38
- 8 Circos Diagram 38
- 9 Gene expression cluster analysis 39
- 10 Time Series Analysis 39
- 11 DEG Detection 39
- 12 Hierarchical Clustering Analysis of DEG 39
- 13 Gene Ontology Analysis of DEG 39
- 14 Pathway Analysis of DEG 40
- 15 Transcription Factor Prediction of DEG 40
- 16 PPI Analysis of DEG 41

Help 41
- 1 FASTQ Format 41

© 2019 BGI All Rights Reserved.

2 Relationship between sequencing error rate and sequencing quality value 41
3 VCF format 42
4 Differentially Splicing Gene format 42
5 How to view the cluster analysis report 43
6 DEG list format 43
7 Cluster list format 44
8 How to read DEG GO enrichment analysis result 44
9 How to read DEG pathway enrichment analysis result 44
10 TF 46
FAQs 47
References 47

© 2019 BGI All Rights Reserved.

Results

1 Abstract

In this project, we sequence 3 samples used DNBseq platform, averagely generating about 4.34 Gb bases per sample. The average mapping ratio with reference genome is 94.51%, the average mapping ratio with gene is 75.68%; 16,948 genes were identified in which 16,948 of them are known genes and 707 of them are novel genes. 6,515 novel transcipts were identified in which 4,805 of them are previously unknown splicing event for known genes, 707 of them are novel coding transcripts without any known features, and the remaining 1,003 are long noncoding RNA.

2 Sequencing data Filtering

Firstly,we remove the reads mapped to rRNA and get raw data. Then the sequencing reads which containing low-quality, adaptor-polluted and high content of unknown base (N) reads, should be processed to be removed before downstream analyses. The filter composition statistics of raw data is shown in **Figure S1**. Clean reads quality metrics are shown as **Table S1**. The distribution of base quality is shown as **Figure S2**.

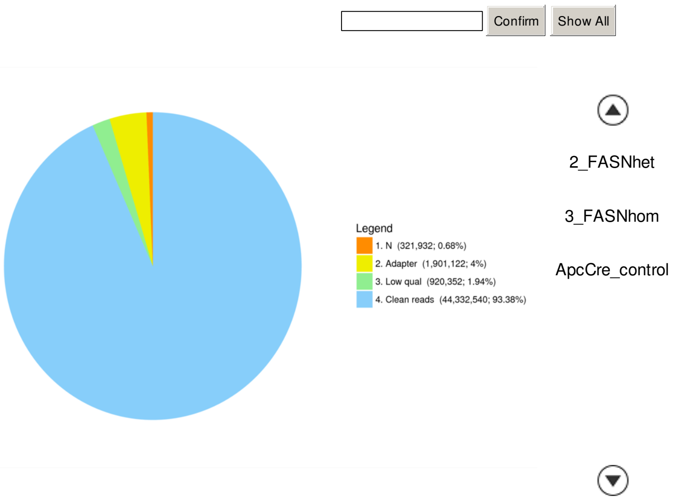

Figure S1 Filter composition of raw data .

N: The total amount of reads which contain more than 5% unknown N base; Adaptor: The total amount of reads which contain adaptors; Low quality: More than 20% of bases in the total read have quality score lower than 15; Clean reads: Reads filtered with N reads, reads have adaptors and low quality reads.

Table S1 Clean reads quality metrics (Download)

Sample	Total Raw Reads(M)	Total Clean Reads(M)	Total Clean Bases(Gb)	Clean Reads Q20(%)	Clean Reads Q30(%)	Clean Reads Ratio(%)	rRNA Ratio(%)
2_FASNhet	47.48	44.33	4.43	96.74	88.61	93.38	0.22
3_FASNhom	44.98	42.27	4.23	96.97	89.23	93.98	0.72
ApcCre_control	47.48	43.55	4.36	97.61	90.21	91.74	0.28

Samples: Sample names
Total Raw Reads(Mb): The reads(removed rRNAs) amount before filtering, Unit: Mb
Total Clean Reads(Mb): The reads amount after filtering, Unit: Mb
Total Clean Bases(Gb): The total base amount after filtering, Unit: Gb
Clean Reads Q20(%): The Q20 value for the clean reads
Clean Reads Q30(%): The Q30 value for the clean reads

© 2019 BGI All Rights Reserved.

Clean Reads Ratio(%): The ratio of the amount of clean reads
rRNA Ratio(%): The ratio of rRNA

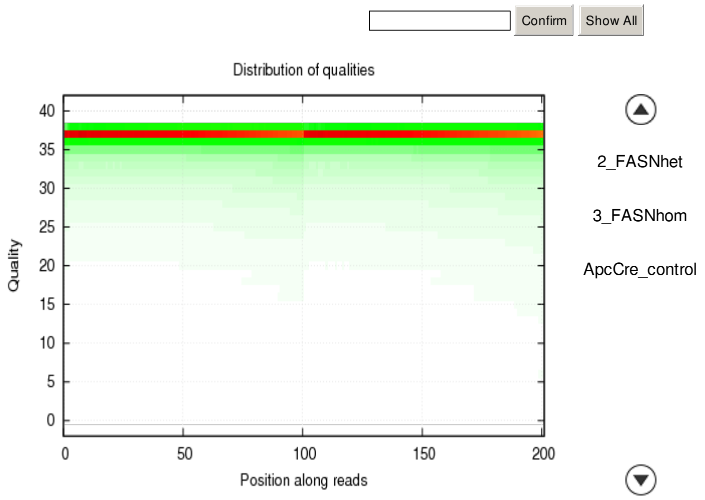

Figure S2 Distribution of base quality on clean reads.

X axis represents base positions along reads. Y axis represents base quality value. Each dot in the image represents the number of total bases with certain quality value of the corresponding base along reads. Darker dot color means greater base number. If the proportion of the bases with low quality (< 20) is very low, that means the sequencing quality of this lane is good.

3 Genome Mapping

After reads filtering, we map clean reads to reference genome using HISAT2[2]. On average 94.51% reads are mapped, and the uniformity of the mapping result for each sample suggests that the samples are comparable. The mapping details are shown as **Table S2**.

Table S2 Summary of Genome Mapping (Download)

Sample	Total CleanReads	Total MappingRatio	Uniquely MappingRatio
2_FASNhet	44,332,540	93.75%	68.44%
3_FASNhom	42,269,598	94.21%	67.22%
ApcCre_control	43,552,282	95.57%	72.19%

Sample: Sample name
Total CleanReads: The amount of clean reads
Total MappingRatio: The percentage of mapped reads
Uniquely MappingRatio: The percentage of reads that map to only one location of reference

At the same time, we provide the bam files for the genome mapping result. IGV(Integrative Genomics Viewer) tool can be used to review the mapping result. IGV supports importing multiple samples for comparison and show the distribution of reads in the exon, intron, UTR, intergenic areas based on the annotation result. **Figure S3** is an example of IGV display. In addition, **Figure S4** shows an example of sashimi-plot, which can plot reads densities along exons and junctions for multiple samples. IGV Genomic Data Browsing Method please refer to the directory IGV/IGV_readme.pdf.

© 2019 BGI All Rights Reserved.

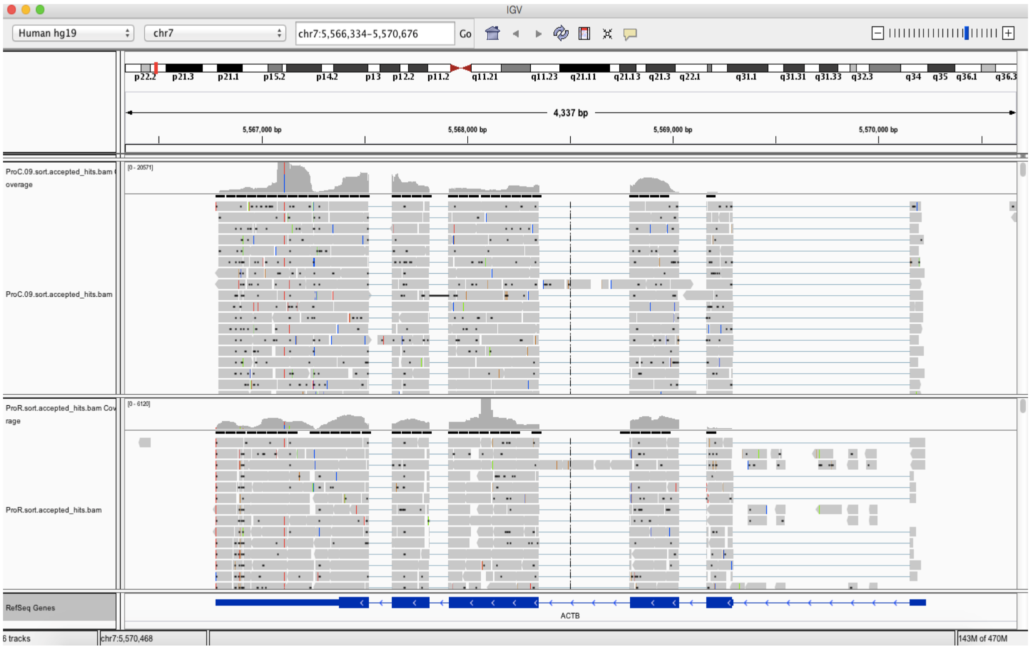

Figure S3 IGV comparison results.

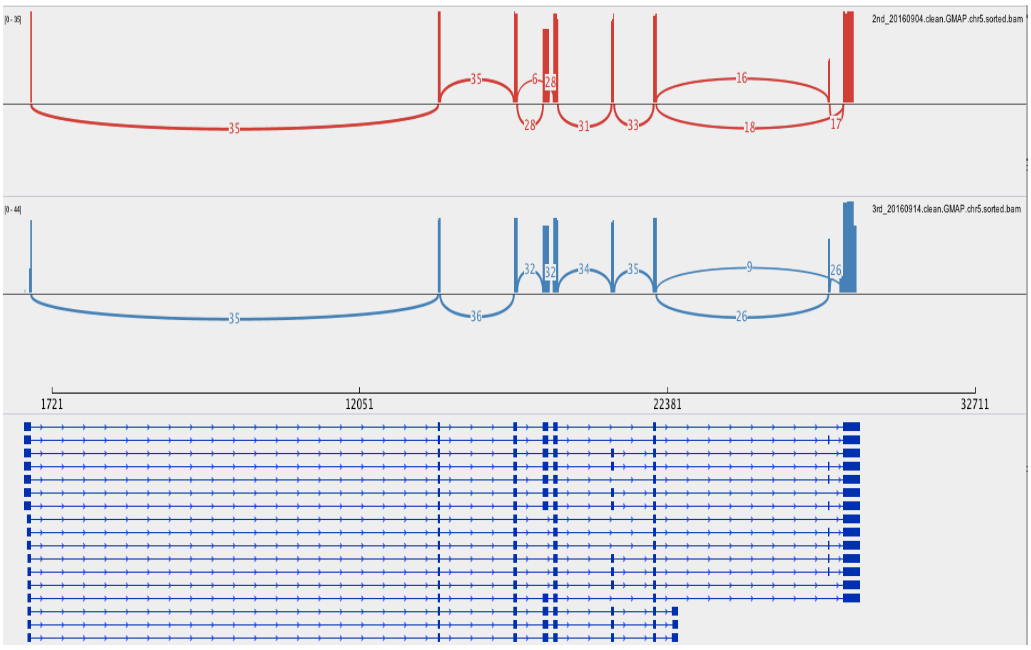

Figure S4 Sashimi plots to screen differentially spliced exons along genomic regions.

4 Novel Transcripts Prediction

After genome mapping, we use StringTie[3] to reconstruct transcripts, and with genome annotation information we identify novel transcripts by using Cuffcompare(a tool of Cufflinks [4]) and predict the coding ability of those new transcripts using CPC [5]. In total, we identify 6,515 novel transcripts, the detailed information is shown as **Table S3**.

Table S3 Summary of Novel Transcripts. (Download)

Total_Novel_Transcript	Coding_Transcript	Noncoding_Transcript	NovelIsoform	NovelGene

© 2019 BGI All Rights Reserved.

6,515	5,512	1,003	4,805	707

Total_Novel_Transcript: The amount of predicted novel transcripts
Coding_Transcript: The amount of predicted coding transcripts
Noncoding_Transcript: The amount of predicted noncoding transcripts
NovelIsoform: The amount of predicted coding transcripts that previously unknown splicing event for a known gene
NovelGene: The amount of predicted coding transcripts that previously unknown

5 SNP and INDEL Detection

After genome mapping, we use GATK[7] to call ***SNP*** and ***INDEL*** variant for each sample. Final results are stored in VCF format. The ***SNP*** summary is shown as **Table S4**, and **Figure S5**. We also generate a friendly-interfaced ***SNP*** summary in EXCEL format shown as **Table S13**. Then, we statistic the location of ***SNP*** and ***INDEL*** , shown as **Figure S6** and **Figure S7**. **Table S4** SNP variant type summary. (Download)

Sample	A-G	C-T	Transition	A-C	A-T	C-G	G-T	Transversion	Total
2_FASNhet	4,638	4,683	9,321	899	971	879	936	3,685	13,006
3_FASNhom	3,674	3,690	7,364	734	836	683	724	2,977	10,341
ApcCre_control	5,033	5,072	10,105	999	978	967	938	3,882	13,987

Sample: Sample name
A-G: The amount of A-G variant type
C-T: The amount of C-T variant type
Transition: The amount of A-G and C-T variant type
A-C: The amount of A-C variant type
A-T: The amount of A-T variant type
C-G: The amount of C-G variant type
G-T: The amount of G-T variant type
Transversion: The amount of A-C, A-T, C-G and G-T variant type
Total: The amount of all variant type

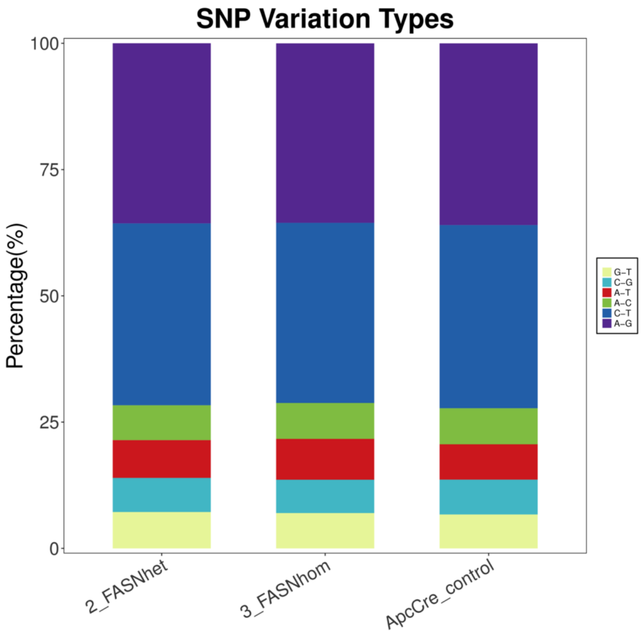

Figure S5 SNP variant type distribution.

X axis represents the type of SNP. Y axis represents the number of SNP.

© 2019 BGI All Rights Reserved.

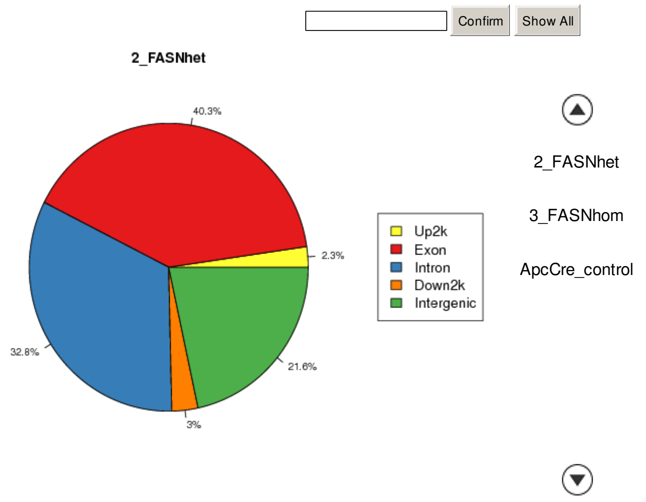

Figure S6 Distribution of SNP location.

Up2k means upstream 2,000 bp area of a gene. Down2k means downstream 2,000 bp area of a gene.

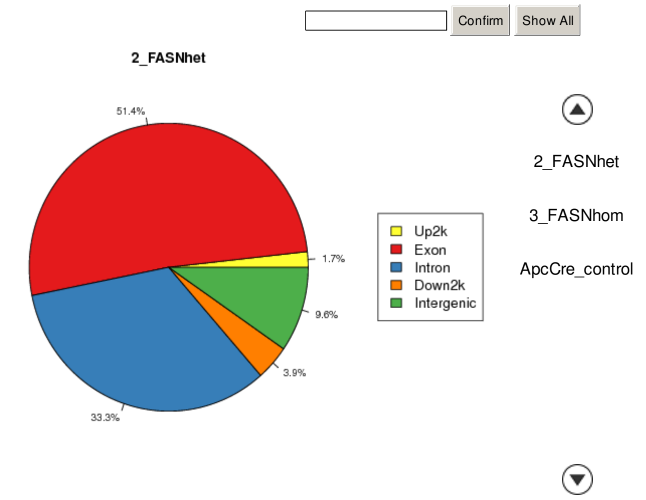

Figure S7 Distribution of INDEL location.

Up2k means upstream 2,000 bp area of a gene. Down2k means downstream 2,000 bp area of a gene.

The VCF format ***SNP*** and ***INDEL*** result of each sample are shown as tables below(see VCF format in help page **VCF format**).

Table S5 SNP list of 2_FASNhet: (Download)

Table S6 SNP list of 3_FASNhom: (Download)

Table S7 SNP list of ApcCre_control: (Download)

Table S8 Summary of SNP result in VCF format: (Download)

Table S9 INDEL list of 2_FASNhet: (Download)

Table S10 INDEL list of 3_FASNhom: (Download)

Table S11 INDEL list of ApcCre_control: (Download)

Table S12 Summary of INDEL results in VCF format: (Download)

© 2019 BGI All Rights Reserved.

Table S13 Summary of SNP results in excel: (Download)

6 Differentially Splicing Gene Detection

After genome mapping, we use rMATS [8] to detect differentially splicing gene (***DSG***) between samples. DSGs are regulated by alternative splicing (AS), which allows the production of a variety of different isofroms from one gene only. Changes in relative abundance of isoforms, regardless of the expression change, indicate a splicing-related mechanism. We detect five types of AS events, including Skipped Exon (SE), Alternative 5' Splicing Site (A5SS), Alternative 3' Splicing Site (A3SS), Mutually exclusive exons (MXE) and Retained Intron (RI). The ***Gene Ontology*** classification is shown as **FigureS8** and the summary of gene splicing is shown in **Figure S9**.

Confirm Show All

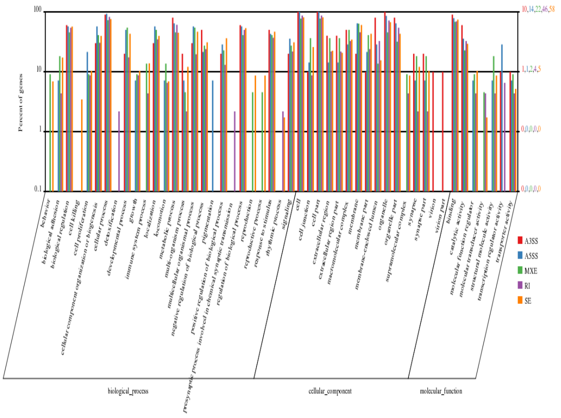

2_FASNhet-VS-3_FASNhom

ApcCre_control-VS-2_FASNhet

ApcCre_control-VS-3_FASNhom

Figure S8 Gene Ontology classification of DSGs.

X axis represents the Gene Ontoloty functions. Y axis represents the number of DSGs. Different columns represent different samples.

The results of each provided compare plan are shown as tables below (see ***DSG*** format in help page **Differentially Splicing Gene format**).

Table S14 A3SS regulated DSG list of 2_FASNhet-VS-3_FASNhom: (Download)

Table S15 A5SS regulated DSG list of 2_FASNhet-VS-3_FASNhom: (Download)

Table S16 MXE regulated DSG list of 2_FASNhet-VS-3_FASNhom: (Download)

Table S17 RI regulated DSG list of 2_FASNhet-VS-3_FASNhom: (Download)

Table S18 SE regulated DSG list of 2_FASNhet-VS-3_FASNhom: (Download)

Table S19 A3SS regulated DSG list of ApcCre_control-VS-2_FASNhet: (Download)

Table S20 A5SS regulated DSG list of ApcCre_control-VS-2_FASNhet: (Download)

Table S21 MXE regulated DSG list of ApcCre_control-VS-2_FASNhet: (Download)

Table S22 RI regulated DSG list of ApcCre_control-VS-2_FASNhet: (Download)

Table S23 SE regulated DSG list of ApcCre_control-VS-2_FASNhet: (Download)

Table S24 A3SS regulated DSG list of ApcCre_control-VS-3_FASNhom: (Download)

Table S25 A5SS regulated DSG list of ApcCre_control-VS-3_FASNhom: (Download)

© 2019 BGI All Rights Reserved.

Table S26 MXE regulated DSG list of ApcCre_control-VS-3_FASNhom: Download)

Table S27 RI regulated DSG list of ApcCre_control-VS-3_FASNhom: (Download)

Table S28 SE regulated DSG list of ApcCre_control-VS-3_FASNhom: (Download)

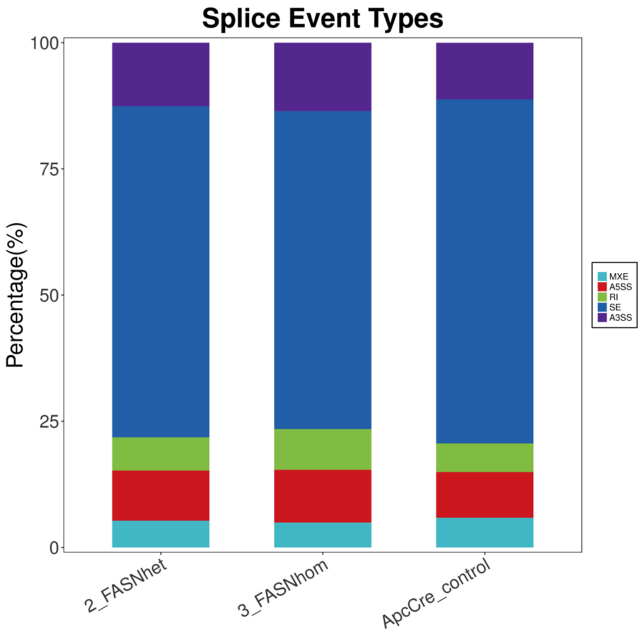

Figure S9 Statistic of Splicing.

X axis means the type of splicing. Y axis means the amount. Different columns represent different splicing events.

7 Gene Expression Analysis

7.1 Gene Mapping and Expression

After novel transcript detection, we merge novel coding transcripts with reference transcripts to get complete reference, then we map clean reads to it using Bowtie2\[9\], then calculate gene expression level for each sample with ***RSEM*** \[10\]. The gene mapping ratio is shown as **Table S29**. And the number of genes and transcripts of each sample is shown as **Table S30**.

Table S29 Summary of gene mapping ratio (Download)

Sample	Total CleanReads	Total MappingRatio	Uniquely MappingRatio
2_FASNhet	44,332,540	78.23	74.74
3_FASNhom	42,269,598	70.20	66.02
ApcCre_control	43,552,282	78.61	74.76

Sample: Sample name
Total CleanReads: The amount of Clean reads
Total MappingRatio: The percentage of mapped reads (%)
Uniquely MappingRatio: The percentage of uniquely mapped reads (%)

Table S30 Genes and Transcripts statistics Download)

Sample	Total GeneNumber	Total TranscriptNumber
2_FASNhet	16,287	23,252

© 2019 BGI All Rights Reserved.

3_FASNhom	14,662	19,924
ApcCre_control	16,218	23,117

Sample: Sample name
Total GeneNumber: The amount of all genes
Total TranscriptNumber: The amount of all transcripts

The expressed gene list summary is shown in **Table S34**.

Table S31 List of gene expression levels for sample 2_FASNhet : (Download)

Table S32 List of gene expression levels for sample 3_FASNhom : (Download)

Table S33 List of gene expression levels for sample ApcCre_control : (Download)

Table S34 The result of gene expression. (Download)

gene_id	transcript_id(s)	length	expected_count	FPKM	SymbolID
100009600	NM_001103168	1,010.00	11.00	0.68	Zglp1
100017	NM_145554	2,671.00	949.00	21.13	Ldlrap1
100019	NM_001081392	17,959.00	543.00	1.75	Mdn1
100033459	NM_001162938	2,987.00	5.00	0.10	Ifi208
100034251	NM_001081957	485.00	44.00	6.32	Wfdc17

gene_id: The gene ID
transcript id: The transcript ID
length: Gene length
expected count: The reads amount which mapped to the gene
FPKM: The gene FPKM
Symbol: Gene Symbol ID

7.2 Sequencing Saturation

Sequencing data saturation analysis is used to measure whether the depth of sequencing data is sufficient for bioinformatics analysis. With the number of sequenced reads increasing, the number of identified genes is also increased. However, when the number of sequenced reads reaches a certain amount, the growth curve of identified genes flattens, indicating that the number of identified genes tends to reach the saturation. **Figure S10**displays saturation analysis for each sample.

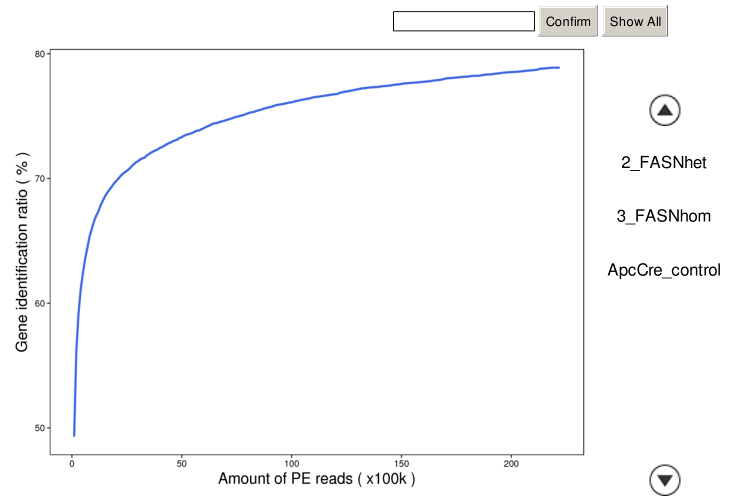

Figure S10 Sequencing Saturation .

The X axis represents the number of clean reads, in units of 100k; the Y axis indicates the number of detected genes, in units of %.

© 2019 BGI All Rights Reserved.

7.3 Reads Coverage and Distribution Analysis of Transcripts

We calculate the reads coverage and the reads distribution of each detected transcript, shown as **Figure S11** and **Figure S12**, respectively. For samples with good sample quality and sufficient sequencing data, most transcripts will be completely covered, and reads will be evenly distributed throughout the transcript.

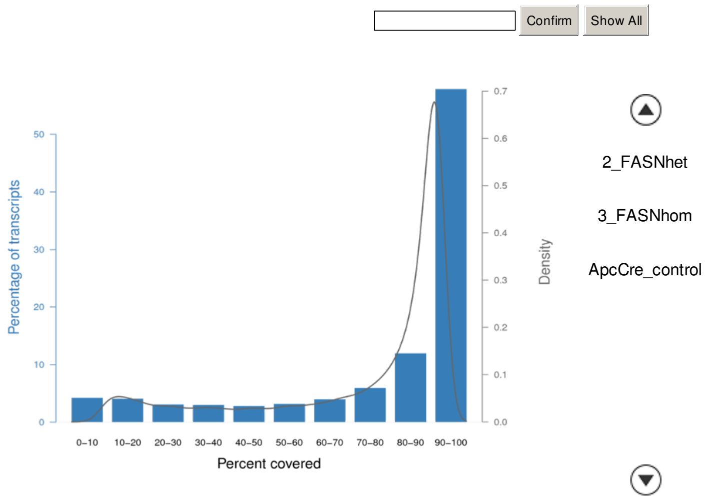

Figure S11 Reads coverage on transcripts.

X axis represents the reads coverage. Y axis on left side represents the percentage of transcripts. Y axis on right side represents the density of transcripts.

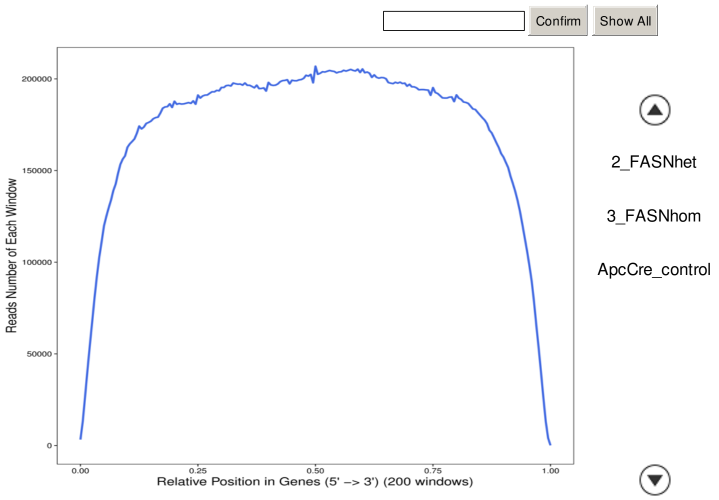

Figure S12 Reads distribution on transcripts.

X axis represents the position along transcripts. Y axis represents the number of reads.

7.4 Correlation between samples

In order to reflect the gene expression correlation between samples, we calculated the Pearson correlation coefficients for all gene expression levels between each two samples and reflected these coefficients in the form of heat maps, shown as **Figure S13**. All the samples were hierarchical clustering by the expression level of all genes, The results

© 2019 BGI All Rights Reserved.

are shown in **Figure S14**, which can directly reflect the relationship between each two samples.

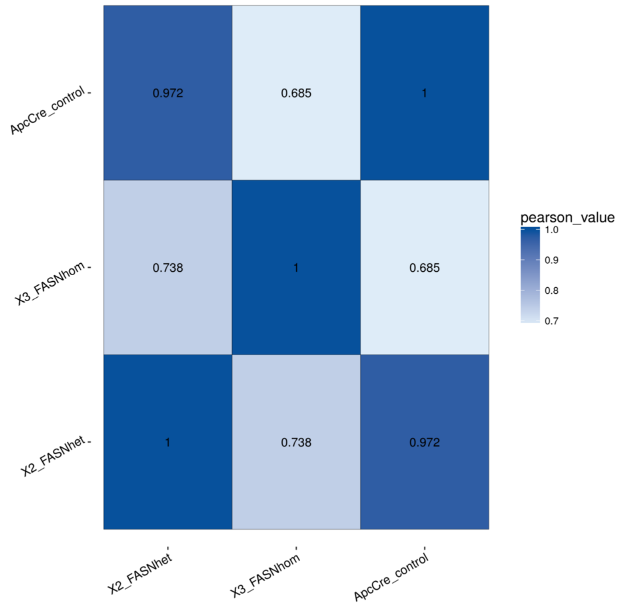

Figure S13 Correlation analysis between samples.

The X and Y axis represent each sample. The color represents the correlation coefficient (the darker the color,the higher the correlation, the lighter the color ,the lower the correlation).

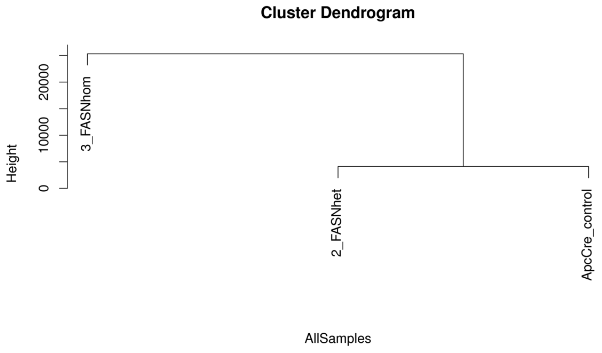

Figure S14 Analysis of hierarchical clustering between samples.

The closer the samples were to each other, the more similar the expression level was.

Principal component analysis (PCA) is a statistical procedure that uses an orthogonal

© 2019 BGI All Rights Reserved.

transformation to convert a set of observations of possibly correlated variables into a set of values of linearly uncorrelated variables called principal components (or sometimes, principal modes of variation). We perform PCA analysis based on the PCA plan provided by our customer, shown as **Figure S15**.

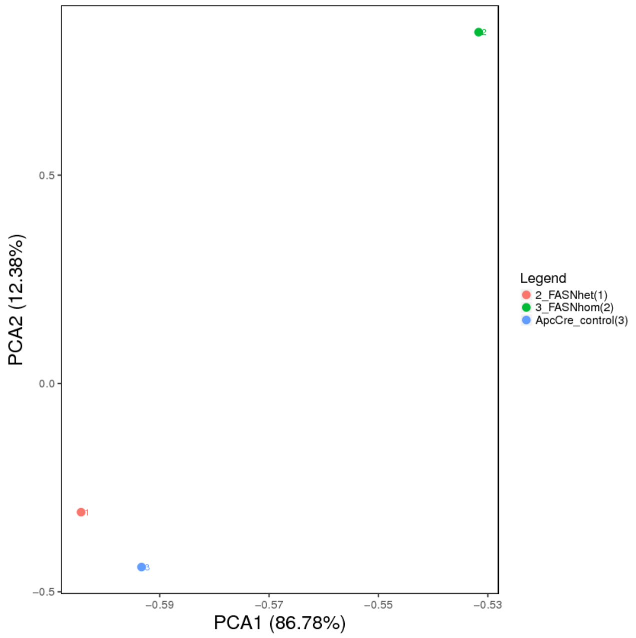

Figure S15 PCA analysis.

X axis represents the contributor rate of first component. Y axis represents the contributor rate of second component. Points represent each sample. The samples in one group shows the same color.

7.5 The Distribution of Gene Expression

Based on the expression information, we preform box plot to show the distribution of the gene expression level of each sample, besides we can observe the dispersion of the distribution, as shown in **Figure S16**. The density map can show the change of gene abundance and reflect the concentration of gene expression in the sample interval, as show in **Figure S17**.

© 2019 BGI All Rights Reserved.

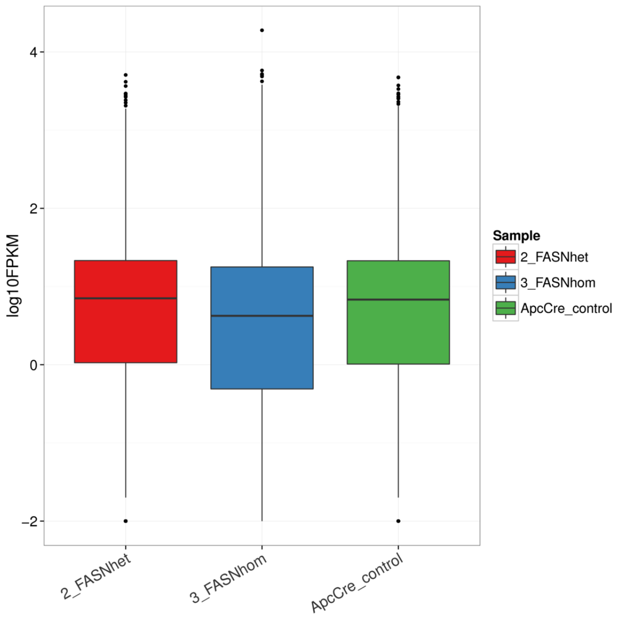

Figure S16 Gene expression Box-plot.

X axis represents the sample name. Y axis represents the log10FPKM value.

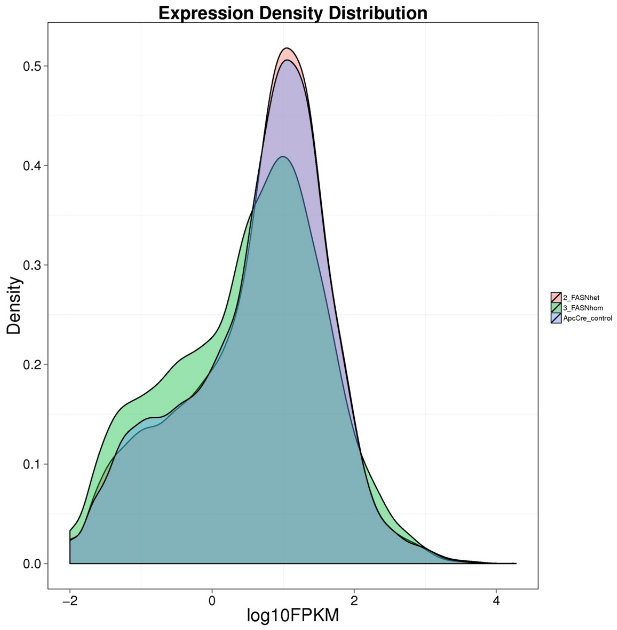

Figure S17 Gene expression density map.

X axis represents the log10FPKM value. Y axis represents the gene density.

To show the gene amount under different FPKM value, we calculate the gene amount under three different FPKM ranges, FPKM <= 1, FPKM 1~10, FPKM >= 10, shown as **Figure S18**.

© 2019 BGI All Rights Reserved.

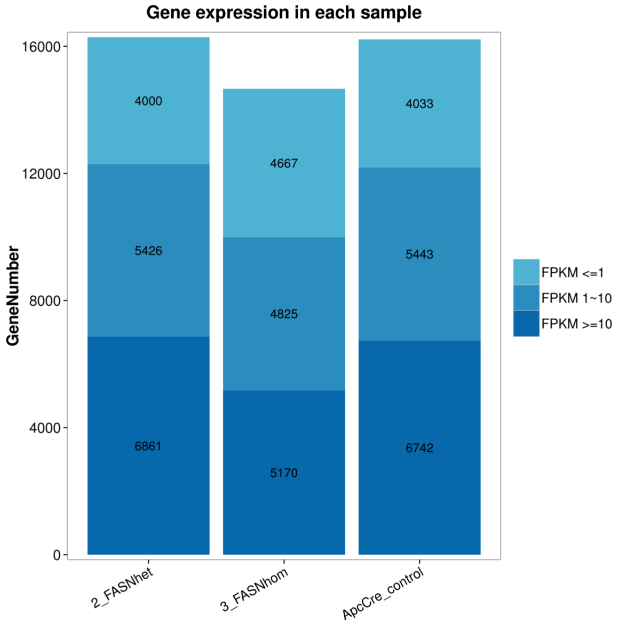

Figure S18 Gene expression distribution.

X axis represents the sample name. Y axis represents the gene amount. The dark color means the high expression level which FPKM value >= 10, while the light color means the low expression level which FPKM value <= 1.

7.6 Gene expression between samples and between groups

We also use venn diagram to display expressed gene between different samples and different groups , shown as **Figure S19**. The Venn diagram can show the number of genes specific expressed in a sample (groupand expressed in multiple samples (groups.

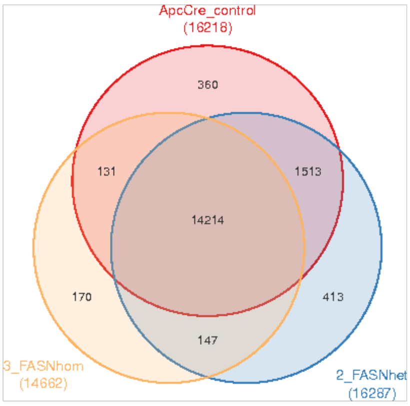

Figure S19 Venn diagram of gene expression between samples and between groups.

8 Circos Diagram

© 2019 BGI All Rights Reserved.

Based on the ***SNP*** , ***INDEL*** , gene expression result, we use Circos[11] to perform the analysis, shown as **Figure S20**.

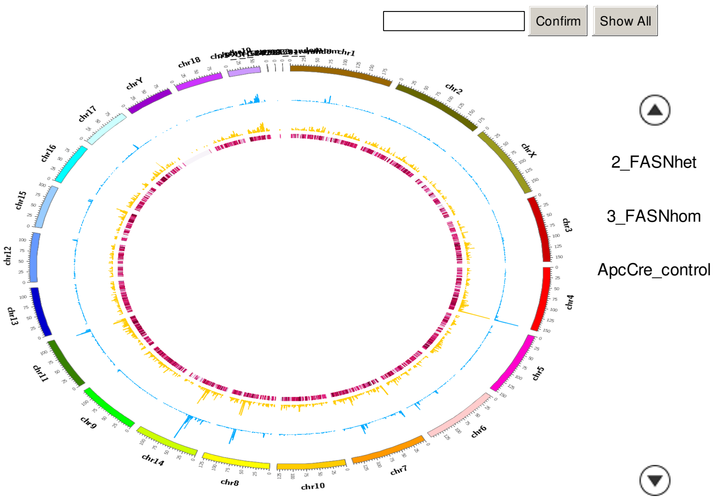

Figure S20 Circos diagram.

From the outside circle to the inner circle, the first circle represents chromosome, the second, third and forth circle represents the SNP amount, INDEL amount and FPKM value, respectively. The connection lines in the inner circle represent the gene fusion.(Red lines represent the gene fusion occurs between chromosomes, green lines represent the gene fusion which occurs within chromosomes, gene fusion analysis only for human samples.)

9 Gene expression cluster analysis

Genes with similar expression patterns usually have same functional correlation. We use cluster software and Euclidean distance matrix for the hierarchical clustering analysis of the expressed gene and sample program at the same time, The clustering results can be viewed with javaTreeview. Please enter the project result directory *BGI_result/Quantify/GeneExpression/GeneCluster*,the corresponding relations of each cluster group table *BGI_result/Quantify/GeneExpression/GeneClusterxp_cluster_correspond.xls*, open TreeView.jar and view the cdt file. In addition, we also provide a complete clustering graph for the intersection (if any) and the union gene of each expression clustering scheme, shown as **Figure S21**. This analysis could also be done online by your self. Online analysis link: http://www.heatmapper.ca/expression/, detail Instructions was introduced in method.

© 2019 BGI All Rights Reserved.

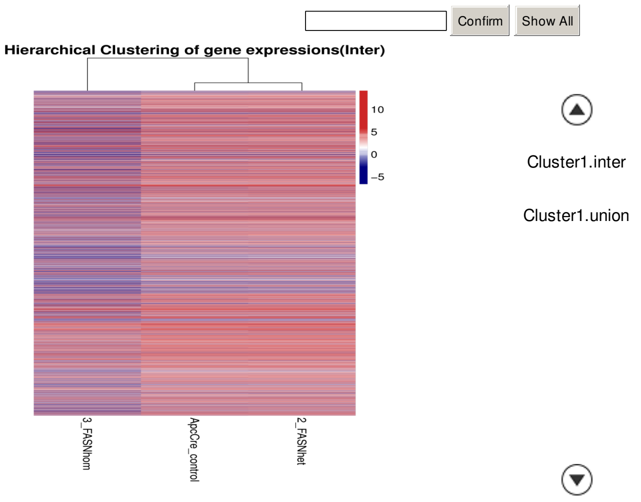

Figure S21 Cluster diagram of gene expression.

The gradient legend at the top right of the graph represents the FPKM value that has been logarithmically converted. Each column represents a sample, each row represents a gene, different colors represents different expression levels, red for high expression, and blue for low expression.

10 Time Series Analysis

In different time stages, some of the genes may have similar expression pattern. According to the gene expression information, those genes can be clustered into time related clusters. Those genes with the same gene expression pattern will be in the same gene cluster. It has been mentioned in some papers that time series analysis can be used to identify the tissue specific genes. The gene cluster results are shown in **Figure S22**. Details refer to : BGI_result/Quantify/GeneExpression/ Clustering_Mfuzz. Membership represents the value between 0~1, it can be used to evaluate if the genes follow the change trends of the clusters. If the membership value goes to 1, it means the gene follow the change trend of the clusters.

© 2019 BGI All Rights Reserved.

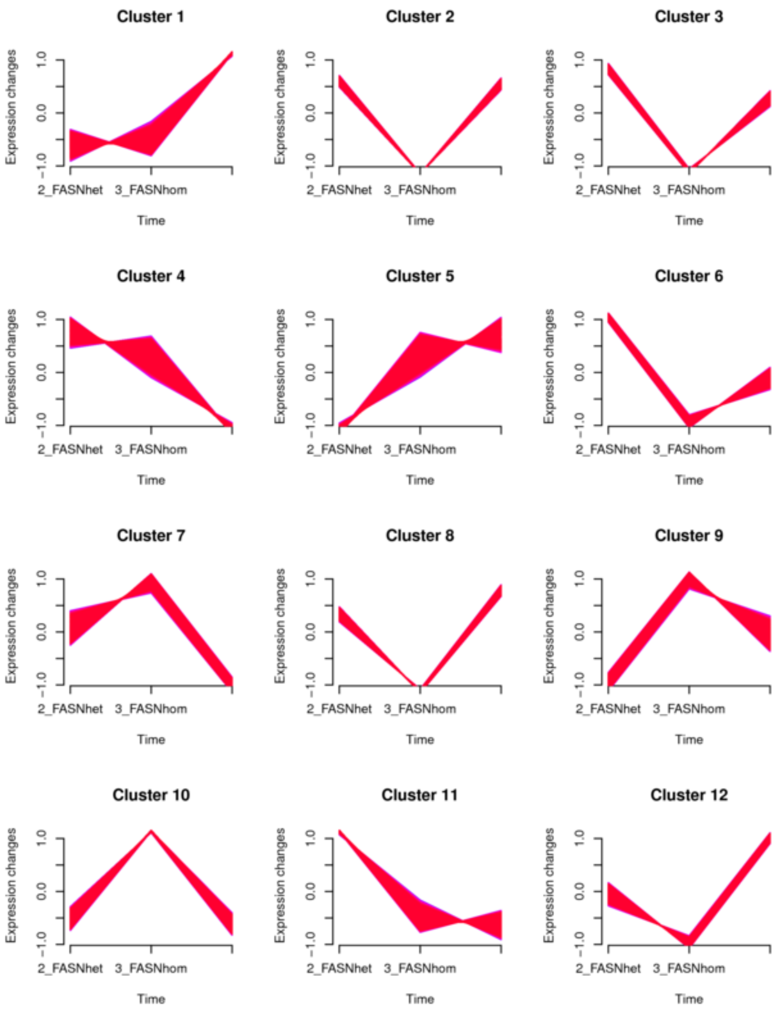

Figure S22 Time series analysis Mfuzz result.

X axis represents the timeline, Y axis represents the normalized gene expression level.

The genes in each cluster are shown in the below table:

Table S35 Distribution of genes in clusters （Download)

gene_id	cluster	2_FASNhet_fpkm	3_FASNhom_fpkm	ApcCre_control_fpkm	...
100039257	1	0	8.15	53.20	...
100039707	1	13.13	10.70	20.56	...
100041515	1	0.17	0.11	0.42	...
100041581	1	0.03	0	0.11	...

gene_id: Gene ID
cluster: Cluster ID
Sample(Group)1_fpkm: The FPKM value for sample(group)1
Sample(Group)2_fpkm: The FPKM value for sample(group)2
Sample(Group)3_fpkm: The FPKM value for sample(group)3
...: symbol, GO, Pathway and NR annotation

Table S36 Gene distribution in each clusters of 2_FASNhet.3_FASNhom.ApcCre_control: （Download)

© 2019 BGI All Rights Reserved.

Table S37 The membership value of the genes for 2_FASNhet.3_FASNhom.ApcCre_control:
(Download)

11 Differentially Expressed Gene Detection

Based on the gene expression level, we can identify the ***DEG*** (Differentially expression genes) between samples or groups. We use PossionDis algorithms to detect the DEGs, results shown as below:

Table S38 DEG result for ApcCre_control-VS-3_FASNhom.PossionDis_Method
(Download)

GeneID	Length	ApcCre_control-Expression	3_FASNhom-Expression	log2FoldChange(3_FASNhom/ApcCre_control)	FDR	Up/Down-Regulation(3_FASNhom/A
13226	419	0.01	43.66	12.09	2.61e-73	Up
226413	6174	0.16	259.58	10.66	0.00e+00	Up
20445	2396	0.05	66.44	10.38	0.00e+00	Up
232889	3390	0.42	203.00	8.92	0.00e+00	Up

GeneID: Gene ID
Length: Gene Length
Sample1-Expression: Gene expression in sample1
Sample2-Expression: Gene expression in sample2
log2FoldChange(sample2/sample1): The log2 value of ratio of Sample1-Expression to Sample2-Expression
FDR: False discovery rate
Up/Down-Regulation: Up/down-regulated
Pvalue: p-value
Symbol: Gene Symbol ID(Gene Name)

Table S39 DEG list for comparison 2_FASNhet-VS-3_FASNhom.PossionDis_Method: (Download)
Table S40 DEG list for comparison ApcCre_control-VS-2_FASNhet.PossionDis_Method: (Download)
Table S41 DEG list for comparison ApcCre_control-VS-3_FASNhom.PossionDis_Method: (Download)
Summary of DEGs is shown in **Figure S23**. We use MA plot, Volcano plot, Scatter plot and
Heatmap plot to show the distributions of DEGs in **Figure S24**, **Figure S25**, **Figure S26** and **Figure S27** respectively.

© 2019 BGI All Rights Reserved.

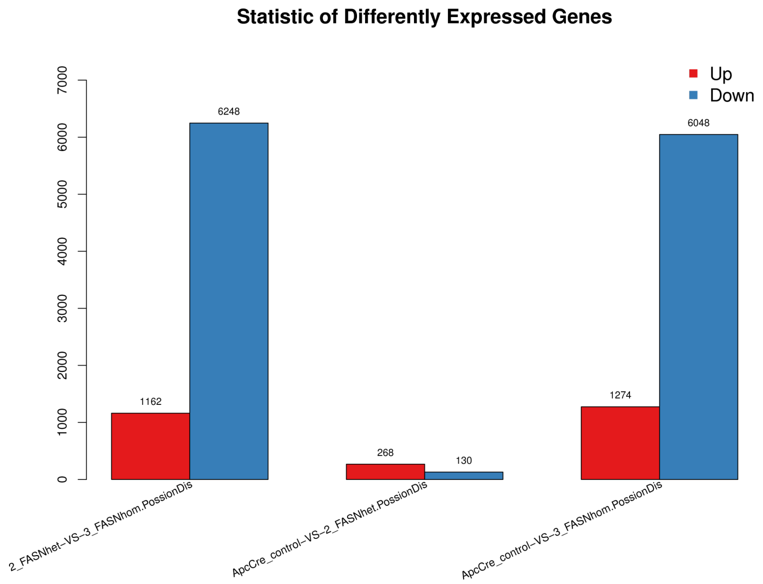

Figure S23 Summary of DEGs.

X axis represents comparison method between each group. Y axis represents DEG numbers. Red color represents up-regulated DEGs. Blue color represents down-regulated DEGs.

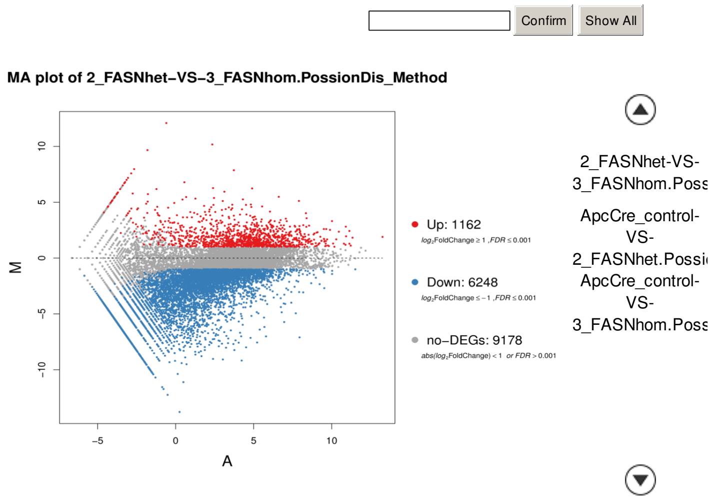

Figure S24 MA plot of DEGs.

X axis represents value A (log2 transformed mean expression level). Y axis represents value M (log2 transformed fold change). Red dots represent up-regulated DEGs. Blue dots represent down-regulated DEGs. Gray points represent non-DEGs.

© 2019 BGI All Rights Reserved.

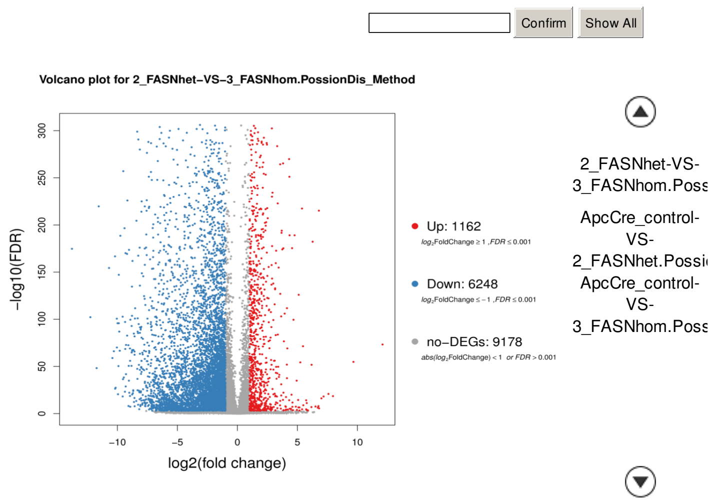

Figure S25 Volcano plot of DEGs.

X axis represents log2 transformed fold change. Y axis represents -log10 transformed significance. Red points represent up-regulated DEGs. Blue points represent down-regulated DEGs. Gray points represent non-DEGs.

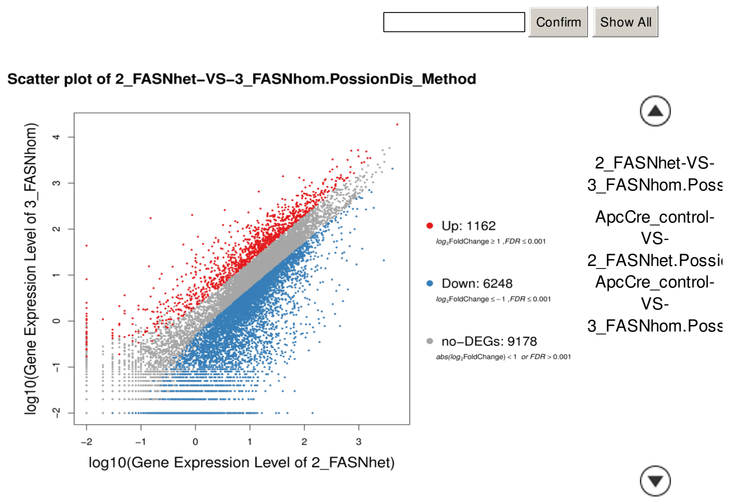

Figure S26 Scatter plot of DEGs.

X Y axis represents log10 transformed gene expression level, red color represents the up-regulated genes, blue color represents the down-regulated genes, gray color represents the non-DEGs.

© 2019 BGI All Rights Reserved.

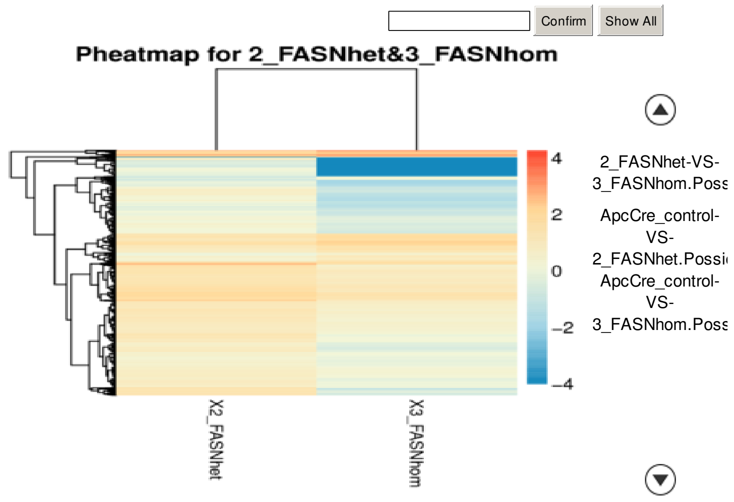

Figure S27 Heatmap of DEGs.

X axis represents the sample. Y axis represents the DEGs. The color represents the log10 transformed gene expression level. (The dark color means the high expression level while the light color means the low expression level.)

12 Venn Diagram of DEG

We perform DEGs by Venn diagrams, as shown in **Figure S28**.

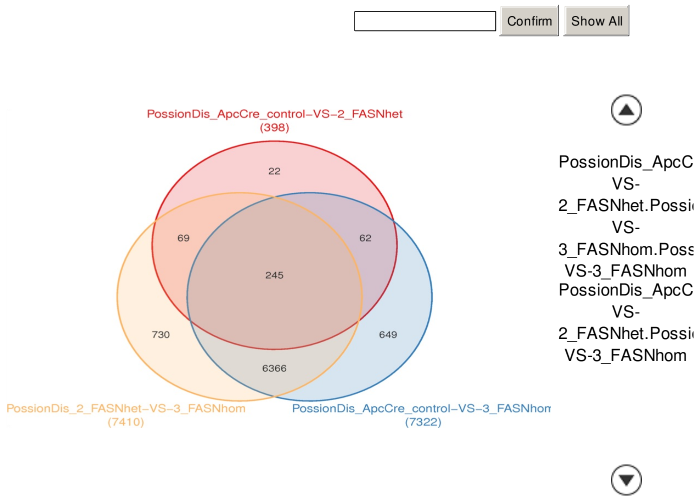

Figure S28 Venn diagram of DEGs.

The red number represents the up-regulated gene amount, blue number represents the down-regulated gene amount.

13 Clustering Analysis of DEG

We perform hierarchical clustering for DEGs ,shown as **Figure S29**.

© 2019 BGI All Rights Reserved.

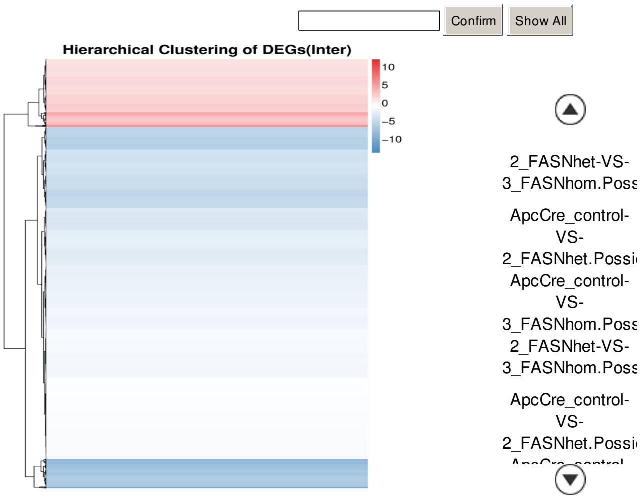

Figure S29 Heatmap of hierarchical clustering of DEGs.

X axis represents each comparing sample. Y axis represents DEGs. Coloring indicates the log2 transformed fold change (high: red, low: blue).

The ordered ***DEG*** lists after hierarchical clustering are shown as tables below (The file name suffix '.inter' or '.union' indicate the file contents are intersection or union of DEGs among different comparison groups. See Cluster list format in help page **Cluster list format**):

Table S42 Clustering DEGs list of 2_FASNhet-VS-3_FASNhom.PossionDis.inter: (Download)

Table S43 Clustering DEGs list of 2_FASNhet-VS-3_FASNhom.PossionDis.union: (Download)

Table S44 Clustering DEGs list of ApcCre_control-VS-2_FASNhet.PossionDis.inter: (Download)

Table S45 Clustering DEGs list of ApcCre_control-VS-2_FASNhet.PossionDis.union: (Download)

Table S46 Clustering DEGs list of ApcCre_control-VS-3_FASNhom.PossionDis.inter: (Download)

Table S47 Clustering DEGs list of ApcCre_control-VS-3_FASNhom.PossionDis.union: (Download)

14 Gene Ontology Analysis of DEG

With DEGs, we perform ***Gene Ontology*** (GO) classification and functional enrichment. GO has three ontologies: molecular biological function, cellular component and biological process. We would perform functional enrichment respectively. The GO classification results are shown as **Figure S30**. The GO enrichment results are shown as **Figure S31**. The GO classification of up-regulated and down-regulated genes are shown as **Figure S32**. (Click help page **How to read DEG GO enrichment analysis result** to know how to read the GO analysis result)

© 2019 BGI All Rights Reserved.

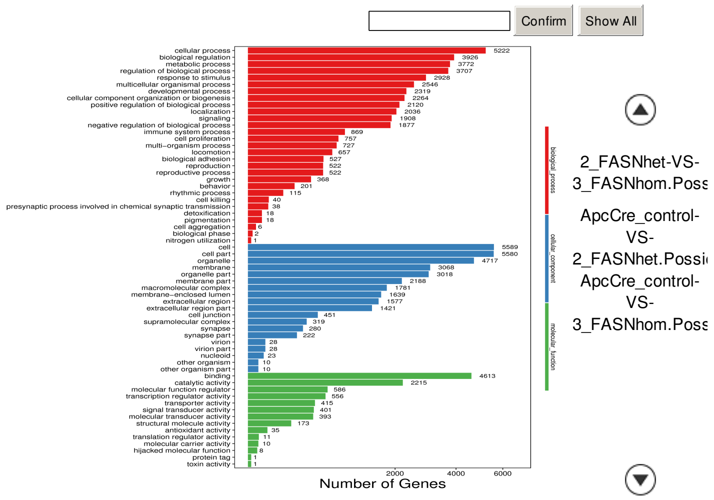

Figure S30 GO classification of DEGs.

X axis represents number of DEG. Y axis represents GO term.

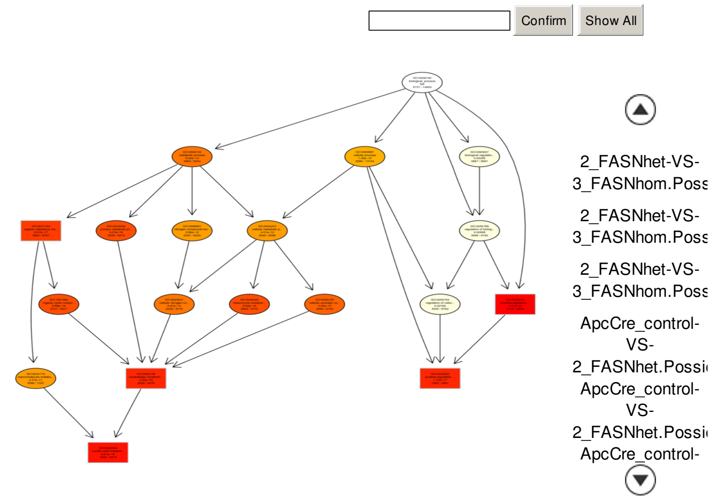

Figure S31 GO functional enrichment of DEGs .

We use DAG (Directed Acyclic Graph) to show the GO enrichment result. Each node shows the name of the GO term and the p-value. The darker (red) the color is, the lower p-value which indicates the more significant enrichment.

© 2019 BGI All Rights Reserved.

Confirm Show All

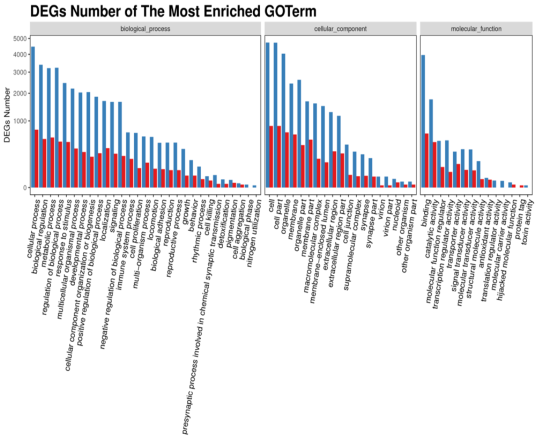

2_FASNhet-VS-3_FASNhom.Poss

ApcCre_control-VS-2_FASNhet.Possi

ApcCre_control-VS-3_FASNhom.Poss

Figure S32 GO classification of up-regulated and down-regulated genes.

X axis represents GO term. Y axis represents the amount of up/down-regulated genes.

Confirm Show All

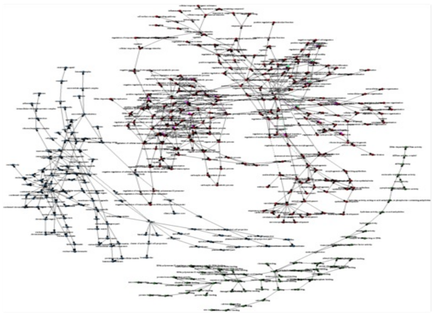

2_FASNhet-VS-3_FASNhom.Poss

ApcCre_control-VS-2_FASNhet.Possi

ApcCre_control-VS-3_FASNhom.Poss

Figure S33 GOTerm relationship network.

Each node represents a GOterm, the different colors represent the different functional classes to which it belongs, red for the biological process, blue for the cellular component, and green for the molecular function. The darkness indicates a significant enrichment (Qvalue <0.01) of GOterm, The lightness indicates no significant enrichment of GOterm,the gray indicates no enrichment of GOterm . Similar to a directed acyclic graph, it shows the relationship between the GOterm, solid arrows shows contain relationship, the dashed arrows shows control relations, red dotted line is positive controlled, green dotted line is negative control. (Through the page map you can be free to choose or adjust the location of the gene. Please click the "Download" link on the following page for more details.)

Table S48 2_FASNhet-VS-3_FASNhom.PossionDis_Method.network web page map. (Download)

Table S49 ApcCre_control-VS-2_FASNhet.PossionDis_Method.network web page map. (Download)

Table S50 ApcCre_control-VS-3_FASNhom.PossionDis_Method.network web page map. (Download)

© 2019 BGI All Rights Reserved.

Table S51 Web version of the report file (display page version report). (Download)

15 Pathway Analysis of DEG

With DEGs, we perform KEGG pathway classification and functional enrichment. The pathway classification results are shown as **Figure S34**, and the pathway functional enrichment results are shown as **Figure S35**. The pathway functional enrichment result for up/down regulation genes are shown as **Figure S36** (click help page **How to read DEG pathway enrichment analysis result** to know how to read the pathway analysis result).

Table S52 Pathway functional enrichment results. (Download)

#Pathway	ApcCre_control-VS-3_FASNhom.PossionDis_Method (6833)	All-gene (18873)	Pvalue	Qvalue	Pathway ID
Ribosome biogenesis in eukaryotes	71	108	3.979271e-10	1.333056e-07	ko03008
Cell cycle	86	149	6.729032e-08	1.127113e-05	ko04110
HTLV-I infection	164	335	9.792565e-07	1.008722e-04	ko05166
DNA replication	35	50	1.204444e-06	1.008722e-04	ko03030

#Pathway: The name of the pathway
group_Method: The number of DEGs which annotated to specific pathway
All-Gene: The number of genes which annotated to specific pathway
Pvalue: p-value
Qvalue: corrected p-value
Pathway ID: The ID of the pathway

Table S53 Pathway functional enrichment results of 2_FASNhet-VS-3_FASNhom.PossionDisPathway (Download)

Table S54 Pathway functional enrichment results of ApcCre_control-VS-2_FASNhet.PossionDisPathway (Download)

Table S55 Pathway functional enrichment results of ApcCre_control-VS-3_FASNhom.PossionDisPathway (Download)

Confirm Show All

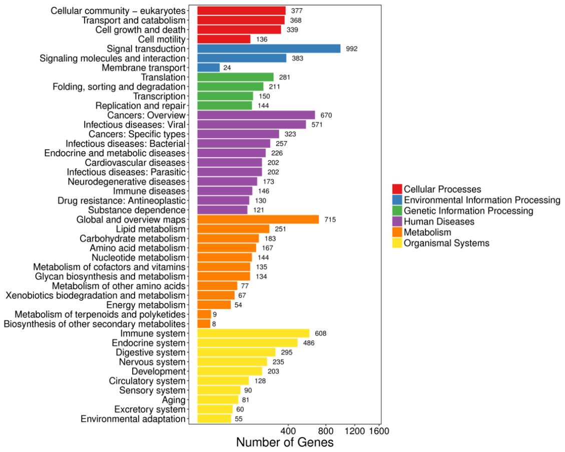

2_FASNhet-VS-3_FASNhom.Poss
ApcCre_control-VS-2_FASNhet.Possi
ApcCre_control-VS-3_FASNhom.Poss

Figure S34 Pathway classification of DEGs.

X axis represents number of DEG. Y axis represents functional classification of KEGG. There are seven branches for KEGG pathways: Cellular Processes, Environmental Information Processing, Genetic

© 2019 BGI All Rights Reserved.

Information Processing, Human Disease (For animals only), Metabolism, Organismal Systems and Drug Development.

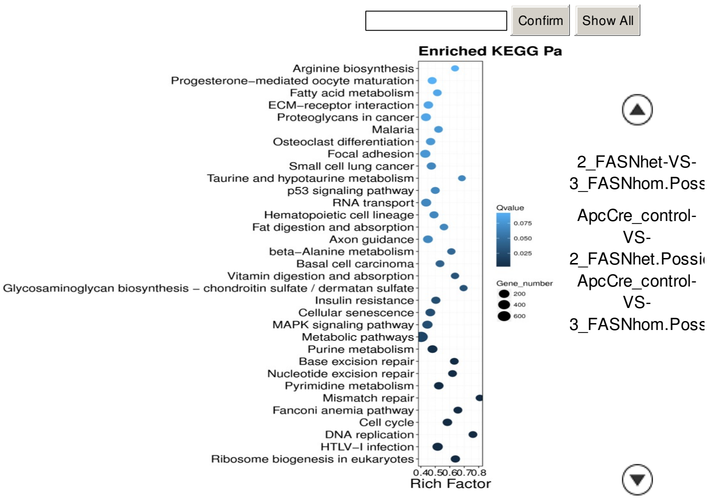

Figure S35 Pathway functional enrichment of DEGs.

X axis represents enrichment factor. Y axis represents pathway name. The color indicates the q-value (high: white, low: blue), the lower q-value indicates the more significant enrichment. Point size indicates DEG number (The bigger dots refer to larger amount). Rich Factor refers to the value of enrichment factor, which is the quotient of foreground value (the number of DEGs) and background value (total Gene amount). The larger the value, the more significant enrichment.

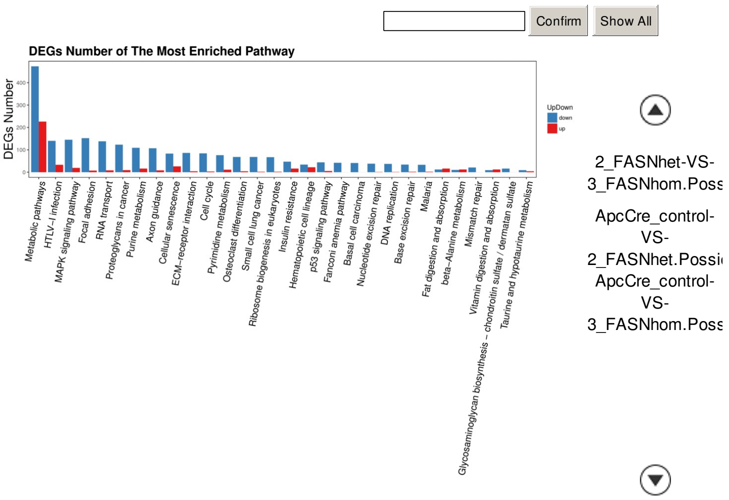

Figure S36 Pathway functional enrichment result for up/down regulation genes.

X axis represents the terms of Pathway. Y axis represents the number of up/down regulation genes.

© 2019 BGI All Rights Reserved.

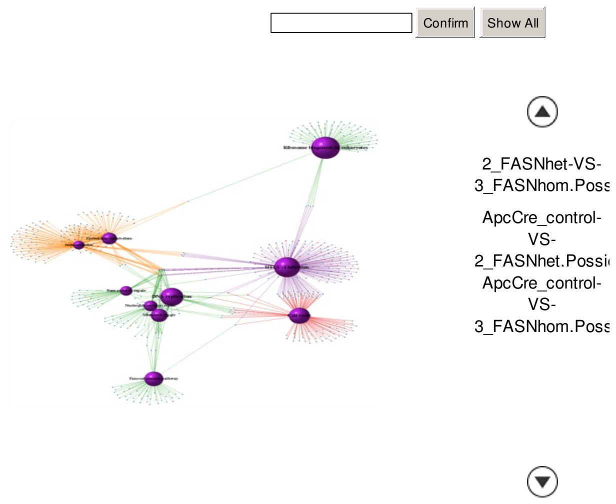

Figure S37 KEGG-DEG (differential expression gene) relationship network.

The red and blue dots represent the up-regulated and down-regulated genes, respectively. Purple ball indicates that the top ten enriched pathways, the darkness indicates a significant enrichment (Qvalue <0.01), and the light color indicates enrichment but is not significant. The larger the area, the higher the degree of enrichment. Different color lines represent different classes of Pathway, red for Cellular Processes, Blue for Environmental Information Processing, Green for Genetic Information Processing, Purple for Human Disease (Human Disease) Only animals), orange for metabolism (Metabolism), yellow for organic system, brown for drug development. (Through the page map,you can be free to choose or adjust the location of the gene, please click on the bottom of the page map "download" link.)

Table S56 2_FASNhet-VS-3_FASNhom.PossionDis_Method.path.xls.network network page map. (Download)

Table S57 ApcCre_control-VS-2_FASNhet.PossionDis_Method.path.xls.network network page map. (Download)

Table S58 ApcCre_control-VS-3_FASNhom.PossionDis_Method.path.xls.network network page map. (Download)

Table S59 Web version of the report file (display page version report). (Download)

16 Transcription Factor Prediction of DEG

For animals and plants, we predict the differentially expressed genes with the ability to encode transcription factor (***TF***)(click to view the help page for **TF**). At the same time, we classify the family of transcription factors to which the differentially expressed genes belong, and the results are shown in **Figure S38**. And the expression levels of transcription factors in each sample were clustered and the results are shown in **Figure S39**. The transcription factor corresponding to the differential gene is shown in **Figure S40**. The list of differentially expressed genes with the ability to encode transcription factors in this project is as follows:

Table S60 TF coding DEGs list (Download)

GeneID	Transcripts	TF_family
240590	NM_177360	DM
327992	NM_001045527	HSF
20646	NM_001349695	Homeobox

© 2019 BGI All Rights Reserved.

20646	NM_013670	Homeobox

GeneID: Gene ID
Transcripts: Transcript ID
TF_family: Transcription factor family
For details, see the help page **TF**.

Table S61 TF coding DEGs list of 2_FASNhet-VS-3_FASNhom.PossionDis_Method: (Download)

Table S62 TF coding DEGs list of ApcCre_control-VS-2_FASNhet.PossionDis_Method: (Download)

Table S63 TF coding DEGs list of ApcCre_control-VS-3_FASNhom.PossionDis_Method: (Download)

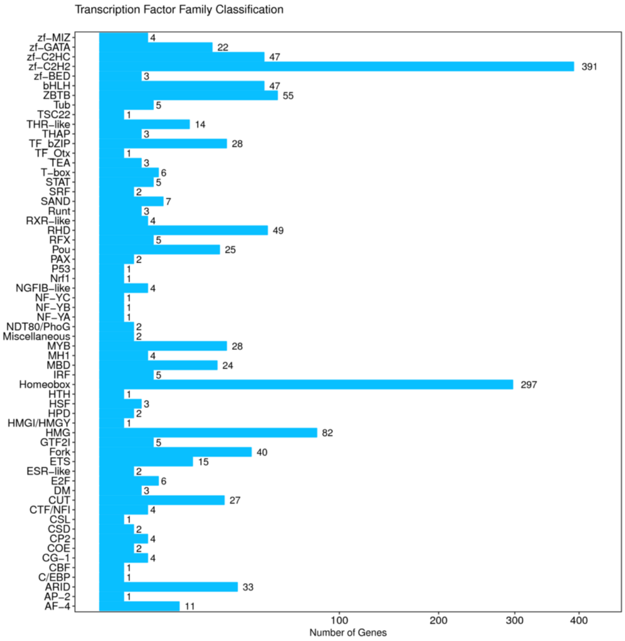

Figure S38 DEGs classification on TF family.

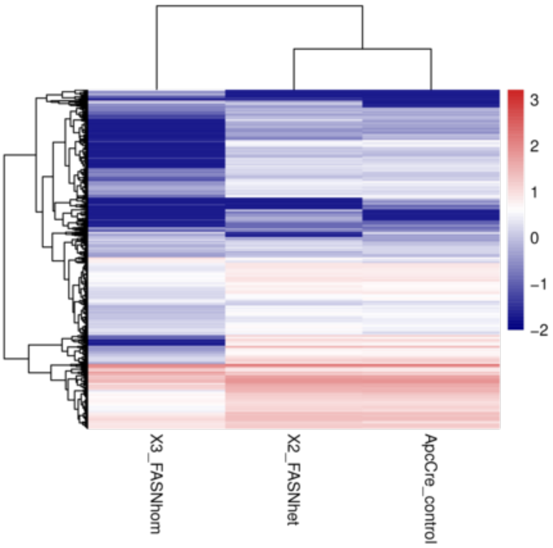

Figure S39 Expression heatmap of TF coding DEGs.

© 2019 BGI All Rights Reserved.

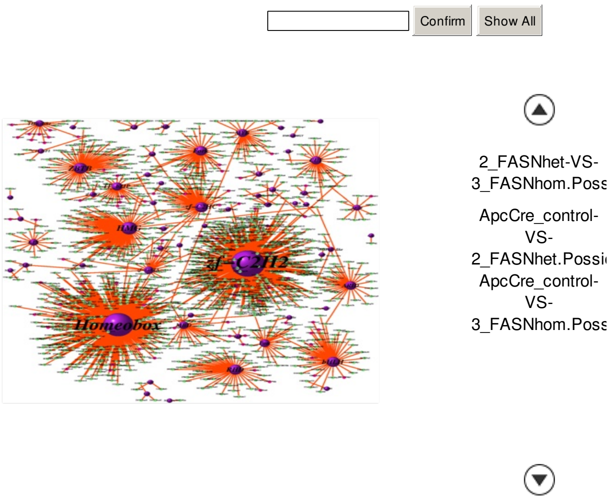

Figure S40 TF-DEG (differentially expressed genes) network.

The red and green dots represent the up-regulated and down-regulated DEGs, respectively. Purple ball represent transcription factor, the greater the node the more DEGs the transcription factor regulate. (Through the page map,you can be free to choose or adjust the location of the gene, please click on the bottom of the page map "download" link.)

Table S64 2_FASNhet-VS-3_FASNhom.PossionDis_Method.network network page map. (Download)
Table S65 ApcCre_control-VS-2_FASNhet.PossionDis_Method.network network page map. (Download)
Table S66 ApcCre_control-VS-3_FASNhom.PossionDis_Method.network network page map. (Download)
Table S67 Web version of the report file (display page version report). (Download)

17 Protein-Protein Interaction Networks of DEG

We use STRING[19] database to analyze the protein and protein interaction and construct the interaction networks of DEGs. We select the top 100 interaction networks to draw the picture, see **Figure S41**. We also provide the input files for Cytoscape network analysis directly. Cytoscape is a software platform for visualizing complex networks and integrating these with any type of attribute data.

Table S68 Protein-protein interaction result of DEGs (Download)

gene1	gene2	protein_cluster1	protein_cluster2	score
21385	15417	10090.ENSMUSP00000000095	10090.ENSMUSP00000000010	163
21387	15417	10090.ENSMUSP00000000096	10090.ENSMUSP00000000010	161
21387	21385	10090.ENSMUSP00000000096	10090.ENSMUSP00000000095	183
18053	15417	10090.ENSMUSP00000000122	10090.ENSMUSP00000000010	216

gene1: Interaction gene 1
gene2: Interaction gene 2
protein_cluster1: Protein encoded by Gene1 in the STRING database
protein_cluster2: Protein encoded by Gene2 in the STRING database
score: The larger the value, the more reliable result

Table S69 Protein-protein interaction result of 2_FASNhet-VS-

© 2019 BGI All Rights Reserved.

3_FASNhom.PossionDis_Method: (Download)

Table S70 Protein-protein interaction result of ApcCre_control-VS-2_FASNhet.PossionDis_Method: (Download)

Table S71 Protein-protein interaction result of ApcCre_control-VS-3_FASNhom.PossionDis_Method: (Download)

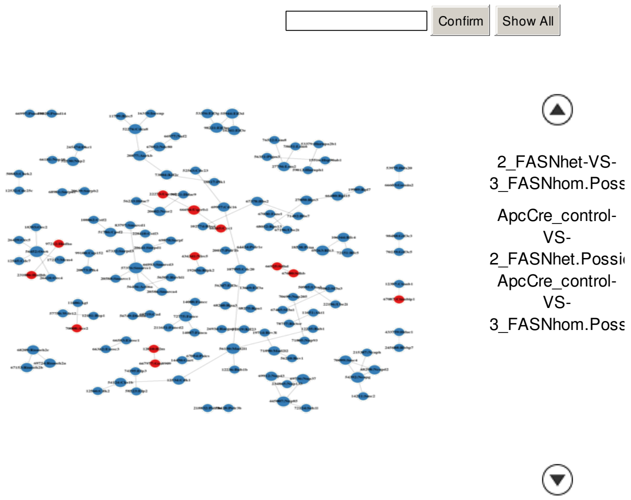

Figure S41 Protein-protein interaction network.

The red dots refer to up-regulated genes, while the blue dots refer to down-regulated genes. The size of the circle indicates the number of interactions.

Table S72 2_FASNhet-VS-3_FASNhom.PossionDis_Method.network network page map.

(Download)

Table S73 ApcCre_control-VS-2_FASNhet.PossionDis_Method.network network page map.

(Download)

Table S74 ApcCre_control-VS-3_FASNhom.PossionDis_Method.network network page map.

(Download)

Table S75 Web version of the report file (display page version report). (Download)

Methods

1 Experiment and Bioinformatics Workflow

1.1 Experiment Workflow

Total RNA sample QC

We use Agilent 2100 Bio analyzer (Agilent RNA 6000 Nano Kit) to do the total RNA sample QC: RNA concentration, RIN value,28S/18S and the fragment length distribution. For plant and fungi samples, we use NanoDrop^{TM}to identify the purity of the RNA samples.

Library construction

© 2019 BGI All Rights Reserved.

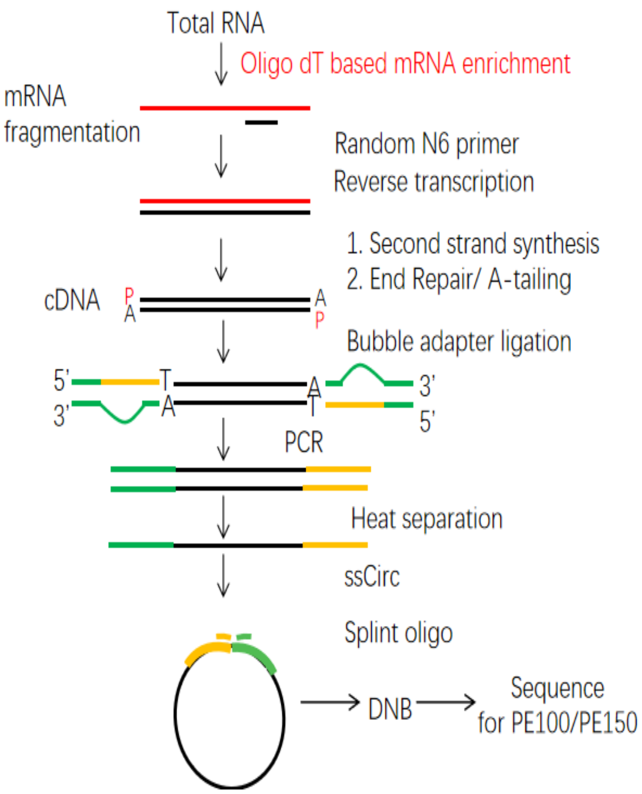

Figure S1 Experimental workflow.

The first step in the workflow involves purifying the poly-A containing mRNA molecules using poly-T oligo-attached magnetic beads. Following purification, the mRNA is fragmented into small pieces using divalent cations under elevated temperature. The cleaved RNA fragments are copied into first strand cDNA using reverse transcriptase and random primers. This is followed by second strand cDNA synthesis using DNA Polymerase I and RNase H. These cDNA fragments then have the addition of a single 'A' base and subsequent ligation of the adapter. The products are then purified and enriched with PCR amplification. We then quantified the PCR yield by Qubit and pooled samples together to make a single strand DNA circle (ssDNA circle), which gave the final library. DNA nanoballs (DNBs) were generated with the ssDNA circle by rolling circle replication (RCR) to enlarge the fluorescent signals at the sequencing process. The DNBs were loaded into the patterned nanoarrays and pair-end reads of 100 bp were read through on the DNBseq platform for the following data analysis study. For this step, the DNBseq platform combines the DNA nanoball-based nanoarrays and stepwise sequencing using Combinational Probe-Anchor Synthesis Sequencing Method.

1.2 Bioinformatics Workflow

Firstly, we removed the reads mapped to rRNAs and get rawdata, then we filter the low quality reads (More than 20% of the bases qualities are lower than 10), reads with adaptors and reads with unknown bases (N bases more than 5%) to get the clean reads. Then we map those clean reads onto reference genome(mm10_UCSC_20180903), followed with novel gene prediction, ***SNP*** & ***INDEL*** calling and gene splicing detection. Finally, we identify DEGs (differentially expressed genes) between samples and do clustering analysis and functional annotations. The analysis pipeline is shown in **Figure S2**.

© 2019 BGI All Rights Reserved.

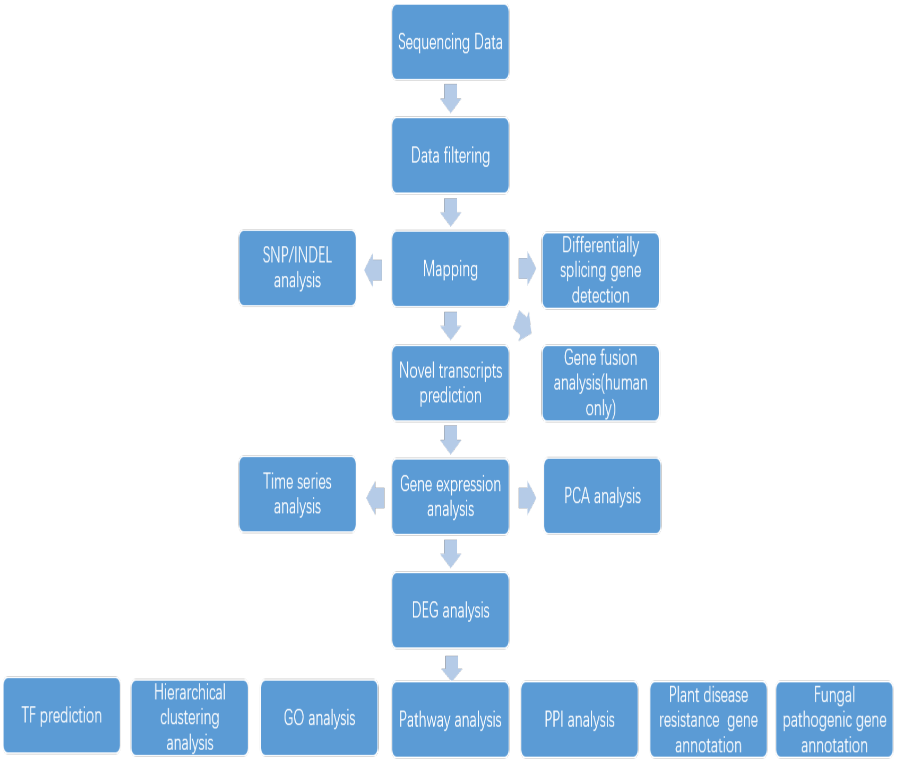

Figure S2 Bioinformatics workflow.

2 Sequencing Reads Filtering

We use internal software SOAPnuke to filter reads, followed as:

1) Remove reads with adaptors;

2) Remove reads in which unknown bases(N) are more than 0.1%;

3) Remove low quality reads (we define the low quality read as the percentage of base which quality is lesser than 20 is greater than 40% in a read).

After filtering, the remaining reads are called "Clean Reads" and stored in FASTQ format [1](see FASTQ Format in help page **FASTQ Format**).

Software information:

SOAPnuke:
version： v1.5.2
parameters: -n 0.001 -l 20 -q 0.4 -A 0.25
website: https://github.com/BGI-flexlab/SOAPnuke

3 Genome Mapping

We use HISAT2 (Hierarchical Indexing for Spliced Alignment of Transcripts) to do the mapping step. For HISAT2 which is much faster, sensitive and high accuracy analysis
software. The mapping method is shown as **Figure S3**[2].

© 2019 BGI All Rights Reserved.

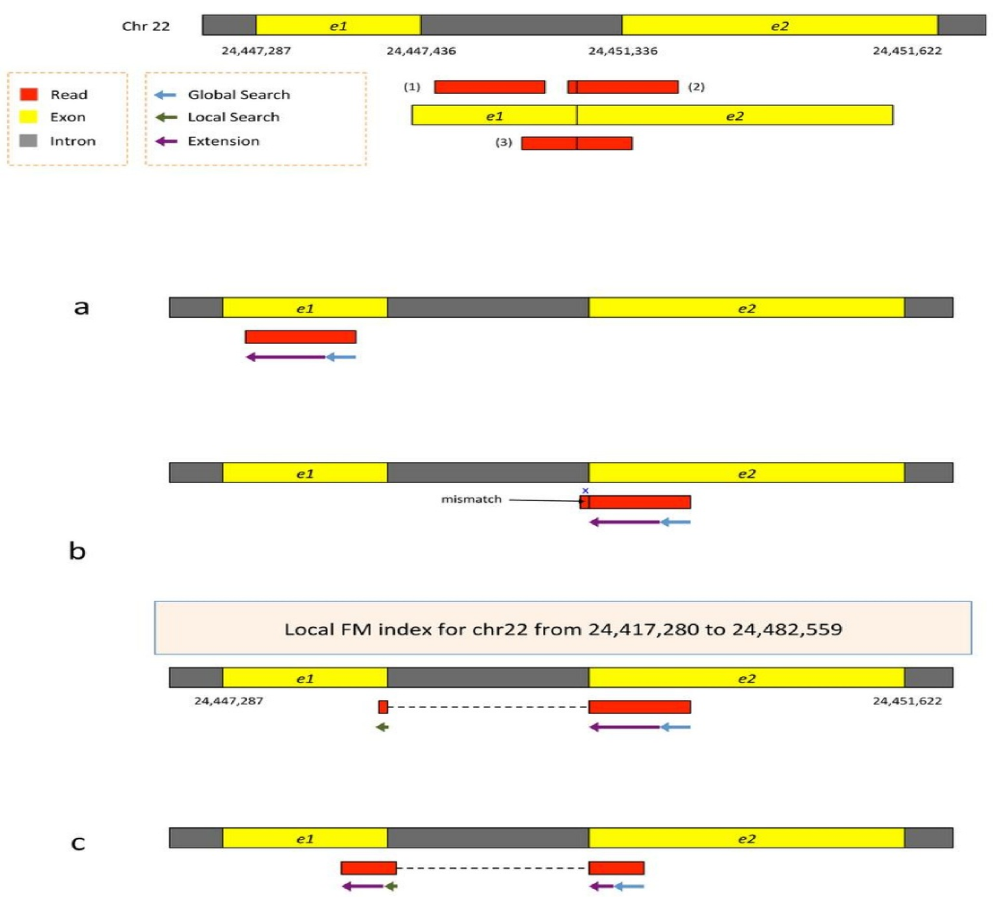

Figure S3 HISAT2 mapping demo show.

Software information:

HISAT2:

Version: v2.0.4

Parameters: --phred33 --sensitive --no-discordant --no-mixed -I 1 -X 1000

Website: http://www.ccb.jhu.edu/software/hisat

4 Novel Transcript Prediction

We use StringTie[3] to reconstruct transcripts, and use Cuffcompare (Cufflinks [4] tools) to compare reconstructed transcripts to reference annotation, after that, we select 'u','i','o','j' class code types as novel transcripts, class code type details is shown

as **Table S1**. And then, we use CPC [5]to predict coding potential of novel transcripts, then we merge coding novel transcripts with reference transcripts to get a complete reference, and downstream analysis will base on this reference. StringTie is an much faster and accurate software for transcriptome assembly, compared to Cufflinks software [2].The pipeline for transcriptome assembly based on reference please see

Figure S4[6].

Table S1 Explanation of class code. （Download)

Class_Code	Explanation
u	Unknown, intergenic transcript.

© 2019 BGI All Rights Reserved.

i	A transfrag falling entirely within a reference intron.
o	Generic exonic overlap with a reference transcript.
j	Potentially novel isoform (fragment): at least one splice junction is shared with a reference transcript.

Please refer to Cufflinks Website for class code details.

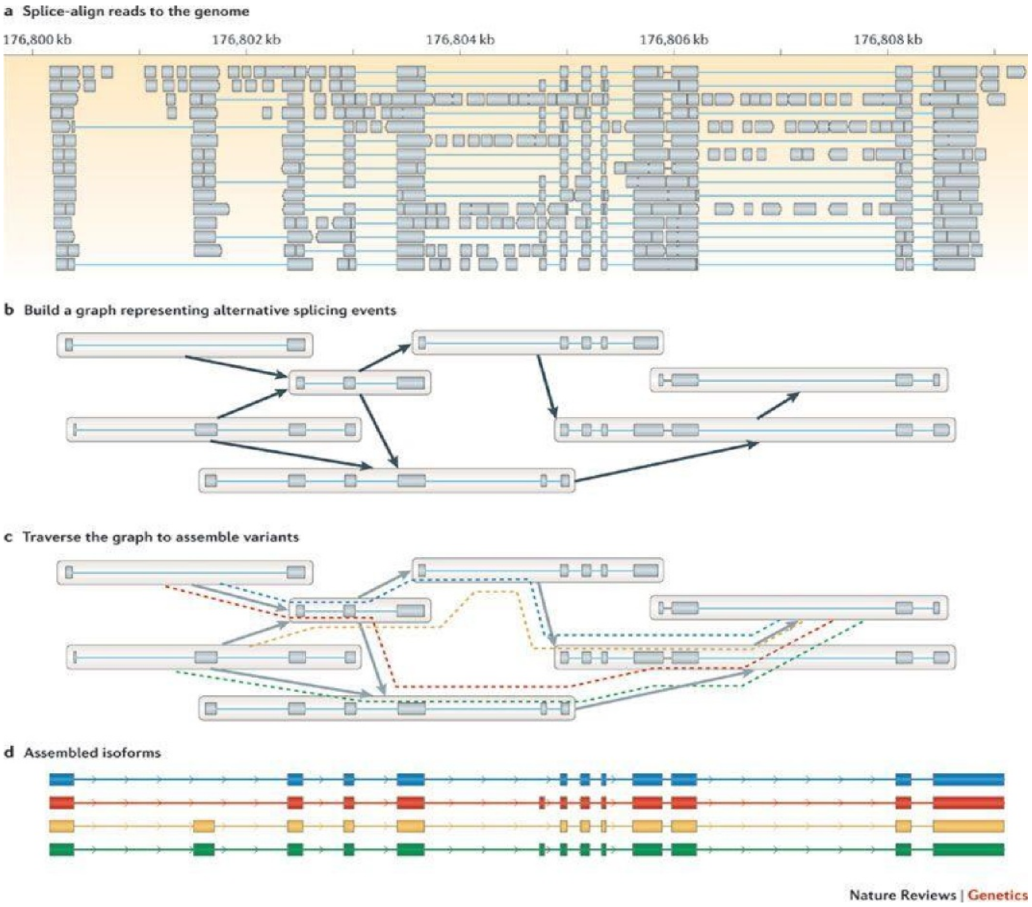

Figure S4 Transcriptome assembly based on reference.

Software information:

StringTie:

Version: v1.0.4

Parameters: -f 0.3 -j 3 -c 5 -g 100 -s 10000 -p 8

Website: http://ccb.jhu.edu/software/stringtie

Cufflinks:

Version: v2.2.1

Parameters: -p 12

Website: http://cole-trapnell-lab.github.io/cufflinks

CPC:

Version: v0.9-r2

Parameters: Default

Website: http://cpc.cbi.pku.edu.cn

5 SNP and INDEL Detection

With genome mapping result, we use GATK[7] to call ***SNP*** and ***INDEL*** for each

© 2019 BGI All Rights Reserved.

sample. After filtering out the unreliable sites, we get the final ***SNP*** and ***INDEL*** in VCF format.

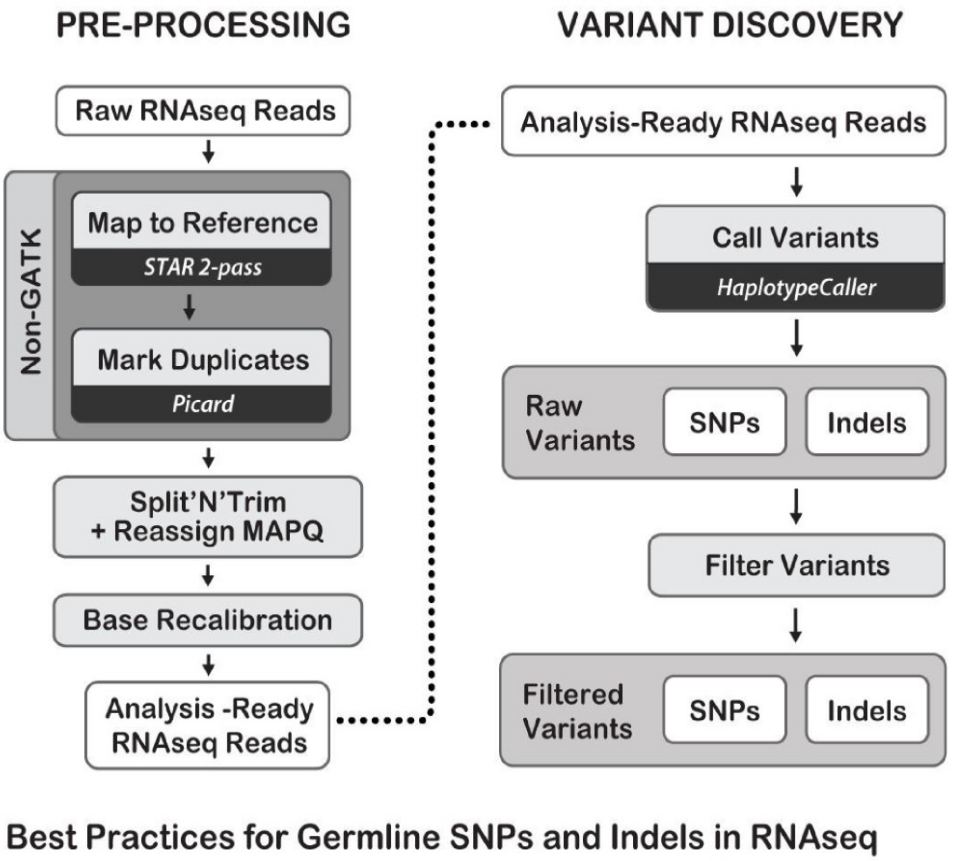

Figure S5 Pipeline for calling SNP and INDEL in RNAseq.

6 Differentially Splicing Gene Detection

It is important to distinguish differential isoform relative abundance, from differential isoform expression. Changes in relative abundance of isoforms, regardless of the expression change, indicate a splicing-related mechanism. On the other hand, there can be measurable changes in the expression of isoforms across samples, without necessarily changing the relative abundance, which possibly indicates a transcription-related mechanism. We use rMATS[8] to detect differentially splicing gene(that is differential isoform relative abundance between samples), a computational tool to detect differential alternative splicing events from RNA-Seq data, it calculates the inclusion isoform and skipping isofrom, shown as **Figure S6**. The statistical model of MATS calculates the P-value and false discovery rate (***FDR***) that the difference in the isoform ratio of a gene between two conditions, in our project, gene that with ***FDR*** <= 0.05 is defined as significant differentially splicing gene (***DSG***).

© 2019 BGI All Rights Reserved.

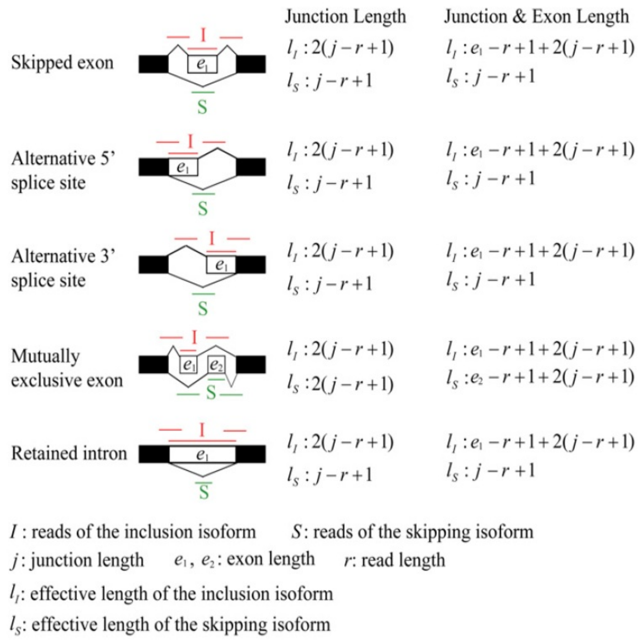

Figure S6 Relative abundance calculation of differential isoforms.

Software information:

rMATS:
Version: v4.0.2
Parameters: -t paired --nthread 8 --tstat 4
Website: http://rnaseq-mats.sourceforge.net

7 Gene Expression Analysis

We mapped clean reads to reference using Bowtie2[9], and then calculate gene expression level with ***RSEM*** [10]. ***RSEM*** is a software package for estimating gene and isoform expression levels from RNA-Seq data.

calculate pearson correlation between all samples using cor, perform hierarchical clustering between all samples using hclust, perform PCA analysis with all samples using princomp, and draw the diagrams with ggplot2 with fuctions of R.

Software information:

Bowtie2 **:**
Version: v2.2.5
Parameters: -q --phred33 --sensitive --dpad 0 --gbar 99999999 --mp 1,1 --np 1 --score-min L,0,-0.1 -I 1 -X 1000 --no-mixed --no-discordant -p 1 -k 200
Website: http://bowtie-bio.sourceforge.net/ ***Bowtie2*** /index.shtml
RSEM **:**
Version: v1.2.12
Parameters: default
Website: http://deweylab.biostat.wisc.edu/ ***RSEM***

8 Circos Diagram

© 2019 BGI All Rights Reserved.

Circos is a software package for visualizing data and information[11]. We visualize ***SNP*** , ***INDEL*** , gene expression and gene fusion (Only for human samples) result based on Circos diagram.

Software information:

Circos:
Version: v0.69
Website: http://www.circos.ca

9 Gene expression cluster analysis

The clustering results were displayed with javaTreeview[14] using cluster[12][13] software to analyze the expression genes and sample scheme at the same time by using the Euclidean distance matrix as the matrix formula.

Software information:

cluster:
Version: v3.0
Parameters: -g 7 -e 7 -m a
Website: http://www.ncbi.nlm.nih.gov/pubmed/9843981

10 Time Series Analysis

Clustering analysis is a common method for gene expression analysis. There are two types of clustering: Hard clustering and soft clustering. We use Mfuzz[15] analysis software to do the soft cluster which is more suitable for gene expression data.

Software information:

Mfuzz:
Version: v2.34.0
Parameters: -c 12 -m 1.25
Website: http://mfuzz.sysbiolab.eu

11 DEG Detection

We detect DEGs with PossionDis as requested.PossionDis is based on the poisson distribution, performed as described at Audic S, et al.[16]

Software information:

PossionDis:
Parameters: Fold Change >= 2.00 and ***FDR*** <= 0.001

12 Hierarchical Clustering Analysis of DEG

We perform hierarchical clustering for DEGs using pheatmap, a function of R. For cluster more than two groups, we perform the intersection and union DEGs between them, respectively.

13 Gene Ontology Analysis of DEG

With the GO annotation result, we classify DEGs according to official classification, and

© 2019 BGI All Rights Reserved.

we also perfrom GO functional enrichment using phyper, a function of R. The pvalue calculating formula in hypergeometric test is:

$$P = 1 - \sum_{i=0}^{m-1} \frac{\binom{M}{i}\binom{N-M}{n-i}}{\binom{N}{n}}$$

See wiki for details https://en.wikipedia.org/wiki/Hypergeometric_distribution。

Then we calculate false discovery rate (***FDR***) for each pvalue, in general, the terms which ***FDR*** not larger than 0.01 are defined as significant enriched.

14 Pathway Analysis of DEG

With the KEGG annotation result, we classify DEGs according to official classification, and we also perform pathway functional enrichment using phyper, a function of R. The pvalue calculating formula in hypergeometric test is:

$$P = 1 - \sum_{i=0}^{m-1} \frac{\binom{M}{i}\binom{N-M}{n-i}}{\binom{N}{n}}$$

See wiki for details https://en.wikipedia.org/wiki/Hypergeometric_distribution.

Then we calculate false discovery rate (***FDR***) for each pvalue, in general, the terms which ***FDR*** not larger than 0.01 are defined as significant enriched.

15 Transcription Factor Prediction of DEG

We use getorf to find ORF of each ***DEG*** . For plants, we align ORF to ***TF*** domains (from PlntfDB) using hmmsearch\[17\]. For animals, we align ORF to animal ***TF*** database (AnimalTFDB) using DIAMOND\[18\].

Software information:

PlntfDB:

Version: v23.0

Website: http://plntfdb.bio.uni-potsdam.de/v3.0/

AnimalTFDB:

Version: v2.0

Website: http://www.bioguo.org/AnimalTFDB/

getorf:

Version: EMBOSS:6.5.7.0

Parameters: -minsize 150

Website: http://www.bioinformatics.nl/cgi-bin/emboss/help/getorf

hmmseach:

Version: v3.0

Parameters: default

Website: http://hmmer.org

DIAMOND:

© 2019 BGI All Rights Reserved.

Version: v0.8.31

Parameters: --more-sensitive --evalue 1e-5

Website:https://github.com/bbuchfink/diamond

16 PPI Analysis of DEG

We use DIAMOND\[18\] to map the DEGs to the STRING\[19\] database to obtain the interaction between ***DEG*** -encoded proteins using homology with known proteins. We select the top 100 interaction networks to draw the picture, for the entire interaction result we provide an input file that can be imported directly into Cytoscape for network analysis. Cytoscape is a software for complex network analysis and visualization. For more information, refer to the official documentation.

STRING:

Version: v10

Website: http://string-db.org/

DIAMOND:

Version: v0.8.31

Parameters(Running): --evalue 1e-5 --outfmt 6 --max-target-seqs 1 --more-sensitive

Parameters(Selecting): query coverage >= 50%、identity >= 40%

Website: https://github.com/bbuchfink/diamond

Help

1 FASTQ Format

The original image data is transferred into sequence data via ***base calling*** , which is defined as raw data or raw reads and saved as FASTQ file. Those FASTQ files are the original data provided for users, including detailed read sequences and the read quality information. In each FASTQ file, every read is described by four lines, listed as follows:

```
@CL100012105L2C001R003_48/1
CAGCCAGCCAGTGGCAGTGCGAGGTGGAGGAGGCAAACAAGTGTAATCGTTTATACATACCCACAGGTGTTAA
AAAGTAATCGAAGTACGAAGAGGAACA
+
FGFFFFFFFFFFFFFFFFFFFFFFFFFFFFFFFFFFFFGFGFFEFFFGFFFFFFFFFFFFFGF:FFFFCFFFFFGFGGFFEGFFFF
FFFFFFFFFFF>FFEFF
```

The first and third lines are sequences names generated by the sequence analyzer; the second line is sequence; the fourth line is ***sequencing quality*** value, in which each letter corresponds to the base in line 2;

For details , Pls check the website: url{ http://en.wikipedia.org/wiki/FASTQ_format } .

2 Relationship between sequencing error rate and sequencing quality value

The base quality is equal to ASCII value of the character in line 4 minus 64 (we call the quality system is Phred+64), e.g. the ASCII value of c is 99, then its base quality value is 35. Starting from the Illumina GA Pipeline v1.5, the base quality values range from 2 to 41. **Table1** demonstrates the relationship between ***sequencing error*** rate and the ***sequencing quality*** value. Specifically, if the ***sequencing error*** rate is denoted as E

© 2019 BGI All Rights Reserved.

and base quality value is denoted as Q, the relationship is as following formula:

$$SQ = -10 \times (\log\frac{E}{1-E})/(\log 10)$$
$$E = \frac{Y}{1+Y}$$
$$Y = \frac{SQ}{e^{-10 \times \log 10}}$$

Table S1 Relationship between sequencing error rate and sequencing quality value. (Download)

Sequencing Error Rate(%)	Sequencing Quality Value	Character(Phred+46)	Character(Phred+33)
1.00	20	T	5
0.10	30	^	?
0.01	40	h	I

Note: The quality value system of DNBseq is Phred+33.

3 VCF format

Variant Call Format (VCF) is a flexible and extendable format for variation data such as single nucleotide polymorphism (***SNP***), insertions/deletions (***INDEL***), copy number variants and structural variants. See details at UCSC website http://genome.ucsc.edu/FAQ/FAQformat.html#format10.1

Table S2 VCF instruction. (Download)

Item	Description
CHROM	Name of the chromosome (or contig, scaffold, etc.)
POS	Position in chromosome
ID	If the variants existed in dbSNP database, then the ID is the dbSNP ID.
REF	The base in the reference genome
ALT	The variant base
QUAL	Quality score
FILTER	Filter details
INFO	Other information

4 Differentially Splicing Gene format

Differentially Splicing Gene(***DSG***) result format is described in **Table S3**.

Table S3 Differentially Splicing Gene format instruction (Download)

Field	Description	Notes
GeneID	gene identity	-
Chr	chromosome	-
Strand	strand	-
Control-IC	inclusion junction counts for Control sample, replicates are separated by comma	-
Control-SC	skipping junction counts for Control sample, replicates are separated by comma	-
Treat-IC	inclusion junction counts for Treat sample, replicates are separated by comma	-
Treat-SC	skipping junction counts for Treat sample, replicates are separated by comma	-
Pvalue	statistical significance	-
FDR	false discovery ratio	-
longExonStart	the long exon start position on chromosome	for A3SS and A5SS event

© 2019 BGI All Rights Reserved.

longExonEnd	the long exon end position on chromosome	for A3SS and A5SS event
shortExonStart	the short exon start position on chromosome	for A3SS and A5SS event
shortExonEnd	the short exon end position on chromosome	for A3SS and A5SS event
flankingExonStart	the flanking exon start position on chromosome	for A3SS and A5SS event
flankingExonEnd	the flanking exon end position on chromosome	for A3SS and A5SS event
1stExonStart	the first exon start position on chromosome	for MXE event
1stExonEnd	the first exon end position on chromosome	for MXE event
2ndExonStart	the secend exon start position on chromosome	for MXE event
2ndExonEnd	the secend exon end position on chromosome	for MXE event
riExonStart	the intron-retained exon start position on chromosome	for RI event
riExonEnd	the intron-retained exon end position on chromosome	for RI event
skipExonStart	the skipped exon start position on chromosome	for SE event
skipExonEnd	the skipped exon end position on chromosome	for SE event
upstreamExonStart	the upstream exon start position on chromosome	for RI and SE event
upstreamExonEnd	the upstream exon end position on chromosome	for RI and SE event
downstreamExonStart	the downstream exon start position on chromosome	for RI and SE event
downstreamExonEnd	the downstream exon end position on chromosome	for RI and SE event
LongExonTranscripts	the transcripts that contain long exon, separated by comma	for A3SS and A5SS event
ShortExonTranscripts	the transcripts that contain short exon, separated by comma	for A3SS and A5SS event
1stExonTranscripts	the transcripts that contain first exon, separated by comma	for MXE event
2ndExonTranscripts	the transcripts that contain secend exon, separated by comma	for MXE event
RetainTranscripts	the transcripts that contain intron-retained exon, separated by comma	for RI event
AbandonTranscripts	the transcripts that exclude intron-retained exon, separated by comma	for RI event
InclusionTranscripts	the transcripts that include certain exon, seperated by comma	for SE event
SkippingTranscripts	the transcripts that exclude certain exon, seperated by comma	for SE event

5 How to view the cluster analysis report

Make sure that your computer has installed Java and then enter the cluster results directory BGI_result / Quantify / GeneExpression / GeneCluster, double-click to open the TreeView.jar, and then directly Ctrl + o (or click the upper left File-> open) select the appropriate cdt file , you can view the detailed clustering results .

For more instructions on javaTreeView, see the official document http://jtreeview.sourceforge.net/manual.html。

6 DEG list format

The result of differentially expressed genes format is described in **Table4**.

Table S4 Format description of DEGs. （Download)

Field	Description
GeneID	Gene ID
Length	Gene length
Sample1-Expression	Gene expression of control sample(s)
Sample2-Expression	Gene expression of treat sample(s)
log2FoldChange(Sample2/Sample1)	log2 transformed fold change between control and treat samples
Pvalue	Statistic of pvalue(PossionDis or DEseq2 method used)
FDR	Statistic of false discovery rate(PossinoDis method used)
Padj	Statistic of adjusted pvalue(DEseq2 method used)
PPEE	Statistic of posterior probability of being equivalent expression(EBseq method used)
Probability	Statistic of probability of being DEG(NOIseq method used)

© 2019 BGI All Rights Reserved.

Up/Down-Regulation(Sample2/Sample1)	Flags indicate up-regulated DEG(Up) or down-regulated DEG(Down) or non-DEG(*)
...	Gene symbol, GO, KEGG and NR annotation

7 Cluster list format

The format of cluster list is described as **Table S5**.

Table S5 Format description of DEGs clustering list. (Download)

Field	Description
Gene	Gene ID
A-VS-B	log2FoldChange of A-VS-B
C-VS-D	log2FoldChange of C-VS-D
...	...
...	Gene symbol, GO, Kegg and NR annotation

8 How to read DEG GO enrichment analysis result

Make sure that the computer has installed java and use IE browser to open GOView.html. The left navigation includes three types of GO terms for each control-treatment pairwise (C: cellular component, P: biological process, F: molecular function). Click one of them, the enriched GO terms result will be listed as **Figure S2**.

Gene Ontology term	Cluster frequency	Genome frequency of use	Corrected P-value
ribosomal subunit (view genes)	58 out of 426 genes, 13.6%	183 out of 15635 genes, 1.2%	9.67e-44
ribosome (view genes)	60 out of 426 genes, 14.1%	226 out of 15635 genes, 1.4%	2.88e-40

Figure S2 Significantly enriched GO terms in DEGs.

Column 1 is GO term name. Column 2 is the ratio of DEGs enriched to this GO term. Column 3 is the ratio of genes enriched to this GO term in background database. Column 4 is Corrected P-value which indicates the degree of enrichment and the smaller Corrected P-value, the more significantly DEGs enriched to this GO term. The result list has been sorted by Corrected P-value.

Click the term name 'ribosomal subunit' in **Figure S2**, you can go to http://amigo.geneontology.org/amigo for more information when the computer is Internet-connected. Click 'view genes' in **Figure S2**, you can get gene IDs that enriched to this GO term as **Figure S3**.

ribosomal subunit	6122, 6202, 6224, 6187, 6181, 6235, 6193, 6138, 6125, 23521, 6135, 6218, 6137, 9349, 6217, 6134, 6139, BGI_novel_G000503, 6155, 6194, 6143, 6222, 6228, 6207, 6159, 6154, 11224, BGI_novel_G000584, 7311, 6128, 6204, BGI_novel_G000650, 6129, 6132, 6229, 6142, 6232, 10399, 4736, 6157, 6175, 6203, 6189, 25873, 6130, 6167, 6191, 6165, 6158, 6161, 6201, 6208, 6223, 9045, 6176, 6206, 6124, 6188
ribosome	6122, 6202, 6224, 6187, 6181, 6235, 6193, 6138, 6125, 23521, 6135, 6218, 6137, 9349, 6217, 6134, 6139, BGI_novel_G000503, 6155, 6194, 6143, 6222, 6228, 6207, 6159, 6154, 11224, BGI_novel_G000584, 7311, 347, 6128, 6204, BGI_novel_G000650, 6129, 6132, 6229, 6142, 6232, 10399, 4736, 6157, 6175, 6203, 6189, 25873, 6130, 6167, 6191, 6165, 6210, 6158, 6161, 6201, 6208, 9045, 6223, 6176, 6206, 6124, 6188

Figure S3 Gene ID list related to GO terms.

In the example, the following DEGs were annotated to the term 'ribosomal subunit': 6122, 6202, 6224, 6187, 6181, 6235, 6193, 6138, 6125, 23521, 6135, 6218, 6137, 9349, 6217, 6134, 6139, BGI_novel_G000503, 6155, 6194, 6143, 6222, 6228, 6207, 6159, 6154, 11224, BGI_novel_G000584, 7311, 6128, 6204, BGI_novel_G000650, 6129, 6132, 6229, 6142, 6232, 10399, 4736, 6157, 6175, 6203, 6189, 25873, 6130, 6167, 6191, 6165, 6158, 6161, 6201, 6208, 6223, 9045, 6176, 6206, 6124, 6188.

9 How to read DEG pathway enrichment analysis result

© 2019 BGI All Rights Reserved.

Open html report for pathway enrichment result and the enriched KEGG pathways will be listed as **Figure S4**.

1. sample3-VS-sample4

#	Pathway	DEGs with pathway annotation (1432)	All genes with pathway annotation (17252)	Pvalue	Qvalue	Pathway ID
1	Pathways in cancer	81 (5.66%)	531 (3.08%)	5.562454e-08	1.074132e-05	ko05200
2	Focal adhesion	74 (5.17%)	475 (2.75%)	8.877128e-08	1.074132e-05	ko04510
3	Leukocyte transendothelial migration	46 (3.21%)	280 (1.62%)	5.86161e-06	3.950743e-04	ko04670
4	Rheumatoid arthritis	25 (1.75%)	115 (0.67%)	6.530153e-06	3.950743e-04	ko05323
5	Malaria	19 (1.33%)	76 (0.44%)	1.00329e-05	4.855924e-04	ko05144

Figure S4 Pathway enrichment analysis of DEGs.

Column 1 is ordinal number. Column 2 is pathway name. Column 3 is the ratio of DEGs enriched to this pathway. Column 4 is the ratio of genes enriched to this pathway in background database. Pvalue and Qvalue are both values that indicate the degree of enrichment and Qvalue is corrected Pvalue. The smaller they are, the more significantly DEGs enriched to this pathway. The result list has been sorted by Qvalue. The last column pathway ID is corresponding to pathway name.

Click pathway name 'Leukocyte transendothelial migration' in **Figure S4**, you can get gene IDs that enriched to it as **Figure S5**.

3	Leukocyte transendothelial migration	146850, 654463, 5909, 4318, 1364, 402415, 3383, 2888, 100528016, 5175, 9404, 149461, 285590, 5880, 50507, 79778, 58494, 8572, 8481, 6525, 5603, 90799, 55691, 100506649, 29970, 4739, 6876, 55679, 5010, 9076, 9411, 26509, 9758, 10398, 8727, 7412, 7070, 6387, 8502, 7430, 7414, 71, 60, 4771, 80014, 51306
4	Rheumatoid arthritis	2921, 6364, 6374, 3576, 3553, 4319, 2920, 2919, 3552, 4314, 2353, 4312, 3589, 100288077, 3383, 7099, 7422, 1514, 7040, 533, 7042, 6387, 284, 5157, 6347

Figure S5 Gene ID list related to pathway.

There are 46 DEGs enriched to the pathway 'Leukocyte transendothelial migration'.

Furthermore, detecting the most significant pathways, the enrichment analysis of ***DEG*** pathway significance, allows us to see detailed pathway information in KEGG database. For example, clicking the hyperlink on 'Leukocyte transendothelial migration' in **Figure S5** will get detailed information as shown in **Figure S6**.

© 2019 BGI All Rights Reserved.

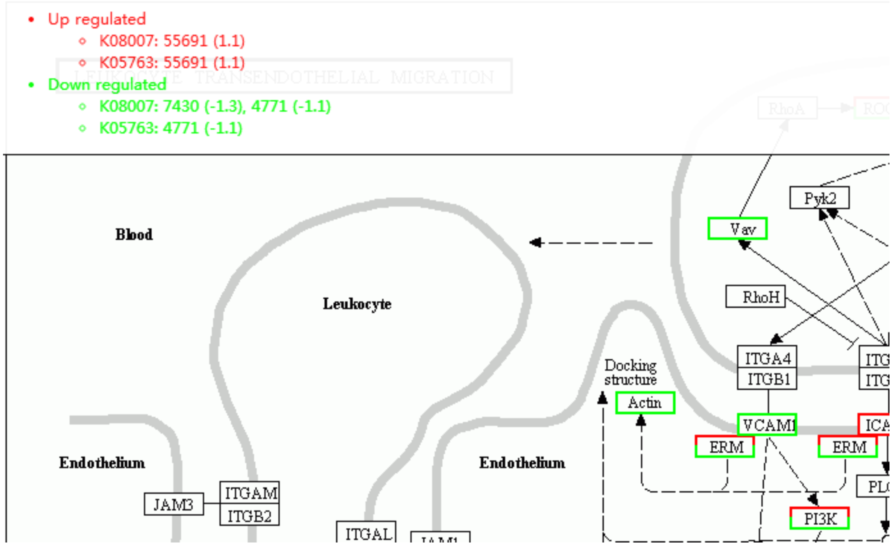

Figure S6 An example of KEGG pathway of 'Leukocyte transendothelial migration'.

Up-regulated genes are marked with red borders and down-regulated genes with green borders. Non-change genes are marked with black borders. When mouse hover on border with red or green, the related DEGs appear on the top left. Clicking gene name in the figure, the page will redirect to KEGG website if the computer is Internet-connected.

10 TF

In molecular biology and genetics, a transcription factor (sometimes called a sequence-specific DNA-binding factor) is a protein that binds to specific DNA sequences, thereby controlling the rate of transcription of genetic information from DNA to messenger RNA. Transcription factors perform this function alone or with other proteins in a complex, by promoting (as an activator), or blocking (as a repressor) the recruitment of RNA polymerase (the enzyme that performs the transcription of genetic information from DNA to RNA) to specific genes. See wiki for detail https://en.wikipedia.org/wiki/Transcription_factor .

Table S6 TF format description (Download)

Item	Description
GeneID	Gene ID
Length	Gene length
Sample1-Expression	Expression level of sample1
Sample2-Expression	Expression level of sample2
log2FoldChange(Sample2/Sample1)	log2 transformed fold change between sample1 and sample2
up/down	Up regulation / down regulation
Transcripts	Transcripts
TF_family	TF family
Included_domain	Included domain (for plant)
Excluded_domain Excluded domain (for plant)	
PlntfDB_link/AnimalDB_link	Linkage for the database
...	Gene symbol, GO, Kegg and NR annotation

© 2019 BGI All Rights Reserved.

FAQs

1. How to upload the RNA data onto NCBI?

See guidance on website: https://www.ncbi.nlm.nih.gov/guide/howto/submit-sequence-data/

2. What is Q20 and Q30? How about the quality of my data?

Q scores are defined as a property that is logarithmically related to the base calling error probabilities (P)[2], $Q = -10\log_{10}P$. If Phred assigns a Q score of 30 (Q30) to a base, this is equivalent to the probability of an incorrect base call 1 in 1000 times. This means that the base call accuracy (i.e., the probability of a correct base call) is 99.9%. If base call accuracy of 99% (Q20) will have an incorrect base call probability of 1 in 100. Q20 represents the percentage of the bases which Q score are more than 20. Q30 represents the percentage of the bases which Q score are more than 30. For example, if we sequenced 1Gb data in total, the Q score of 0.9Gb bases is no less than 20 , then we can say the Q20 is 90%. Normally, for NGS sequencing, Q20≥90%, Q30≥80% can be defined as good quality data.

3. What's the differentce between FPKM and RPKM?

RPKM stands for Reads Per Kilobase of transcript per Million mapped reads. In RNA-Seq, the relative expression of a transcript is proportional to the number of cDNA fragments that originate from it. RPKM=(1000000*C)/(N*L/1000), C represents the amount of reads which mapped to the specific transcripts, N represents the amount of reads which mapped to any transcripts. L represents the base amount of the specific transcripts.

FPKM stands for Fragments Per Kilobase of transcript per Million mapped reads. FPKM=(1000000*C)/(N*L/1000), C represents the amount of fragment which mapped to the specific transcripts, N represents the amount of fragment which mapped to any transcripts. L represents the base amount of the specific transcripts.

In RNA-Seq, the relative expression of a transcript is proportional to the number of cDNA fragments that originate from it. Paired-end RNA-Seq experiments produce two reads per fragment, but that doesn't necessarily mean that both reads will be mappable. For example, the second read is of poor quality. If we were to count reads rather than fragments, we might double-count some fragments but not others, leading to a skewed expression value. Thus, FPKM is calculated by counting fragments, not reads.

4. Once I get the sequencing data, how can I open those files? How can I do the analysis for selected the interested genes?

You can use EditPlus to open the files. Such as water channel protein, you can search for the genes according to the keywords (AQP, aquaporin, etc.). After obtaining the gene ID, you can search for the gene ID in the sequence result file to get the gene sequence.

5. How to select the genes for qPCR validation? How do we evaluate the validation result?

For RNA-Seq, thousands of differentially expressed genes are identified. We need to select the genes for qPCR validation by our own research interests. Normally, we need to select no less than 20 genes which are highly expressed and significantly differentially expressed for validation.

6. Do we need to do the biological replicates when doing the RNA seq? How many replicates per samples is recommended?

Yes, we recommend 3 biological replicates per sample for RNA seq which is more sufficient for bioinformatics analysis.

7. For library construction, why we fragment the RNA instead of cDNA?

Pls refer to paper 'RNA-Seq: a revolutionary tool for Transcriptomics'.

8. Is there some online tools for heatmap ?

www.heatmapper.ca/expression/ was an online tools for heatmap of expression. First you should follow the example table, generate your table start 'UNIQID' 'NAME' 'sample1Expression' 'sample2Expression'... .

9. Is there some online tools for Functional annotation ?

The Database for Annotation, Visualization and Integrated Discovery (DAVID)(https://david.ncifcrf.gov/) was a free and powerful tools for annotation. It provides a comprehensive set of functional annotation tools for investigators to understand biological meaning behind large list of genes.

10. How to use DAVID ?

Enter the webpage https://david.ncifcrf.gov/summary.jsp; Step 1: Enter Gene List; Step 2: Select Identifier; Step 3: List Type; Step 4: Submit List. And Wait the annotation result.

References

[1] Cock, P. J. et al. The Sanger FASTQ file format for sequences with quality scores, and the Solexa/Illumina FASTQ variants. Nucleic Acids Res. 38, 1767-1771 (2010).
[2] Kim, D., Langmead, B. & Salzberg, S. L. HISAT: a fast spliced aligner with low memory requirements. Nat. Methods 12, 357-360 (2015).
[3] Pertea, M. et al. StringTie enables improved reconstruction of a transcriptome from RNA-seq reads. Nat. Biotechnol. 33, 290-295 (2015).
[4] Trapnell, C. et al. Differential gene and transcript expression analysis of RNA-seq experiments with TopHat and Cufflinks. Nat. Protoc. 7, 562-578 (2012).

© 2019 BGI All Rights Reserved.

[5] Kong, L. et al. CPC: assess the protein-coding potential of transcripts using sequence features and support vector machine. Nucleic Acids Res. 35, W345-W349 (2007).
[6] Martin, J. A. & Wang, Z. Next-generation transcriptome assembly. Nat. Rev. Genet. 12, 671-682 (2011).
[7] McKenna, A. et al. The Genome Analysis Toolkit: a MapReduce framework for analyzing next-generation DNA sequencing data. Genome Res. 20, 1297-1303 (2010).
[8] Shen, S. et al. rMATS: Robust and flexible detection of differential alternative splicing from replicate RNA-Seq data. Proc. Natl Acad. Sci. USA 111, E5593-E5601 (2014).
[9] Langmead, B. et al. Fast gapped-read alignment with Bowtie 2. Nat. Methods 9, 357-359 (2012).
[10] Li, B. & Dewey, C. N. RSEM: accurate transcript quantification from RNA-Seq data with or without a reference genome. BMC Bioinformatics 12, 323 (2011).
[11] Krzywinski, M. et al. Circos: an information aesthetic for comparative genomics. Genome Res. 19, 1639-1645 (2009).
[12] Eisen, M. B., et al. (2001). Cluster analysis and display of genome-wide expression patterns. Proc Natl Acad Sci USA, (1998)95(25): 14863-8. 2001.29: 1165-1188.
[13] M. J. L. de Hoon, et al. (2004). Open Source Clustering Software. Bioinformatics, 20(9): 1453-1454.
[14] Saldanha, A. J. (2004). Java Treeview--extensible visualization of microarray data. Bioinformatics, 20(17): 3246-8.
[15] Kumar, L. & Futschik, M. E. Mfuzz: a software package for soft clustering of microarray data. Bioinformation 2, 5-7 (2007).
[16] Audic, S. & Claverie, J. M. The significance of digital gene expression profiles. Genome Res. 7, 986-995 (1997).
[17] Mistry, J. et al. Challenges in homology search: HMMER3 and convergent evolution of coiled-coil regions. Nucleic Acids Res. 41, e121 (2013).
[18] Buchfink, B., Xie, C. & Huson, D. H. Fast and sensitive protein alignment using DIAMOND. Nat. Methods, 12, 59-60 (2015).
[19] von Mering, C. et al. STRING: known and predicted protein-protein as sociations, integrated and transferred across organisms. Nucleic Acids Res. 33, D433-D437 (2005).

2017 Copyright BGI All Rights Reserved 粤ICP备 12059600
Technical Support E-mail:info@bgitechsolutions.com
Website: www.bgitechsolutions.com

© 2019 BGI All Rights Reserved.
