# Supplementary material for: Tissue-Specific Downregulation of Fatty Acid Synthase Suppresses Intestinal Adenoma Formation via Coordinated Reprograming of Transcriptome and Metabolism in the Mouse Model of Apc-Driven Colorectal Cancer
Source: Int J Mol Sci. 2022 Jun 10;23(12):6510. doi: 10.3390/ijms23126510 (PMC9245602; doi:10.3390/ijms23126510)
Supplement: Supplementary file 1 [file ijms-23-06510-s001.zip › ijms-1768628-supplementary-1/Supplementary/Table S3.pdf]

Table S3: RPPA analysis of metabolic enzymes

FASN$^{+/D}$/Apc/Cre vs Apc/Cre Reference median

Primary.Ab	log2FC	Wilcoxon.PVal	FDR
ME2	-0.50531900617402	0.00507486809794025	0.0124919830103145
G6PD	0.356430498580574	0.00507486809794025	0.0124919830103145
KGA / GAC	0.439414694722753	0.00507486809794025	0.0124919830103145
SDHB	0.702303590868516	0.00507486809794025	0.0124919830103145
ACO1	0.232614132962274	0.00507486809794025	0.0124919830103145
GLS2	0.557444085720816	0.00507486809794025	0.0124919830103145
ACO2	0.785888535679022	0.00507486809794025	0.0124919830103145
ACLY	0.367770000806591	0.00507486809794025	0.0124919830103145
HK2	0.107876288516259	0.00507486809794025	0.0124919830103145
FASN	-0.362005562888291	0.00507486809794025	0.0124919830103145
HK3	0.713536771625705	0.00507486809794025	0.0124919830103145
FH	0.745298094705334	0.00507486809794025	0.0124919830103145
GFPT1	-0.605621577550896	0.00507486809794025	0.0124919830103145
PCK2	-0.232819427601423	0.00823901882572464	0.0175765734948792
PDH E1 alpha	-0.343598261941599	0.00823901882572464	0.0175765734948792
PDL1	0.607000883535152	0.013065226764426	0.026130453528852
MDH1	-0.122472566579598	0.0193733848500302	0.036467547952998
ME1	0.153620578168709	0.0306389879377033	0.0490223807003253
GLUT1 (SLC2A1)	0.18379152078753	0.0306389879377033	0.0490223807003253
CS	0.250831673728568	0.0306389879377033	0.0490223807003253
LDHA	0.0984590422980025	0.0567344395845372	0.0864524793669138
PYGL	-0.0987298690248952	0.0926958025578126	0.134830258265909
PFKP	-0.0465052059267563	0.173485468321478	0.241371086360317
PC	0.157066484808091	0.297953061608168	0.381379918858455
HK1	-0.0766699447353654	0.297953061608168	0.381379918858455
OGT	0.0582614140283351	0.471169998490056	0.579901536603146
PFKFB1	0.0295188278556306	0.575173531920197	0.681687148942456
OGDH	-0.0331286629474983	0.688920555804461	0.787337778062241
PCK1	-0.0225035103886047	0.936186293473059	0.99859871303793

**PFKB3**	0.0237632108882604	0.936186293473059	0.99859871303793
**PD1**	-0.0188966143642357	1	1
**GAPDH**	0	1	1

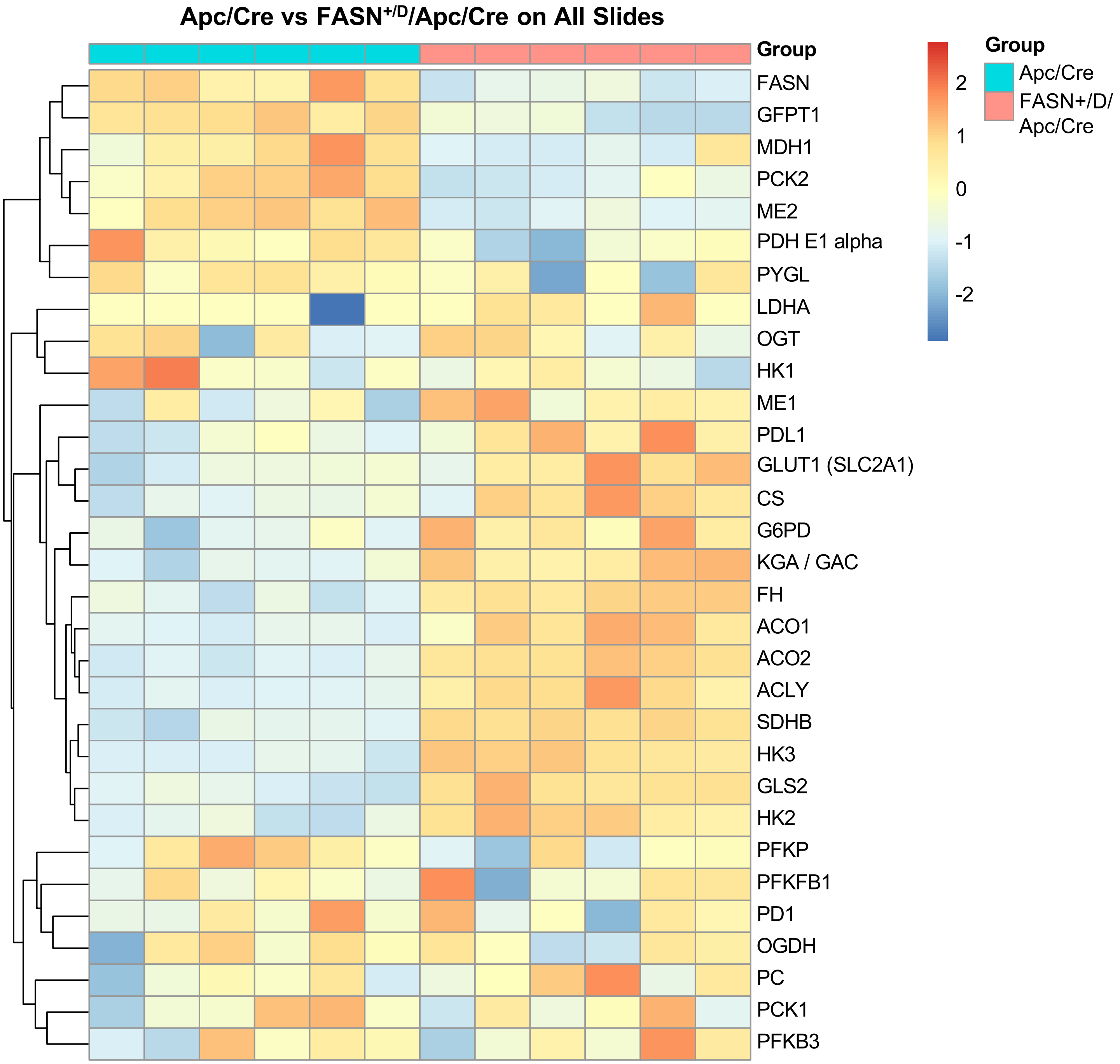
Apc/Cre vs FASN+/D/Apc/Cre on All Slides
Group
FASN
GFPT1
MDH1
PCK2
ME2
PDH E1 alpha
PYGL
LDHA
OGT
HK1
ME1
PDL1
GLUT1 (SLC2A1)
CS
G6PD
KGA / GAC
FH
ACO1
ACO2
ACLY
SDHB
HK3
GLS2
HK2
PFKP
PFKFB1
PD1
OGDH
PC
PCK1
PFKB3
2
1
0
-1
-2
Group
Apc/Cre
FASN+/D/
Apc/Cre

FASN$^{D/D}$/Apc/Cre vs Apc/Cre Reference median

Primary.Ab	log2FC	Wilcoxon.PVal	FDR
**ME1**	0.402621406688985	0.00507486809794025	0.00955269289024047
**PDH E1 alpha**	-0.407246885434538	0.00507486809794025	0.00955269289024047
**ME2**	-0.627574152923109	0.00507486809794025	0.00955269289024047
**PC**	0.547879846062042	0.00507486809794025	0.00955269289024047
**PDL1**	0.485955886621211	0.00507486809794025	0.00955269289024047
**PFKFB1**	0.307233441977374	0.00507486809794025	0.00955269289024047
**PFKP**	0.687552015595735	0.00507486809794025	0.00955269289024047
**PFKB3**	0.473853575066423	0.00507486809794025	0.00955269289024047
**SDHB**	0.774195625591252	0.00507486809794025	0.00955269289024047
**ACO1**	0.425508631393241	0.00507486809794025	0.00955269289024047
**GLS2**	0.384164697235887	0.00507486809794025	0.00955269289024047
**ACO2**	0.595359943468956	0.00507486809794025	0.00955269289024047
**GLUT1 (SLC2A1)**	0.4166593588649	0.00507486809794025	0.00955269289024047
**CS**	0.582821713293506	0.00507486809794025	0.00955269289024047
**HK2**	0.145602230168479	0.00507486809794025	0.00955269289024047
**FASN**	-1.2323789419473	0.00507486809794025	0.00955269289024047
**GFPT1**	-0.475674781267784	0.00507486809794025	0.00955269289024047
**PCK2**	-0.285428180223822	0.00715477722031697	0.0127196039472302
**HK3**	0.216474745086545	0.00823901882572464	0.0131824301211594
**FH**	0.396833916912948	0.00823901882572464	0.0131824301211594
**LDHA**	0.134157595035648	0.0103496267886546	0.0157708598684261
**G6PD**	-0.259469225719954	0.013065226764426	0.0181777068026797
**HK1**	-0.25063644236632	0.013065226764426	0.0181777068026797
**MDH1**	0.0882955270265766	0.0306389879377033	0.0408519839169377
**OGDH**	-0.249373815567096	0.0655521611655026	0.0839067662918433
**PCK1**	-0.177836178112716	0.229766270461138	0.28278925595217
**OGT**	-0.101115353195159	0.297953061608168	0.340517784695049
**KGA / GAC**	0.0837009303512897	0.297953061608168	0.340517784695049
**PD1**	0.0421623262389996	0.521109982958906	0.575017912230517

**PYGL**	-0.0361417347233068	0.575173531920197	0.61351843404821
**ACLY**	-0.00540552586228049	0.936186293473059	0.966385851327029
**GAPDH**	0	1	1

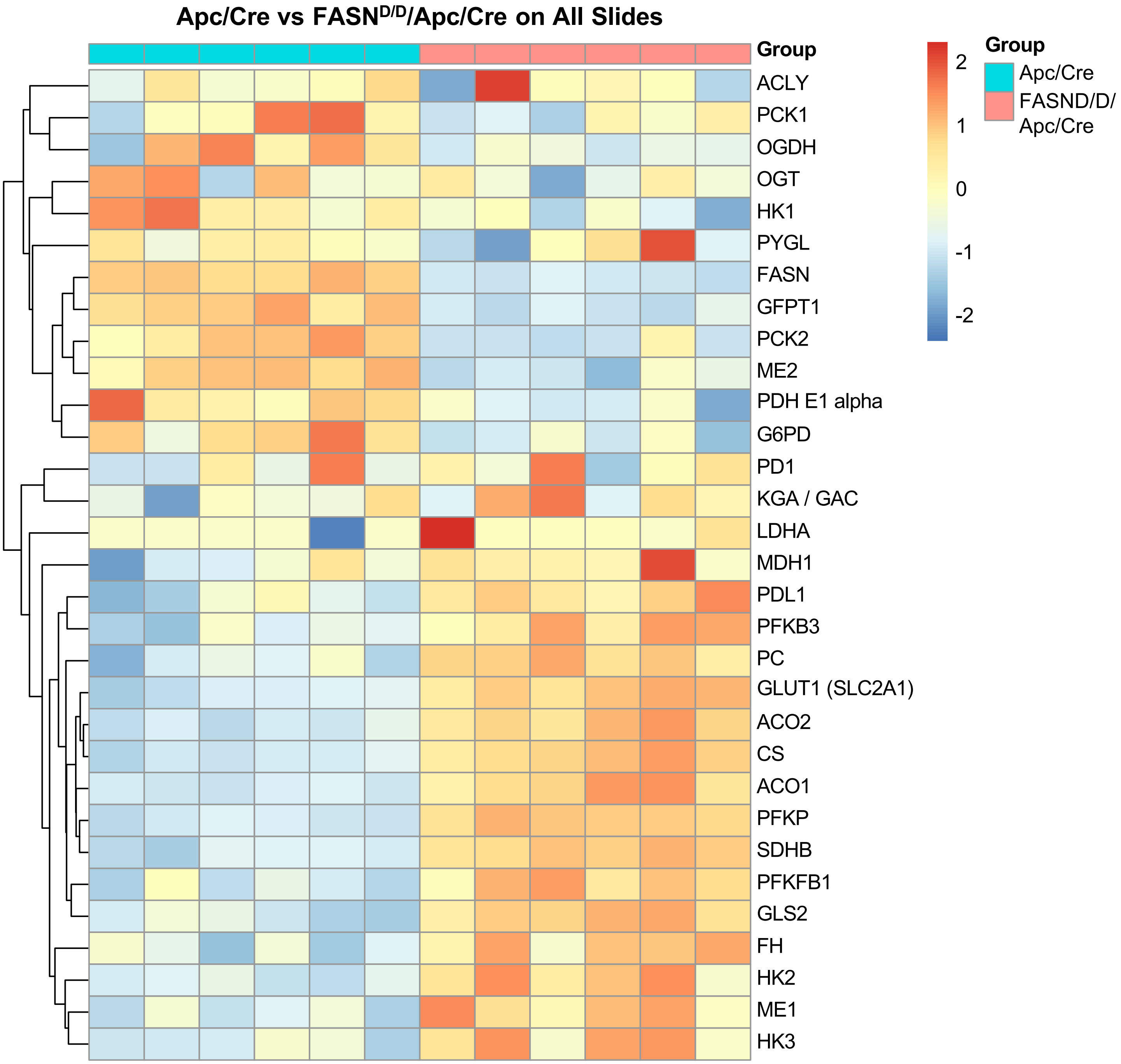
Apc/Cre vs FASN$^{D/D}$/Apc/Cre on All Slides
Group
ACLY
PCK1
OGDH
OGT
HK1
PYGL
FASN
GFPT1
PCK2
ME2
PDH E1 alpha
G6PD
PD1
KGA / GAC
LDHA
MDH1
PDL1
PFKB3
PC
GLUT1 (SLC2A1)
ACO2
CS
ACO1
PFKP
SDHB
PFKFB1
GLS2
FH
HK2
ME1
HK3
2
1
0
-1
-2
Group
Apc/Cre
FASND/D/
Apc/Cre
